# Supplementary material for: True Colors: Commercially-acquired morphological genotypes reveal hidden allele variation among dog breeds, informing both trait ancestry and breed potential
Source: PLoS One. 2019 Oct 28;14(10):e0223995. doi: 10.1371/journal.pone.0223995 (PMC6816562; doi:10.1371/journal.pone.0223995)

**S1 Fig. Allele Distribution.** Distribution of alleles for a) *TYRP1*, b) *MITF*, c) *PSMB7*, d) *RALY*, e) *KRT71*, f) *FGF5*, g) *T*, h) *BMP3*, i) chr10 ear set marker. Breeds are grouped by phylogenetic relationship.

**a)**


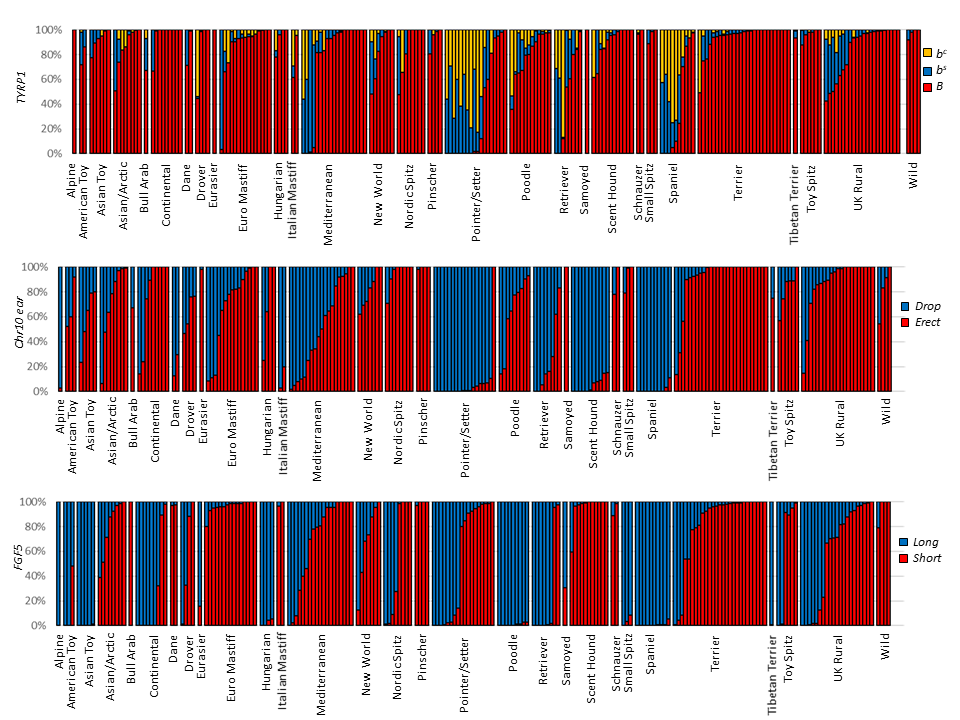


**b)**


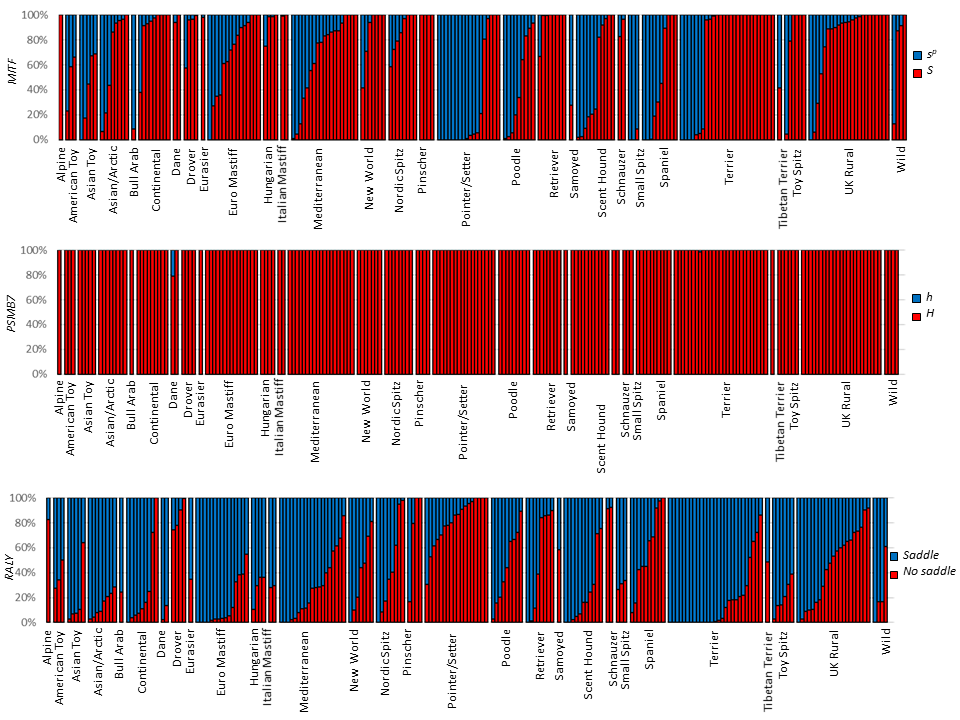


**c)**


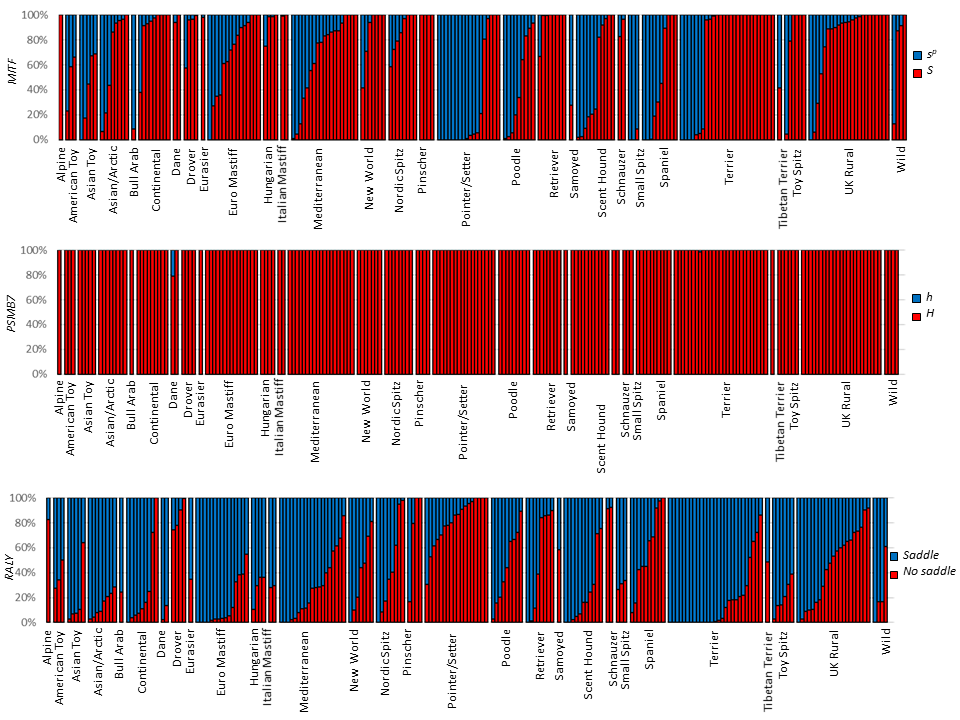


**d)**


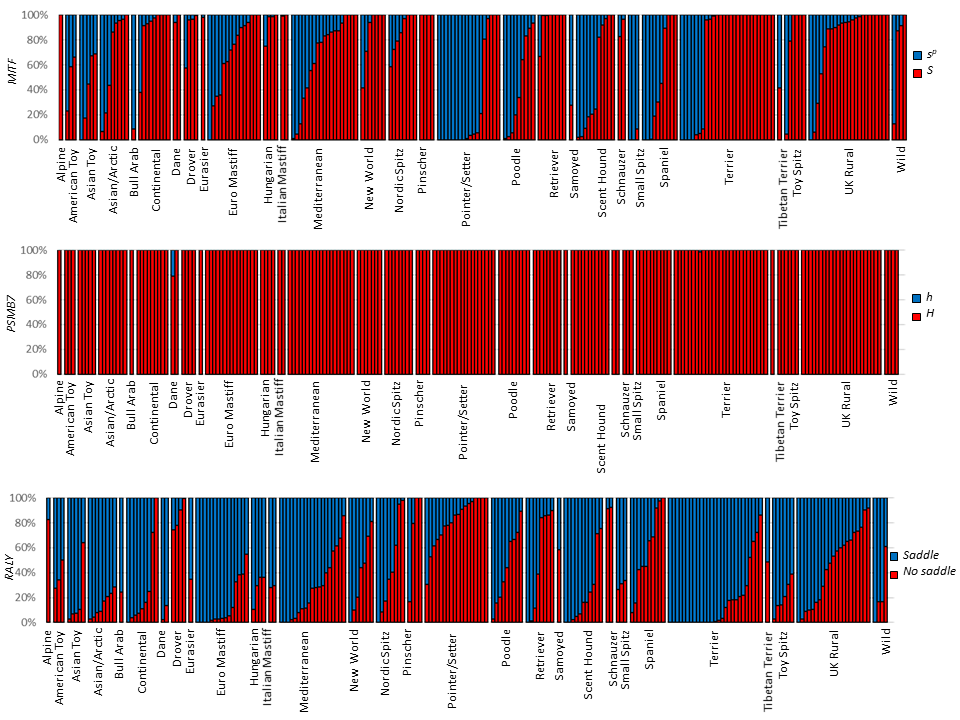


**e)**


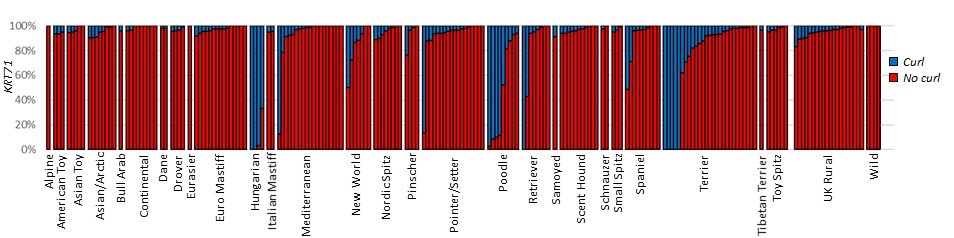


**f)**


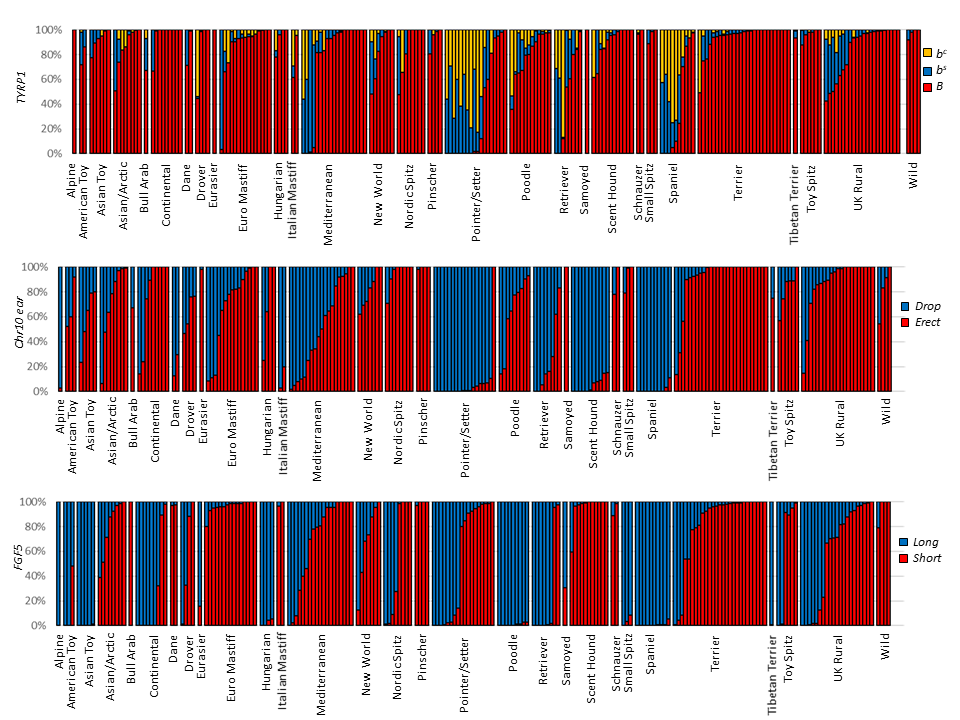


**g)**


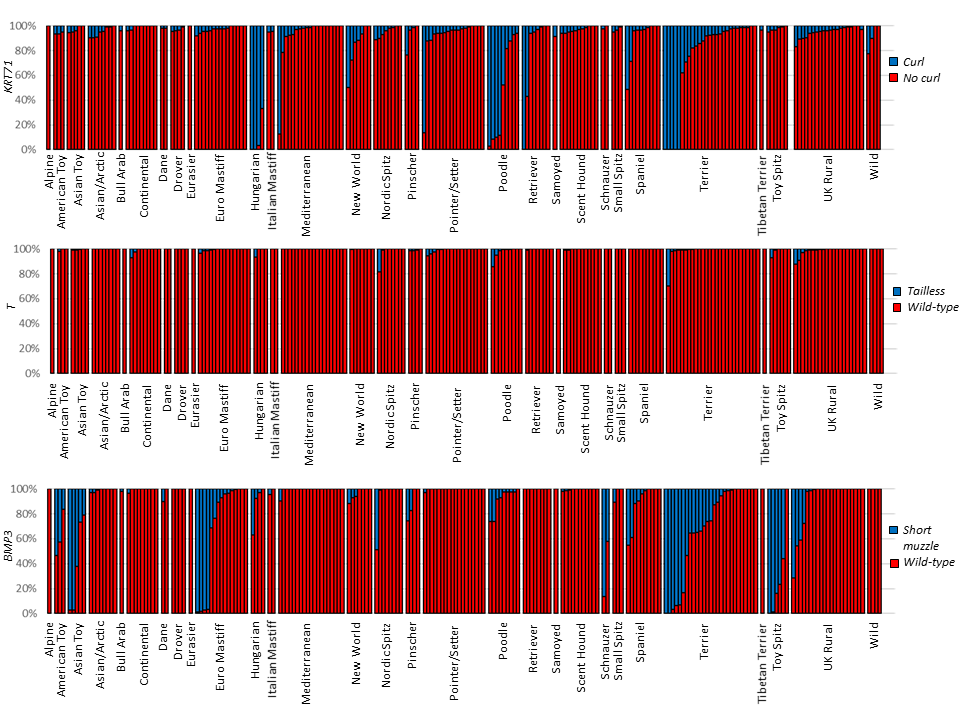


**h)**


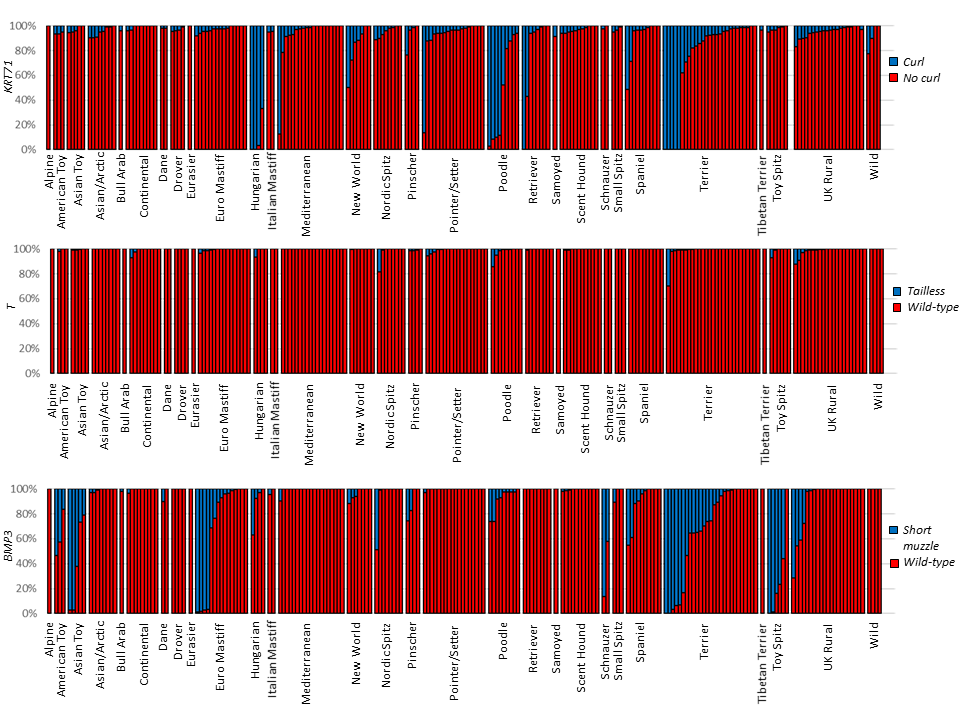


**i)**


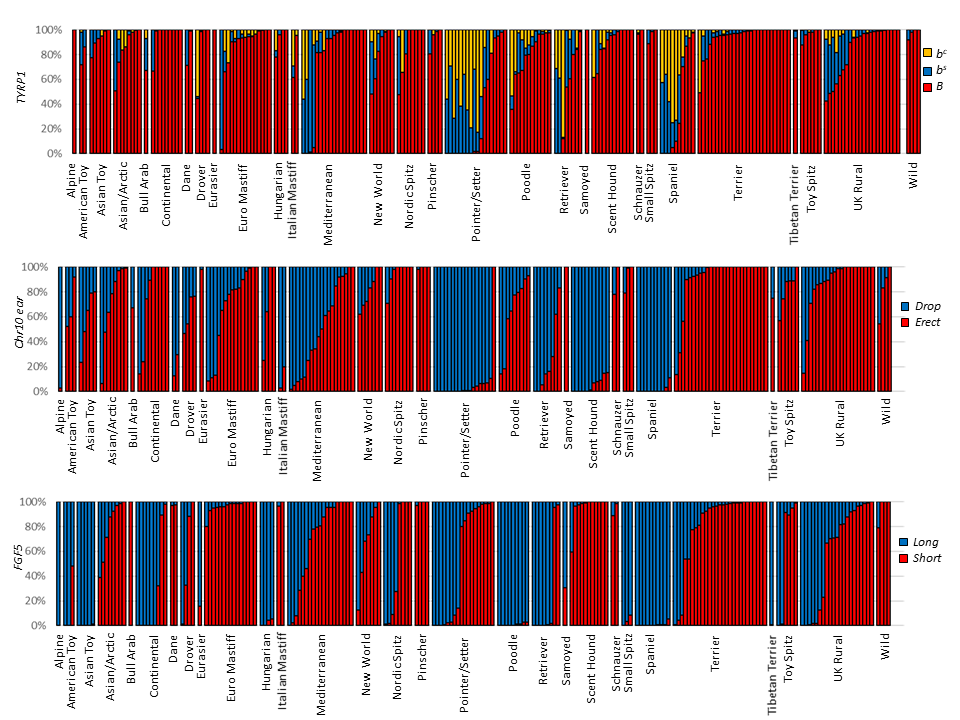

Supplement: S1 Fig — Distribution of alleles for a) TYRP1, b) MITF, c) PSMB7, d) RALY, e) KRT71, f) FGF5, g) T, h) BMP3, i) chr10 ear set marker. Breeds are grouped by phylogenetic relationship. (DOCX) [file pone.0223995.s001.docx]
