# Supplementary material for: True Colors: Commercially-acquired morphological genotypes reveal hidden allele variation among dog breeds, informing both trait ancestry and breed potential
Source: PLoS One. 2019 Oct 28;14(10):e0223995. doi: 10.1371/journal.pone.0223995 (PMC6816562; doi:10.1371/journal.pone.0223995)

**S2 Fig. Allele frequencies in breeds with multiple populations.** Allele frequencies for a) *MC1R*, b) *ASIP*, c) *CBD103*, d) *TYRP1*, e) *MITF*, f) *PSMB7*, g) *RALY*, h) *KRT71*, i) *FGF5*, j) *T*, k) *BMP3*, l) chr10 ear marker, for all breeds with multiple populations. Initial X^2^ significance (p < 0.0167) is indicated by horizontal black bars. Pairwise significance was conducted for all significant breeds with greater than two populations, with significance level indicated by **. Letters under horizontal black bar denote significant groupings.

**a)**


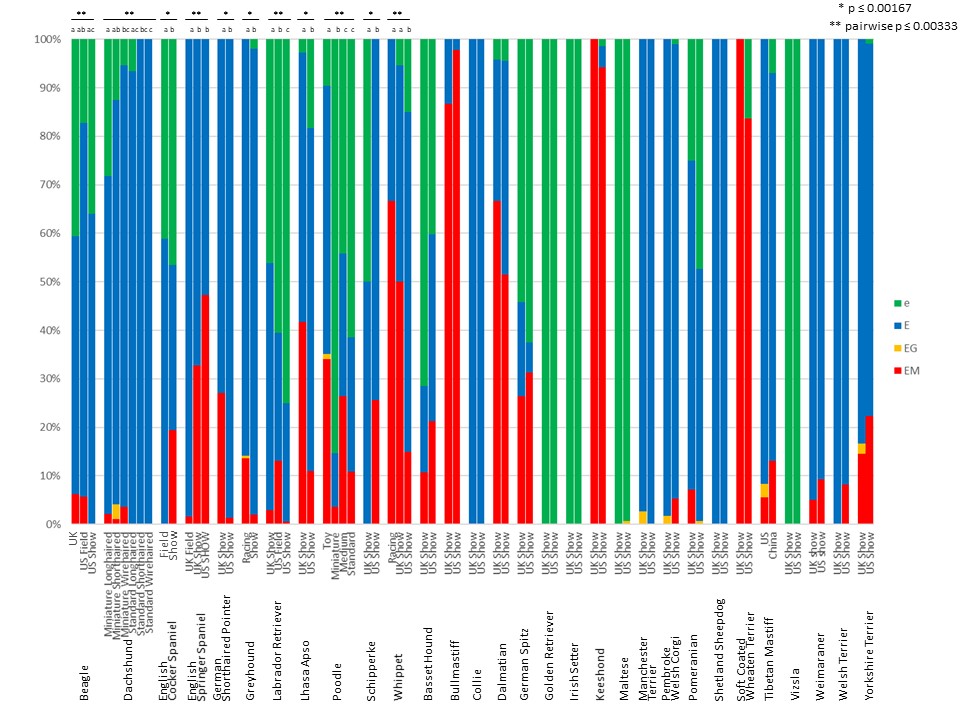


**b)**


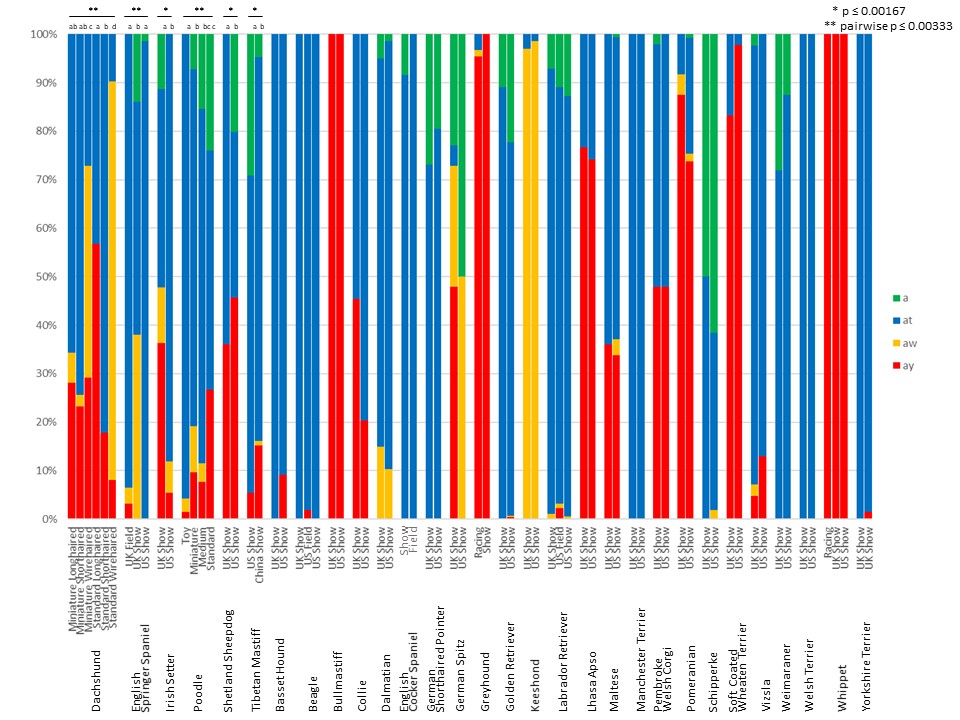


**c)**


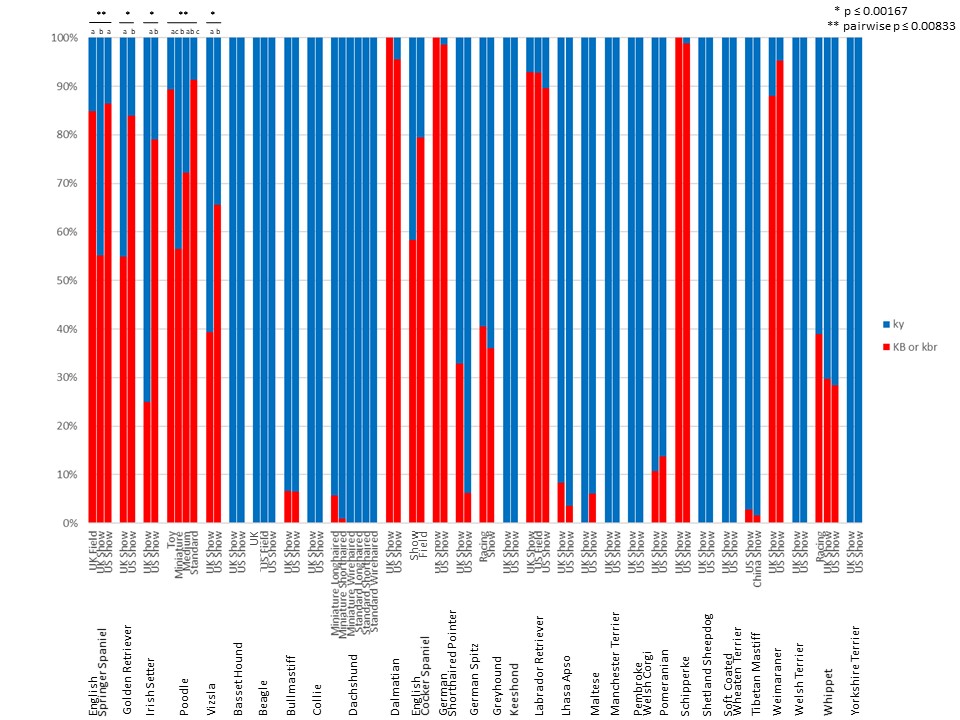


**d)**


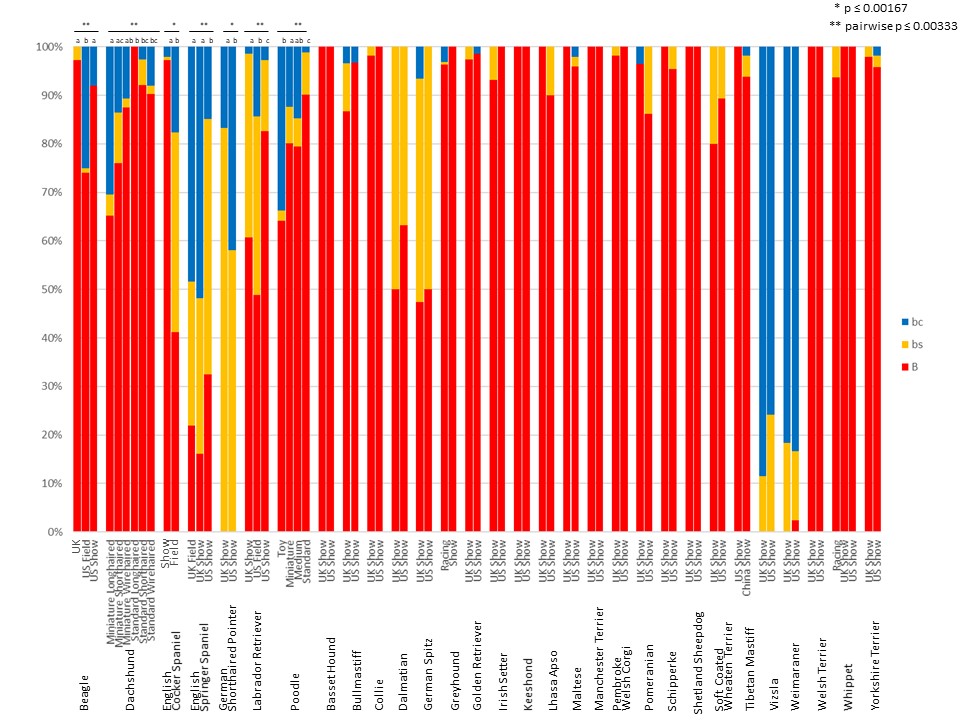


**e)**
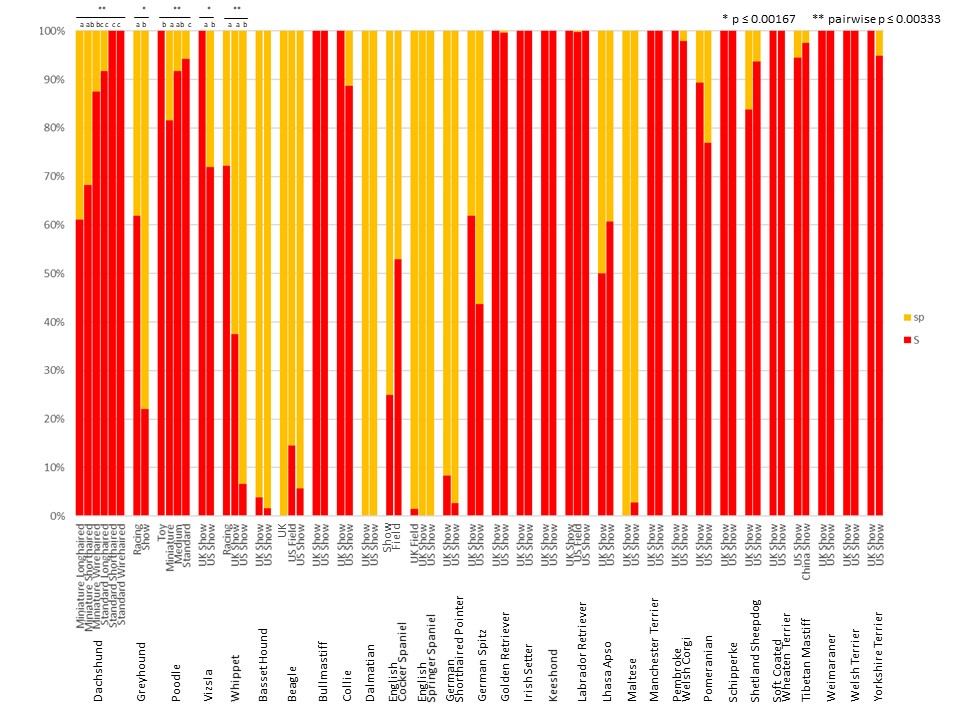


**f)**


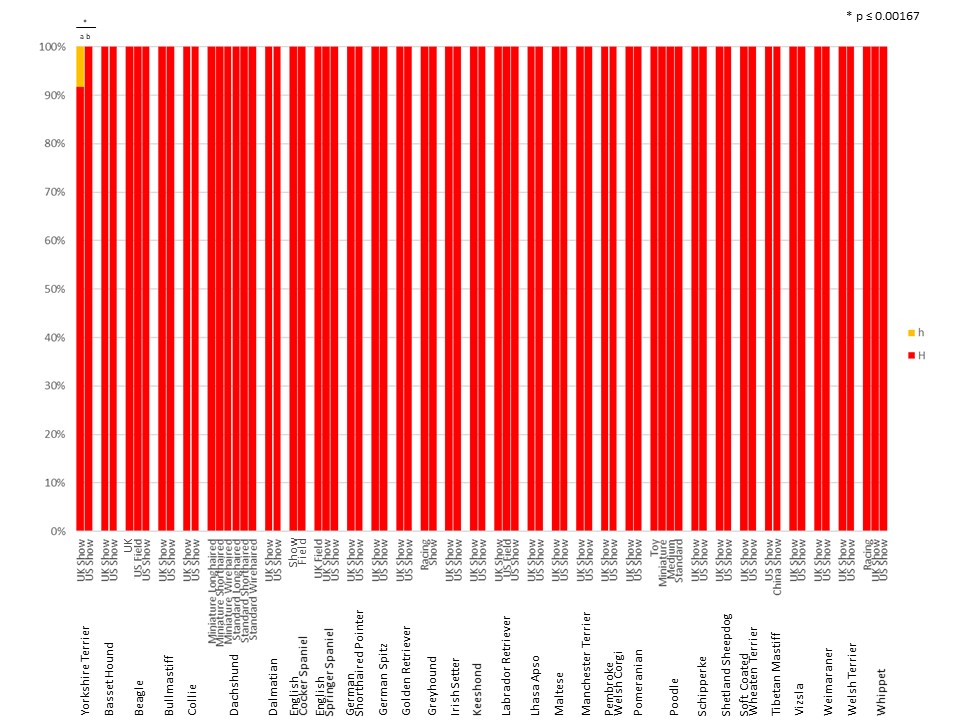


**g)**


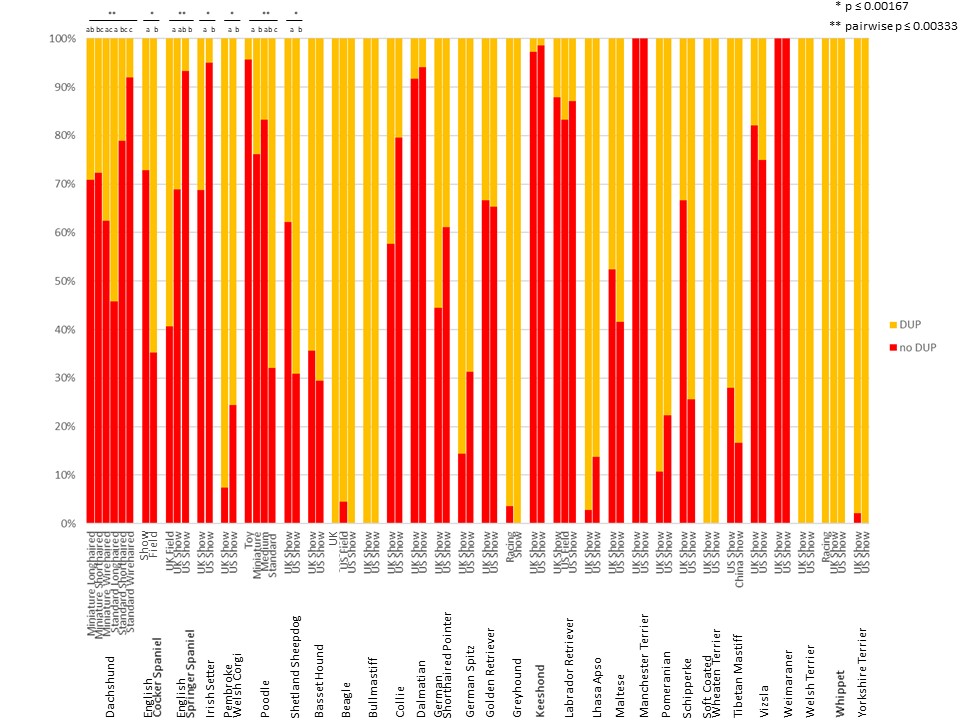


**h)**


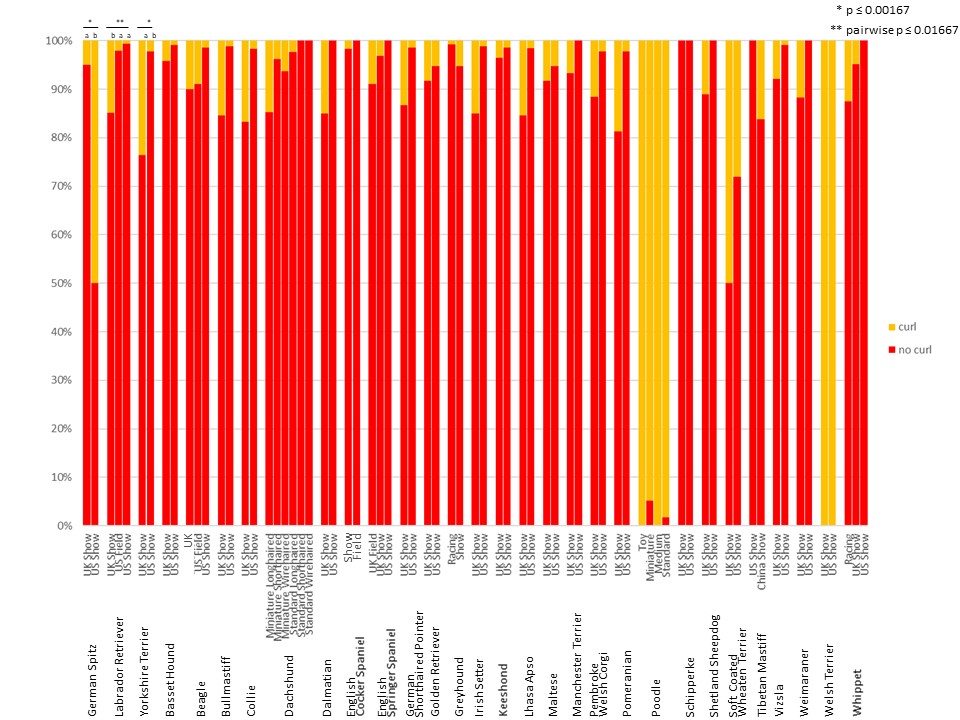


**i)**


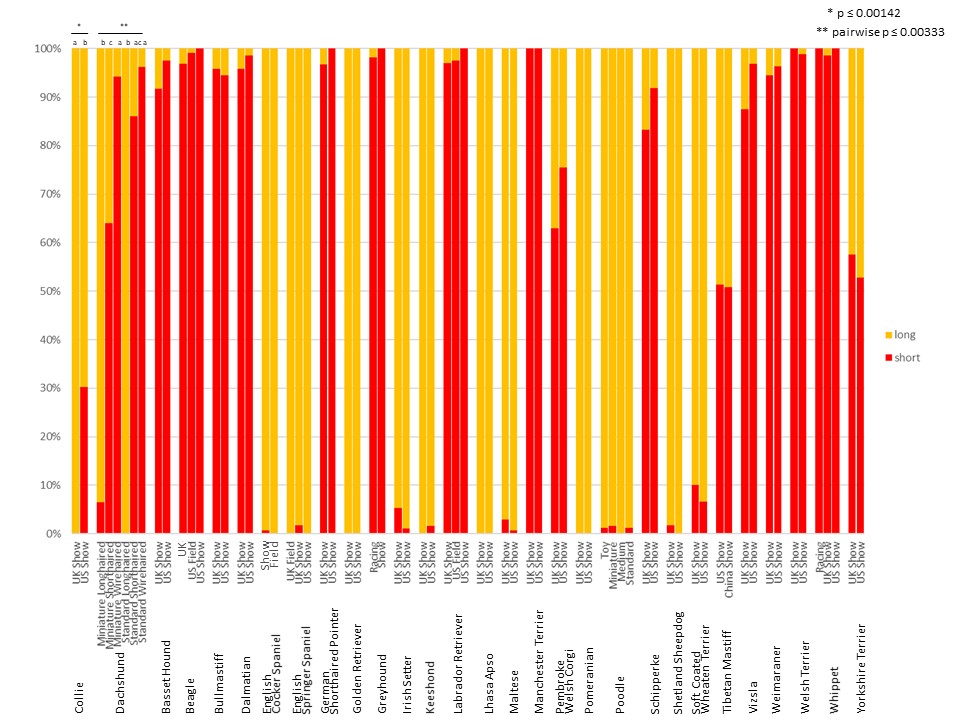


**j)**


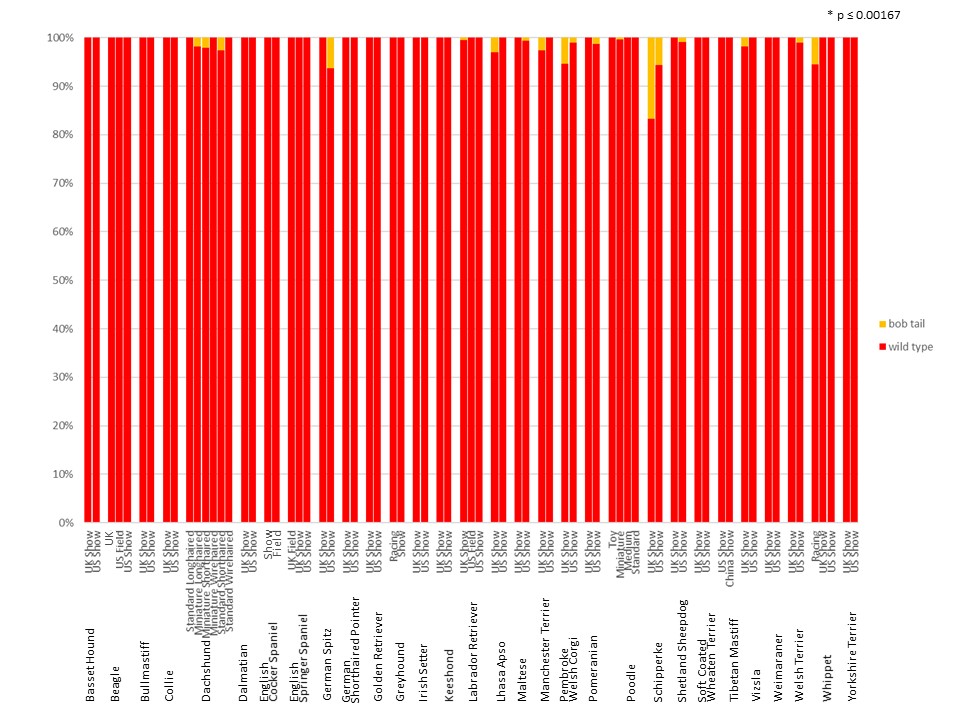


**k)**


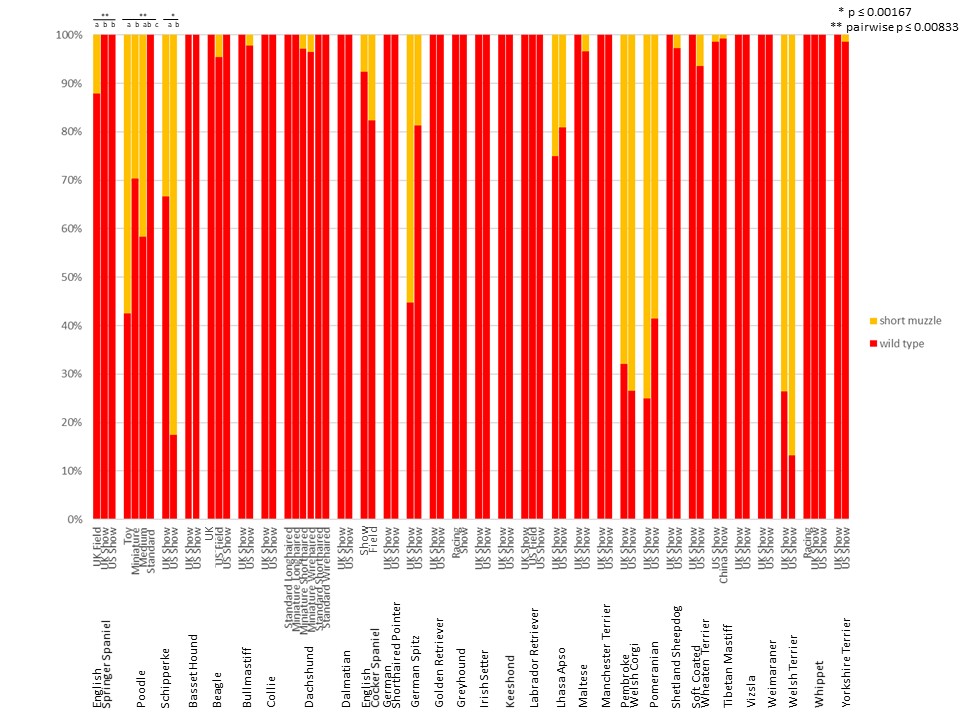


**l)**


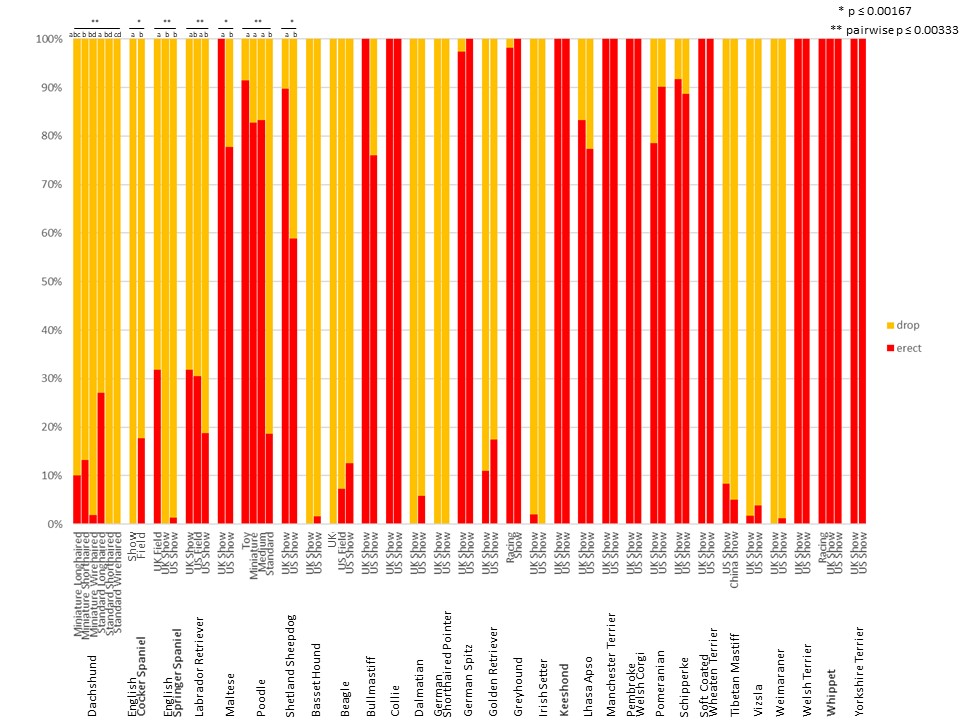

Supplement: S2 Fig — Allele frequencies for a) MC1R, b) ASIP, c) TYRP1, d) CBD103, e) MITF, f) PSMB7, g) RALY, h) KRT71, i) FGF5, j) T, k) BMP3, l) chr10 ear marker, for all breeds with multiple populations. Initial X2 significance (p < 0.0167) is indicated by horizontal black bars. Pairwise significance was conducted for all significant breeds with greater than two populations, with significance level indicated by **. Letters under horizontal black bar denote significant groupings. (DOCX) [file pone.0223995.s002.docx]
