# Supplementary material for: True Colors: Commercially-acquired morphological genotypes reveal hidden allele variation among dog breeds, informing both trait ancestry and breed potential
Source: PLoS One. 2019 Oct 28;14(10):e0223995. doi: 10.1371/journal.pone.0223995 (PMC6816562; doi:10.1371/journal.pone.0223995)
Supplement: S1 Table — List of breeds, their abbreviations used throughout the paper, and the number of samples genotyped. Each breed was assigned to a phylogenetic clade based on previously published results [32,33]. Clade names in parenthesis indicate breeds not included previously [32,33], but for which a clade was assigned based on known breed history and phenotypes. (DOCX) [file pone.0223995.s003.docx]

**S1 Table. Sample information.** List of breeds, their abbreviations used throughout the paper, and the number of samples genotyped. Each breed was assigned to a phylogenetic clade based on previously published results [32,33]. Clade names in parenthesis indicate breeds not included previously [32,33], but for which a clade was assigned based on known breed history and phenotypes.

| **Breed** | **Abbreviation** | **Clade** | **n** |
| --- | --- | --- | --- |
| Affenpinscher | AFFN | (Toy Spitz) | 40 |
| Afghan Hound | AFGH | Mediterranean | 69 |
| Airedale Terrier | AIRT | Terrier | 59 |
| Akita | AKIT | Asian/Arctic | 79 |
| Alaskan Malamute | AMAL | Asian/Arctic | 70 |
| American Bulldog | ABUL | (Euro Mastiff) | 39 |
| American Eskimo Dog | AESK | Small Spitz | 59 |
| American Foxhound | FOXH | Scent hound | 33 |
| American Staffordshire Terrier | AMST | Euro Mastiff | 297 |
| American Water Spaniel | AWSP | (Spaniel) | 34 |
| Anatolian Shepherd Dog | ANAT | Mediterranean | 27 |
| Argentine Dogo | DOGO | (Euro Mastiff) | 14 |
| Australian Cattle Dog | AUCD | UK Rural | 45 |
| Australian Kelpie | KELP | UK Rural | 60 |
| Australian Shepherd | AUSS | UK Rural | 137 |
| Australian Terrier | AUST | Terrier | 27 |
| Azawakh | AZWK | Mediterranean | 30 |
| Basset Hound (UK) | BASS (UK) | Scent hound | 14 |
| Basset Hound (US) | BASS (US) | Scent hound | 61 |
| Beagle (UK) | BEAG (UK) | Scent hound | 18 |
| Beagle (US Field) | BEAG (US Field) | Scent hound | 55 |
| Beagle (US Show) | BEAG (US Show) | Scent hound | 44 |
| Bearded Collie | BERD | UK Rural | 60 |
| Beauceron | BEAU | (Continental Shepherds) | 29 |
| Bedlington Terrier | BEDT | Terrier | 61 |
| Belgian Malinois | BMAL | Continental Shepherds | 52 |
| Belgian Sheepdog | BELS | Continental Shepherds | 46 |
| Belgian Tervuren | TERV | Continental Shepherds | 39 |
| Bergamasco | BERG | New World | 9 |
| Berger Picard | BPIC | New World | 11 |
| Bernese Mountain Dog | BMD | Alpine | 17 |
| Bichon Frise | BICH | Poodle | 82 |
| Biewer Terrier | BIEW | (Terrier) | 30 |
| Black Barb | BARB | (UK Rural) | 19 |
| Black Russian Terrier | BRTR | Drover | 36 |
| Bloodhound | BLDH | Scent hound | 44 |
| Boerboel | BOER | Euro Mastiff | 23 |
| Bolognese | BOLO | Poodle | 20 |
| Border Collie | BORD | UK Rural | 99 |
| Border Terrier | BORT | Terrier | 94 |
| Borzoi | BORZ | UK Rural | 74 |
| Boston Terrier | BOST | Euro Mastiff | 72 |
| Bouvier Des Flanders | BOUV | Continental Shepherds | 46 |
| Boxer | BOX | Euro Mastiff | 78 |
| Boykin Spaniel | BOYK | (Spaniel) | 10 |
| Bracco Italiano | BRAC | Pointer/Setter | 13 |
| Briard | BRIA | Continental Shepherds | 55 |
| Brittany | BRIT | Pointer/Setter | 53 |
| Brussels Griffon | BRUS | Toy Spitz | 49 |
| Bull Arab | BLAB | (Euro Mastiff) | 29 |
| Bull Terrier | BULT | Euro Mastiff | 63 |
| Bulldog | BULD | Euro Mastiff | 79 |
| Bullmastiff (UK) | BULM (UK) | Euro Mastiff | 15 |
| Bullmastiff (US) | BULM (US) | Euro Mastiff | 46 |
| Cairn Terrier | CAIR | Terrier | 63 |
| Canaan Dog | CAAN | (Mediterranean) | 18 |
| Cane Corso | CANE | Italian Mastiff | 46 |
| Cardigan Welsh Corgi | CARD | UK Rural | 53 |
| Catahoula Leopard Dog | LEOP | New World | 26 |
| Caucasian Shepherd | CAUC | Mediterranean | 35 |
| Cavalier King Charles Spaniel | CKCS | Spaniel | 135 |
| Central Asian Shepherd Dog | CASD | Mediterranean | 25 |
| Cesky Terrier | CESK | (Terrier) | 17 |
| Chesapeake Bay Retriever | CPBR | (Retriever) | 45 |
| Chihuahua | CHIH | American Toy | 55 |
| Chinese Crested | CRES | American Toy | 31 |
| Chinese Shar-Pei | SHAR | Asian/Arctic | 63 |
| Chinook | COOK | New World | 36 |
| Chow Chow | CHOW | Asian/Arctic | 56 |
| Cirneco dell'Etna | CIRN | Mediterranean | 16 |
| Clumber Spaniel | CLSP | (Spaniel) | 57 |
| Cocker Spaniel | ACKR | Spaniel | 84 |
| Collie (UK) | COLL (UK) | UK Rural | 27 |
| Collie (US) | COLL (US) | UK Rural | 66 |
| Coton de Tulear | COTO | Poodle | 84 |
| Curly Coated Retriever | CCRT | Retriever | 79 |
| Dachshund (Miniature Longhaired) | DACH | Scent hound | 27 |
| Dachshund (Miniature Shorthaired) | DACH | Scent hound | 52 |
| Dachshund (Miniature Wirehaired) | DACH | Scent hound | 28 |
| Dachshund (Standard Longhaired) | DACH | Scent hound | 24 |
| Dachshund (Standard Shorthaired) | DACH | Scent hound | 19 |
| Dachshund (Standard Wirehaired) | DACH | Scent hound | 31 |
| Dalmatian (UK) | DALM (UK) | Pointer/Setter | 12 |
| Dalmatian (US) | DALM (US) | Pointer/Setter | 34 |
| Dandie Dinmont Terrier | DDMT | (Terrier) | 41 |
| Dingo | DING | Wild | 12 |
| Dobermann Pinscher | DOBP | Drover | 115 |
| Dogue de Bordeaux | DDBX | Euro Mastiff | 34 |
| Eastern Coyote | COYO (Eastern) | Wild | 29 |
| English Cocker Spaniel (Field) | ECKR (Field) | Spaniel | 17 |
| English Cocker Spaniel (Show) | ECKR (Show) | Spaniel | 72 |
| English Setter | ESET | Pointer/Setter | 63 |
| English Springer Spaniel (UK Field) | ESSP (UK Field) | Spaniel | 33 |
| English Springer Spaniel (UK Show) | ESSP (UK Show) | Spaniel | 29 |
| English Springer Spaniel (US Show) | ESSP (US Show) | Spaniel | 37 |
| Eurasier | EURA | Eurasier | 26 |
| Field Spaniel | FIEL | Spaniel | 42 |
| Finnish Lapphund | FLAP | (Nordic Spitz) | 26 |
| Flat Coated Retriever | FCR | Retriever | 82 |
| French Bulldog | FBUL | Euro Mastiff | 68 |
| German Pinscher | GPIN | (Pinscher) | 25 |
| German Shepherd Dog | GSD | New World | 162 |
| German Shorthaired Pointer (UK) | GSHP (UK) | Pointer/Setter | 37 |
| German Shorthaired Pointer (US) | GSHP (US) | Pointer/Setter | 37 |
| German Spitz (UK) | GSPZ (UK) | (Nordic Spitz) | 38 |
| German Spitz (US) | GSPZ (US) | (Nordic Spitz) | 8 |
| German Wirehaired Pointer | GWHP | Pointer/Setter | 33 |
| Giant Schnauzer | GSNZ | Drover | 44 |
| Glen of Imaal Terrier | GLEN | Terrier | 15 |
| Golden Retriever (UK) | GOLD (UK) | Retriever | 41 |
| Golden Retriever (US) | GOLD (US) | Retriever | 143 |
| Gordon Setter | GORD | Pointer/Setter | 45 |
| Great Dane | DANE | Dane | 88 |
| Great Pyrenees | GPYR | Mediterranean | 52 |
| Grey Wolf | WOLF | Wild | 12 |
| Greyhound (Racing) | GREY (Racing) | UK Rural | 84 |
| Greyhound (Show) | GREY (Show) | UK Rural | 25 |
| Harrier | HARR | (Scent hound) | 33 |
| Havanese | HAVA | Poodle | 61 |
| Ibizan Hound | IBIZ | Mediterranean | 36 |
| Icelandic Sheepdog | ICES | Nordic Spitz | 29 |
| Irish Red and White Setter | RWST | (Pointer/Setter) | 10 |
| Irish Setter (UK) | ISET (UK) | Pointer/Setter | 25 |
| Irish Setter (US) | ISET (US) | Pointer/Setter | 50 |
| Irish Terrier | IRIT | Terrier | 33 |
| Irish Water Spaniel | IWSP | Retriever | 32 |
| Irish Wolfhound | IWOF | UK Rural | 65 |
| Italian Greyhound | ITGY | UK Rural | 69 |
| Italian Spinone | SPIN | Pointer/Setter | 54 |
| Japanese Chin | CHIN | Asian Toy | 50 |
| Japanese Shiba Inu | SHIB | Asian/Arctic | 65 |
| Japanese Spitz | JSPZ | (Small Spitz) | 21 |
| Keeshond (UK) | KEES (UK) | Nordic Spitz | 18 |
| Keeshond (US) | KEES (US) | Nordic Spitz | 36 |
| Kerry Blue Terrier | KERY | Terrier | 41 |
| Komondor | KOMO | Hungarian | 26 |
| Koolie | KOOL | (UK Rural) | 42 |
| Korean Jindo | JIND | (Asian/Arctic) | 11 |
| Kritikos Lagonikos | KKLG | (Mediterranean) | 43 |
| Kuvasz | KUVZ | Mediterranean | 15 |
| Labrador Retriever (UK) | LAB (UK) | Retriever | 107 |
| Labrador Retriever (US Field) | LAB (US Field) | Retriever | 180 |
| Labrador Retriever (US Show) | LAB (US Show) | Retriever | 93 |
| Lagotto Romagnolo | LAGO | Pointer/Setter | 139 |
| Lakeland Terrier | LAKE | (Terrier) | 45 |
| Lancashire Heeler | LANC | (UK Rural) | 11 |
| Large Munsterlander | LMUN | Pointer/Setter | 21 |
| Lhasa Apso (UK) | LHAS (UK) | Asian Toy | 18 |
| Lhasa Apso (US) | LHAS (US) | Asian Toy | 42 |
| Lowchen | LOWC | (Poodle) | 37 |
| Magyar Agar | MGAG | (Mediterranean) | 24 |
| Maltese (UK) | MALT (UK) | Poodle | 21 |
| Maltese (US) | MALT (US) | Poodle | 74 |
| Manchester Terrier (UK) | MANT (UK) | Pinscher | 19 |
| Manchester Terrier (US) | MANT (US) | Pinscher | 16 |
| Maremma Sheepdog | MARM | Mediterranean | 22 |
| Mastiff | MAST | Euro Mastiff | 59 |
| McNab | MCNB | (UK Rural) | 28 |
| Mi-ki | MIKI | (Asian Toy) | 22 |
| Miniature Bull Terrier | MBLT | Euro Mastiff | 28 |
| Miniature Pinscher | MPIN | Pinscher | 52 |
| Miniature Schnauzer | MSNZ | Schnauzer | 85 |
| Mudi | MUDI | (Hungarian) | 39 |
| Neapolitan Mastiff | NEAP | Italian Mastiff | 39 |
| Newfoundland | NEWF | Retriever | 66 |
| Norfolk Terrier | NORF | Terrier | 52 |
| Norwegian Buhund | NBUH | (Nordic Spitz) | 12 |
| Norwegian Elkhound | NELK | Nordic Spitz | 91 |
| Norwegian Lundehund | NLUN | (Nordic Spitz) | 17 |
| Norwich Terrier | NOWT | Terrier | 61 |
| Nova Scotia Duck Tolling Retriever | NSDT | Retriever | 38 |
| Old English Sheepdog | OES | UK Rural | 68 |
| Otterhound | OTTR | Scent hound | 34 |
| Papillon | PAPI | Toy Spitz | 81 |
| Parson Russell Terrier | PRUS | Terrier | 124 |
| Pekingese | PEKE | Asian Toy | 58 |
| Pembroke Welsh Corgi (UK) | PEMB (UK) | UK Rural | 28 |
| Pembroke Welsh Corgi (US) | PEMB (US) | UK Rural | 47 |
| Peruvian Inca Orchid | PERU | New World | 21 |
| Petite Basset Griffon Vendeen | PBGV | Scent hound | 47 |
| Pharaoh Hound | PHAR | Mediterranean | 21 |
| Plott | PLOT | (Scent hound) | 25 |
| Podenco Canario | PDCN | (Euro Mastiff) | 20 |
| Pointer | PNTR | (Pointer/Setter) | 78 |
| Polish Greyhound | POLG | (Mediterranean) | 23 |
| Polish Lowland Sheepdog | PONS | (Continental) | 21 |
| Pomeranian (UK) | POM (UK) | Toy Spitz | 14 |
| Pomeranian (US) | POM (US) | Toy Spitz | 76 |
| Poodle (Medium) | MePOO | Poodle | 18 |
| Poodle (Miniature) | MPOO | Poodle | 130 |
| Poodle (Standard) | SPOO | Poodle | 86 |
| Poodle (Toy) | TPOO | Poodle | 47 |
| Portuguese Water Dog | PTWD | Poodle | 50 |
| Presa Canario | PRES | (Euro Mastiff) | 30 |
| Pug | PUG | Toy Spitz | 59 |
| Puli | PULI | Hungarian | 39 |
| Pumi | PUMI | Hungarian | 51 |
| Pyrenean Shepherd | PYRS | (Continental) | 14 |
| Redbone Coonhound | RBCH | Scent hound | 9 |
| Rhodesian Ridgeback | RHOD | Dane | 64 |
| Rottweiler | ROTT | Drover | 118 |
| Russell Terrier | RUSS | Terrier | 89 |
| Saluki | SALU | Mediterranean | 117 |
| Samoyed | SAMO | Samoyed | 18 |
| Schipperke (UK) | SKIP (UK) | Toy Spitz | 6 |
| Schipperke (US) | SKIP (US) | Toy Spitz | 44 |
| Scottish Deerhound | DEER | UK Rural | 38 |
| Scottish Terrier | SCOT | Terrier | 71 |
| Sealyham Terrier | SEAL | (Terrier) | 29 |
| Shetland Sheepdog (UK) | SSHP (UK) | UK Rural | 34 |
| Shetland Sheepdog (US) | SSHP (US) | UK Rural | 56 |
| Shih Tzu | SHIH | Asian Toy | 90 |
| Siberian Husky | HUSK | Asian/Arctic | 115 |
| Silky Terrier | SILK | Terrier | 46 |
| Skye Terrier | SKYE | (Terrier) | 11 |
| Sloughi | SLOU | Mediterranean | 21 |
| Small Munsterlander | SMUN | (Pointer/Setter) | 8 |
| Smooth Fox Terrier | SFOX | (Terrier) | 47 |
| Soft Coated Wheaten Terrier (UK) | SCWT (UK) | Terrier | 5 |
| Soft Coated Wheaten Terrier (US) | SCWT (US) | Terrier | 47 |
| Spanish Greyhound | GALG | (UK Rural) | 25 |
| Spanish Water Dog | SPWD | (Poodle) | 14 |
| Staffordshire Bull Terrier | STAF | Euro Mastiff | 74 |
| Standard Schnauzer | SSNZ | Schnauzer | 32 |
| Sussex Spaniel | SUSX | (Spaniel) | 18 |
| Swedish Vallhund | SVAL | Nordic Spitz | 38 |
| Taigan | TAIG | (Mediterranean) | 22 |
| Tenterfield Terrier | TENT | (Terrier) | 10 |
| Tibetan Mastiff (China) | TIBM (China) | Asian/Arctic | 61 |
| Tibetan Mastiff (US) | TIBM (US) | Asian/Arctic | 36 |
| Tibetan Spaniel | TIBS | Asian Toy | 49 |
| Tibetan Terrier | TIBT | Tibetan Terrier | 69 |
| Toy Manchester Terrier | TMNT | Pinscher | 39 |
| Treeing Walker Coonhound | TREE | (Scent hound) | 45 |
| Vizsla (UK) | VIZS (UK) | Pointer/Setter | 28 |
| Vizsla (US) | VIZS (US) | Pointer/Setter | 64 |
| Volpino Italiano | VPIN | Small Spitz | 19 |
| Weimaraner (UK) | WEIM (UK) | Pointer/Setter | 21 |
| Weimaraner (US) | WEIM (US) | Pointer/Setter | 43 |
| Welsh Springer Spaniel | WSSP | Spaniel | 54 |
| Welsh Terrier (UK) | WELT (UK) | (Terrier) | 17 |
| Welsh Terrier (US) | WELT (US) | (Terrier) | 49 |
| West Highland White Terrier | WHWT | Terrier | 77 |
| Western Coyote | COYO (Western) | Wild | 19 |
| Whippet (Racing) | WHIP (Racing) | UK Rural | 9 |
| Whippet (UK) | WHIP (UK) | UK Rural | 37 |
| Whippet (US) | WHIP (US) | UK Rural | 30 |
| White Swiss Shepherd | BBLS | (New World) | 36 |
| Wire Fox Terrier | WFOX | Terrier | 31 |
| Wirehaired Pointing Griffon | WHPG | Pointer/Setter | 20 |
| Wirehaired Vizsla | WVIZ | (Pointer/Setter) | 17 |
| Xoloitzcuintli | XOLO | New World | 17 |
| Yorkshire Terrier (UK) | YORK (UK) | Terrier | 24 |
| Yorkshire Terrier (US) | YORK (US) | Terrier | 107 |
