# Supplementary material for: True Colors: Commercially-acquired morphological genotypes reveal hidden allele variation among dog breeds, informing both trait ancestry and breed potential
Source: PLoS One. 2019 Oct 28;14(10):e0223995. doi: 10.1371/journal.pone.0223995 (PMC6816562; doi:10.1371/journal.pone.0223995)
Supplement: S2 Table — Allele frequencies for (a) coat color genes ASIP and MC1R, (b) the brown (TYRP1) and dominant black (CBD103) genes, (c) the white spotting (MITF), harlequin (PSMB71), and saddle tan (RALY) genes, (d) hair length (FGF5), hair curl (KRT71), and ear set, and (e) skull shape (BMP3) and natural taillessness (T). Breeds fixed for a single allele at any gene are indicated with bold text. (DOCX) [file pone.0223995.s004.docx]

**S2 Table (a).** Allele frequencies for coat color genes *ASIP* and *MC1R*. Breeds fixed for a single allele at any gene are indicated with bold text.

|  | ***ASIP*** | | | | | | ***MC1R*** | | | | | |
| --- | --- | --- | --- | --- | --- | --- | --- | --- | --- | --- | --- | --- |
| **Breed** | **n** | ***a^y^*** | ***a^w^*** | ***a^t^*** | ***a*** | ***a^yt^*** |  | **n** | ***E^M^*** | ***E^G^*** | ***E*** | ***e*** |
| ABUL | 37 | 0.96 | 0.00 | 0.04 | 0.00 | 0.00 |  | 39 | 0.76 | 0.00 | 0.22 | 0.03 |
| ACKR | 76 | 0.02 | 0.01 | 0.80 | 0.17 | 0.00 |  | 84 | 0.14 | 0.00 | 0.32 | 0.54 |
| AESK | 44 | 0.30 | 0.25 | 0.11 | 0.34 | 0.00 |  | 54 | 0.00 | 0.00 | 0.01 | 0.99 |
| AFFN | 21 | 0.38 | 0.00 | 0.21 | 0.40 | 0.00 |  | 40 | 0.50 | 0.00 | 0.46 | 0.04 |
| AFGH | 61 | 0.52 | 0.01 | 0.47 | 0.00 | 0.00 |  | 66 | 0.64 | 0.20 | 0.00 | 0.16 |
| AIRT | 56 | 0.00 | 0.00 | **1.00** | 0.00 | 0.00 |  | 57 | 0.00 | 0.00 | **1.00** | 0.00 |
| AKIT | 76 | 0.84 | 0.06 | 0.09 | 0.01 | 0.00 |  | 79 | 0.42 | 0.01 | 0.33 | 0.24 |
| AMAL | 68 | 0.00 | 0.88 | 0.10 | 0.02 | 0.00 |  | 69 | 0.00 | 0.00 | 0.93 | 0.07 |
| AMST | 270 | 0.81 | 0.00 | 0.19 | 0.00 | 0.00 |  | 292 | 0.66 | 0.00 | 0.30 | 0.04 |
| ANAT | 22 | 0.89 | 0.02 | 0.05 | 0.02 | 0.02 |  | 24 | 0.58 | 0.06 | 0.13 | 0.23 |
| AUCD | 39 | 0.19 | 0.00 | 0.81 | 0.00 | 0.00 |  | 43 | 0.38 | 0.00 | 0.60 | 0.01 |
| AUSS | 129 | 0.00 | 0.02 | 0.94 | 0.04 | 0.00 |  | 136 | 0.29 | 0.00 | 0.68 | 0.02 |
| AUST | 27 | 0.11 | 0.00 | 0.89 | 0.00 | 0.00 |  | 27 | 0.57 | 0.00 | 0.43 | 0.00 |
| AWSP | 32 | 0.00 | 0.00 | 0.38 | 0.63 | 0.00 |  | 34 | 0.00 | 0.00 | **1.00** | 0.00 |
| AZWK | 28 | 0.96 | 0.04 | 0.00 | 0.00 | 0.00 |  | 30 | 0.30 | 0.00 | 0.62 | 0.08 |
| BARB | 19 | 0.00 | 0.00 | 0.97 | 0.03 | 0.00 |  | 19 | 0.39 | 0.00 | 0.58 | 0.03 |
| BASS combined | 71 | 0.08 | 0.00 | 0.92 | 0.00 | 0.00 |  | 75 | 0.19 | 0.00 | 0.35 | 0.46 |
| BASS (UK) | 11 | 0.00 | 0.00 | **1.00** | 0.00 | 0.00 |  | 14 | 0.11 | 0.00 | 0.18 | 0.71 |
| BASS (US) | 60 | 0.09 | 0.00 | 0.91 | 0.00 | 0.00 |  | 61 | 0.21 | 0.00 | 0.39 | 0.40 |
| BBLS | 28 | 0.00 | 0.25 | 0.59 | 0.16 | 0.00 |  | 33 | 0.02 | 0.00 | 0.00 | 0.98 |
| BEAG combined | 109 | 0.01 | 0.00 | 0.99 | 0.00 | 0.00 |  | 111 | 0.04 | 0.00 | 0.68 | 0.28 |
| BEAG (UK) | 15 | 0.00 | 0.00 | **1.00** | 0.00 | 0.00 |  | 16 | 0.06 | 0.00 | 0.53 | 0.41 |
| BEAG (US Field) | 54 | 0.02 | 0.00 | 0.98 | 0.00 | 0.00 |  | 52 | 0.06 | 0.00 | 0.77 | 0.17 |
| BEAG (US Show) | 40 | 0.00 | 0.00 | **1.00** | 0.00 | 0.00 |  | 43 | 0.00 | 0.00 | 0.64 | 0.36 |
| BEAU | 25 | 0.00 | 0.00 | **1.00** | 0.00 | 0.00 |  | 28 | 0.14 | 0.00 | 0.86 | 0.00 |
| BEDT | 55 | 0.00 | 0.00 | **1.00** | 0.00 | 0.00 |  | 60 | 0.48 | 0.00 | 0.51 | 0.01 |
| BELS | 26 | 0.52 | 0.00 | 0.12 | 0.37 | 0.00 |  | 46 | 0.68 | 0.00 | 0.32 | 0.00 |
| BERD | 55 | 0.00 | 0.00 | 0.99 | 0.01 | 0.00 |  | 59 | 0.97 | 0.00 | 0.03 | 0.00 |
| BERG | 3 | 0.00 | 0.17 | 0.67 | 0.17 | 0.00 |  | 9 | 0.06 | 0.00 | 0.94 | 0.00 |
| BICH | 67 | 0.66 | 0.11 | 0.22 | 0.01 | 0.00 |  | 78 | 0.00 | 0.00 | 0.00 | **1.00** |
| BIEW | 29 | 0.02 | 0.00 | 0.98 | 0.00 | 0.00 |  | 29 | 0.26 | 0.00 | 0.71 | 0.03 |
| BLAB | 28 | 0.75 | 0.07 | 0.16 | 0.02 | 0.00 |  | 29 | 0.60 | 0.00 | 0.33 | 0.07 |
| BLDH | 43 | 0.13 | 0.00 | 0.87 | 0.00 | 0.00 |  | 43 | 0.71 | 0.00 | 0.29 | 0.00 |
| BMAL | 46 | 0.92 | 0.05 | 0.02 | 0.00 | 0.00 |  | 48 | **1.00** | 0.00 | 0.00 | 0.00 |
| BMD | 17 | 0.00 | 0.00 | **1.00** | 0.00 | 0.00 |  | 17 | 0.00 | 0.00 | **1.00** | 0.00 |
| BOER | 23 | **1.00** | 0.00 | 0.00 | 0.00 | 0.00 |  | 21 | 0.83 | 0.00 | 0.14 | 0.02 |
| BOLO | 16 | 0.34 | 0.09 | 0.38 | 0.19 | 0.00 |  | 19 | 0.00 | 0.00 | 0.00 | **1.00** |
| BORD | 90 | 0.01 | 0.21 | 0.78 | 0.00 | 0.00 |  | 96 | 0.41 | 0.00 | 0.54 | 0.06 |
| BORT | 83 | 0.55 | 0.00 | 0.45 | 0.00 | 0.00 |  | 91 | **1.00** | 0.00 | 0.00 | 0.00 |
| BORZ | 70 | 0.50 | 0.00 | 0.50 | 0.00 | 0.00 |  | 71 | 0.15 | 0.56 | 0.24 | 0.05 |
| BOST | 67 | 0.95 | 0.00 | 0.02 | 0.00 | 0.03 |  | 71 | 0.75 | 0.00 | 0.24 | 0.01 |
| BOUV | 43 | 0.88 | 0.00 | 0.12 | 0.00 | 0.00 |  | 46 | 0.70 | 0.00 | 0.30 | 0.00 |
| BOX | 75 | 0.99 | 0.00 | 0.01 | 0.00 | 0.00 |  | 73 | **1.00** | 0.00 | 0.00 | 0.00 |
| BOYK | 9 | 0.06 | 0.00 | 0.94 | 0.00 | 0.00 |  | 10 | 0.20 | 0.00 | 0.80 | 0.00 |
| BPIC | 9 | 0.94 | 0.00 | 0.00 | 0.00 | 0.06 |  | 10 | 0.00 | 0.00 | **1.00** | 0.00 |
| BRAC | 13 | 0.69 | 0.04 | 0.27 | 0.00 | 0.00 |  | 13 | 0.00 | 0.00 | 0.19 | 0.81 |
| BRIA | 55 | 0.94 | 0.00 | 0.06 | 0.00 | 0.00 |  | 55 | 0.36 | 0.00 | 0.64 | 0.00 |
| BRIT | 51 | 0.98 | 0.00 | 0.02 | 0.00 | 0.00 |  | 53 | 0.04 | 0.00 | 0.11 | 0.85 |
| BRTR | 35 | 0.01 | 0.01 | 0.94 | 0.03 | 0.00 |  | 36 | 0.46 | 0.01 | 0.51 | 0.01 |
| BRUS | 44 | 0.73 | 0.00 | 0.26 | 0.00 | 0.01 |  | 46 | 0.91 | 0.00 | 0.08 | 0.01 |
| BULD | 72 | 0.97 | 0.00 | 0.03 | 0.01 | 0.00 |  | 76 | 0.76 | 0.00 | 0.24 | 0.00 |
| BULM combined | 58 | **1.00** | 0.00 | 0.00 | 0.00 | 0.00 |  | 59 | 0.95 | 0.00 | 0.05 | 0.00 |
| BULM (UK) | 14 | **1.00** | 0.00 | 0.00 | 0.00 | 0.00 |  | 15 | 0.87 | 0.00 | 0.13 | 0.00 |
| BULM (US) | 44 | **1.00** | 0.00 | 0.00 | 0.00 | 0.00 |  | 44 | 0.98 | 0.00 | 0.02 | 0.00 |
| BULT | 58 | 0.45 | 0.01 | 0.53 | 0.02 | 0.00 |  | 59 | **1.00** | 0.00 | 0.00 | 0.00 |
| CAAN | 15 | 0.80 | 0.00 | 0.20 | 0.00 | 0.00 |  | 18 | 0.14 | 0.08 | 0.42 | 0.36 |
| CAIR | 57 | 0.93 | 0.00 | 0.07 | 0.00 | 0.00 |  | 61 | 0.97 | 0.00 | 0.01 | 0.02 |
| CANE | 41 | 0.72 | 0.02 | 0.24 | 0.01 | 0.00 |  | 44 | 0.94 | 0.00 | 0.06 | 0.00 |
| CARD | 51 | 0.29 | 0.00 | 0.71 | 0.00 | 0.00 |  | 53 | 0.22 | 0.00 | 0.67 | 0.11 |
| CASD | 20 | 0.70 | 0.10 | 0.15 | 0.05 | 0.00 |  | 25 | 0.18 | 0.26 | 0.32 | 0.24 |
| CAUC | 34 | 0.47 | 0.47 | 0.04 | 0.01 | 0.00 |  | 35 | 0.63 | 0.03 | 0.20 | 0.14 |
| CCRT | 76 | 0.00 | 0.00 | 0.93 | 0.07 | 0.00 |  | 79 | 0.24 | 0.00 | 0.76 | 0.00 |
| CESK | 13 | 0.00 | 0.00 | **1.00** | 0.00 | 0.00 |  | 15 | **1.00** | 0.00 | 0.00 | 0.00 |
| CHIH | 53 | 0.39 | 0.00 | 0.60 | 0.01 | 0.00 |  | 53 | 0.04 | 0.00 | 0.52 | 0.44 |
| CHIN | 48 | 0.34 | 0.00 | 0.66 | 0.00 | 0.00 |  | 49 | 0.00 | 0.00 | 0.71 | 0.29 |
| CHOW | 49 | 0.99 | 0.00 | 0.01 | 0.00 | 0.00 |  | 56 | 0.00 | 0.00 | 0.78 | 0.22 |
| CIRN | 14 | 0.96 | 0.00 | 0.04 | 0.00 | 0.00 |  | 16 | 0.00 | 0.00 | 0.16 | 0.84 |
| CKCS | 115 | 0.00 | 0.01 | 0.99 | 0.00 | 0.00 |  | 131 | 0.00 | 0.00 | 0.20 | 0.80 |
| CLSP | 54 | 0.03 | 0.00 | 0.97 | 0.00 | 0.00 |  | 56 | 0.00 | 0.00 | 0.00 | **1.00** |
| COLL combined | 86 | 0.27 | 0.00 | 0.73 | 0.00 | 0.00 |  | 93 | 0.00 | 0.00 | **1.00** | 0.00 |
| COLL (UK) | 22 | 0.45 | 0.00 | 0.55 | 0.00 | 0.00 |  | 27 | 0.00 | 0.00 | **1.00** | 0.00 |
| COLL (US) | 64 | 0.20 | 0.00 | 0.80 | 0.00 | 0.00 |  | 66 | 0.00 | 0.00 | **1.00** | 0.00 |
| COOK | 35 | 0.74 | 0.16 | 0.10 | 0.00 | 0.00 |  | 36 | 0.19 | 0.00 | 0.39 | 0.42 |
| COTO | 73 | 0.66 | 0.11 | 0.22 | 0.01 | 0.00 |  | 83 | 0.06 | 0.00 | 0.13 | 0.81 |
| COYO (Eastern) | 25 | 0.00 | 0.80 | 0.20 | 0.00 | 0.00 |  | 26 | 0.00 | 0.02 | 0.98 | 0.00 |
| COYO (Western) | 18 | 0.00 | **1.00** | 0.00 | 0.00 | 0.00 |  | 11 | 0.00 | 0.00 | **1.00** | 0.00 |
| CPBR | 35 | 0.97 | 0.00 | 0.00 | 0.03 | 0.00 |  | 45 | 0.46 | 0.00 | 0.53 | 0.01 |
| CRES | 25 | 0.70 | 0.00 | 0.28 | 0.02 | 0.00 |  | 28 | 0.09 | 0.00 | 0.59 | 0.32 |
| DACH combined | 150 | 0.26 | 0.25 | 0.49 | 0.00 | 0.00 |  | 172 | 0.01 | 0.01 | 0.89 | 0.09 |
| DACH (Miniature Longhair) | 16 | 0.28 | 0.06 | 0.66 | 0.00 | 0.00 |  | 23 | 0.02 | 0.00 | 0.70 | 0.28 |
| DACH (Miniature Shorthair) | 43 | 0.23 | 0.02 | 0.74 | 0.00 | 0.00 |  | 48 | 0.01 | 0.03 | 0.83 | 0.13 |
| DACH (Miniature Wirehair) | 24 | 0.29 | 0.44 | 0.27 | 0.00 | 0.00 |  | 28 | 0.04 | 0.00 | 0.91 | 0.05 |
| DACH (Standard Longhair) | 22 | 0.57 | 0.00 | 0.43 | 0.00 | 0.00 |  | 23 | 0.00 | 0.00 | 0.93 | 0.07 |
| DACH (Standard Shorthair) | 14 | 0.18 | 0.00 | 0.82 | 0.00 | 0.00 |  | 19 | 0.00 | 0.00 | **1.00** | 0.00 |
| DACH (Standard Wirehair) | 31 | 0.08 | 0.82 | 0.10 | 0.00 | 0.00 |  | 31 | 0.00 | 0.00 | **1.00** | 0.00 |
| DALM combined | 44 | 0.00 | 0.11 | 0.86 | 0.02 | 0.00 |  | 46 | 0.55 | 0.00 | 0.40 | 0.04 |
| DALM (UK) | 10 | 0.00 | 0.15 | 0.80 | 0.05 | 0.00 |  | 12 | 0.67 | 0.00 | 0.29 | 0.04 |
| DALM (US) | 34 | 0.00 | 0.10 | 0.88 | 0.01 | 0.00 |  | 34 | 0.51 | 0.00 | 0.44 | 0.04 |
| DANE | 79 | 0.91 | 0.05 | 0.03 | 0.01 | 0.01 |  | 85 | 0.55 | 0.00 | 0.45 | 0.00 |
| DDBX | 28 | **1.00** | 0.00 | 0.00 | 0.00 | 0.00 |  | 33 | 0.85 | 0.00 | 0.15 | 0.00 |
| DDMT | 38 | 0.46 | 0.00 | 0.54 | 0.00 | 0.00 |  | 41 | 0.76 | 0.00 | 0.24 | 0.00 |
| DEER | 33 | 0.79 | 0.00 | 0.21 | 0.00 | 0.00 |  | 34 | **1.00** | 0.00 | 0.00 | 0.00 |
| DING | 12 | 0.75 | 0.00 | 0.17 | 0.00 | 0.08 |  | 12 | 0.00 | 0.00 | **1.00** | 0.00 |
| DOBP | 109 | 0.00 | 0.00 | **1.00** | 0.00 | 0.00 |  | 115 | 0.16 | 0.00 | 0.84 | 0.00 |
| DOGO | 9 | 0.61 | 0.00 | 0.06 | 0.00 | 0.33 |  | 14 | 0.89 | 0.00 | 0.00 | 0.11 |
| ECKR combined | 82 | 0.00 | 0.00 | 0.93 | 0.07 | 0.00 |  | 89 | 0.16 | 0.00 | 0.39 | 0.46 |
| ECKR (Field) | 17 | 0.00 | 0.00 | **1.00** | 0.00 | 0.00 |  | 17 | 0.00 | 0.00 | 0.59 | 0.41 |
| ECKR (Show) | 65 | 0.00 | 0.00 | 0.92 | 0.08 | 0.00 |  | 72 | 0.19 | 0.00 | 0.34 | 0.47 |
| ESET | 55 | 0.00 | 0.00 | **1.00** | 0.00 | 0.00 |  | 62 | 0.25 | 0.00 | 0.06 | 0.69 |
| ESSP combined | 93 | 0.01 | 0.11 | 0.83 | 0.04 | 0.00 |  | 98 | 0.28 | 0.00 | 0.72 | 0.00 |
| ESSP (UK Field) | 31 | 0.03 | 0.03 | 0.94 | 0.00 | 0.00 |  | 32 | 0.02 | 0.00 | 0.98 | 0.00 |
| ESSP (UK Show) | 25 | 0.00 | 0.38 | 0.48 | 0.14 | 0.00 |  | 29 | 0.33 | 0.00 | 0.67 | 0.00 |
| ESSP (US Show) | 37 | 0.00 | 0.00 | 0.99 | 0.01 | 0.00 |  | 37 | 0.47 | 0.00 | 0.53 | 0.00 |
| EURA | 26 | 0.44 | 0.13 | 0.23 | 0.19 | 0.00 |  | 26 | 0.40 | 0.00 | 0.52 | 0.08 |
| FBUL | 61 | 0.93 | 0.00 | 0.06 | 0.01 | 0.00 |  | 67 | 0.45 | 0.00 | 0.13 | 0.43 |
| FCR | 70 | 0.00 | 0.00 | 0.69 | 0.31 | 0.00 |  | 80 | 0.03 | 0.00 | 0.89 | 0.08 |
| FIEL | 38 | 0.00 | 0.00 | 0.93 | 0.07 | 0.00 |  | 42 | 0.18 | 0.00 | 0.82 | 0.00 |
| FLAP | 21 | 0.17 | 0.00 | 0.64 | 0.19 | 0.00 |  | 26 | 0.00 | 0.00 | 0.69 | 0.31 |
| FOXH | 29 | 0.05 | 0.00 | 0.95 | 0.00 | 0.00 |  | 32 | 0.19 | 0.02 | 0.61 | 0.19 |
| GALG | 25 | 0.98 | 0.00 | 0.00 | 0.00 | 0.02 |  | 25 | 0.00 | 0.00 | 0.80 | 0.20 |
| GLEN | 14 | **1.00** | 0.00 | 0.00 | 0.00 | 0.00 |  | 14 | 0.89 | 0.00 | 0.11 | 0.00 |
| GOLD combined | 169 | 0.00 | 0.00 | 0.79 | 0.20 | 0.00 |  | 180 | 0.00 | 0.00 | 0.00 | **1.00** |
| GOLD (UK) | 32 | 0.00 | 0.00 | 0.89 | 0.11 | 0.00 |  | 39 | 0.00 | 0.00 | 0.00 | **1.00** |
| GOLD (US) | 137 | 0.00 | 0.00 | 0.77 | 0.22 | 0.00 |  | 141 | 0.00 | 0.00 | 0.00 | **1.00** |
| GORD | 45 | 0.00 | 0.00 | **1.00** | 0.00 | 0.00 |  | 45 | 0.00 | 0.00 | 0.91 | 0.09 |
| GPIN | 24 | 0.50 | 0.00 | 0.50 | 0.00 | 0.00 |  | 25 | 0.00 | 0.00 | 0.98 | 0.02 |
| GPYR | 51 | 0.77 | 0.08 | 0.04 | 0.01 | 0.10 |  | 50 | 0.00 | 0.00 | 0.53 | 0.47 |
| GREY combined | 99 | 0.96 | 0.01 | 0.03 | 0.00 | 0.00 |  | 106 | 0.11 | 0.00 | 0.88 | 0.00 |
| GREY (Racing) | 76 | 0.95 | 0.01 | 0.03 | 0.00 | 0.00 |  | 81 | 0.14 | 0.01 | 0.86 | 0.00 |
| GREY (Show) | 23 | **1.00** | 0.00 | 0.00 | 0.00 | 0.00 |  | 25 | 0.02 | 0.00 | 0.96 | 0.02 |
| GSD | 144 | 0.01 | 0.14 | 0.66 | 0.19 | 0.00 |  | 156 | 0.76 | 0.00 | 0.18 | 0.06 |
| GSHP combined | 62 | 0.00 | 0.00 | 0.77 | 0.23 | 0.00 |  | 73 | 0.14 | 0.00 | 0.86 | 0.00 |
| GSHP (UK) | 26 | 0.00 | 0.00 | 0.73 | 0.27 | 0.00 |  | 37 | 0.27 | 0.00 | 0.73 | 0.00 |
| GSHP (US) | 36 | 0.00 | 0.00 | 0.81 | 0.19 | 0.00 |  | 36 | 0.01 | 0.00 | 0.99 | 0.00 |
| GSNZ | 43 | 0.03 | 0.22 | 0.58 | 0.16 | 0.00 |  | 44 | 0.25 | 0.00 | 0.75 | 0.00 |
| GSPZ combined | 29 | 0.40 | 0.29 | 0.03 | 0.28 | 0.00 |  | 44 | 0.27 | 0.00 | 0.17 | 0.56 |
| GSPZ (UK) | 24 | 0.48 | 0.25 | 0.04 | 0.23 | 0.00 |  | 36 | 0.26 | 0.00 | 0.19 | 0.54 |
| GSPZ (US) | 5 | 0.00 | 0.50 | 0.00 | 0.50 | 0.00 |  | 8 | 0.31 | 0.00 | 0.06 | 0.63 |
| GWHP | 31 | 0.00 | 0.26 | 0.66 | 0.08 | 0.00 |  | 32 | 0.03 | 0.00 | 0.95 | 0.02 |
| HARR | 32 | 0.00 | 0.00 | **1.00** | 0.00 | 0.00 |  | 31 | 0.39 | 0.00 | 0.31 | 0.31 |
| HAVA | 59 | 0.23 | 0.00 | 0.77 | 0.00 | 0.00 |  | 61 | 0.02 | 0.00 | 0.61 | 0.37 |
| HUSK | 107 | 0.01 | 0.86 | 0.13 | 0.00 | 0.00 |  | 112 | 0.00 | 0.00 | 0.92 | 0.08 |
| IBIZ | 31 | 0.81 | 0.02 | 0.18 | 0.00 | 0.00 |  | 35 | 0.00 | 0.00 | 0.00 | **1.00** |
| ICES | 27 | 0.57 | 0.22 | 0.15 | 0.06 | 0.00 |  | 29 | 0.02 | 0.00 | 0.86 | 0.12 |
| INCA | 19 | 0.32 | 0.13 | 0.53 | 0.03 | 0.00 |  | 21 | 0.36 | 0.00 | 0.43 | 0.21 |
| IRIT | 33 | 0.95 | 0.00 | 0.03 | 0.00 | 0.02 |  | 33 | 0.35 | 0.00 | 0.65 | 0.00 |
| ISET combined | 68 | 0.15 | 0.08 | 0.73 | 0.04 | 0.00 |  | 69 | 0.00 | 0.00 | 0.00 | **1.00** |
| ISET (UK) | 22 | 0.36 | 0.11 | 0.41 | 0.11 | 0.00 |  | 20 | 0.00 | 0.00 | 0.00 | **1.00** |
| ISET (US) | 46 | 0.05 | 0.07 | 0.88 | 0.00 | 0.00 |  | 49 | 0.00 | 0.00 | 0.00 | **1.00** |
| ITGY | 67 | 0.95 | 0.01 | 0.04 | 0.00 | 0.00 |  | 69 | 0.76 | 0.00 | 0.14 | 0.09 |
| IWOF | 45 | 0.91 | 0.02 | 0.00 | 0.07 | 0.00 |  | 65 | 0.57 | 0.00 | 0.43 | 0.00 |
| IWSP | 32 | 0.00 | 0.00 | 0.75 | 0.25 | 0.00 |  | 32 | 0.52 | 0.00 | 0.48 | 0.00 |
| JIND | 9 | 0.67 | 0.28 | 0.06 | 0.00 | 0.00 |  | 10 | 0.00 | 0.00 | 0.75 | 0.25 |
| JSPZ | 11 | 0.64 | 0.00 | 0.05 | 0.32 | 0.00 |  | 19 | 0.00 | 0.00 | 0.00 | **1.00** |
| KEES combined | 52 | 0.00 | 0.98 | 0.02 | 0.00 | 0.00 |  | 53 | 0.96 | 0.00 | 0.03 | 0.01 |
| KEES (UK) | 17 | 0.00 | 0.97 | 0.03 | 0.00 | 0.00 |  | 18 | **1.00** | 0.00 | 0.00 | 0.00 |
| KEES (US) | 35 | 0.00 | 0.99 | 0.01 | 0.00 | 0.00 |  | 35 | 0.94 | 0.00 | 0.04 | 0.01 |
| KELP | 54 | 0.00 | 0.02 | 0.98 | 0.00 | 0.00 |  | 60 | 0.29 | 0.00 | 0.66 | 0.05 |
| KERY | 40 | 0.03 | 0.01 | 0.96 | 0.00 | 0.00 |  | 41 | 0.28 | 0.00 | 0.72 | 0.00 |
| KKLG | 39 | 0.40 | 0.08 | 0.44 | 0.09 | 0.00 |  | 41 | 0.02 | 0.15 | 0.48 | 0.35 |
| KOMO | 25 | 0.48 | 0.20 | 0.32 | 0.00 | 0.00 |  | 26 | 0.00 | 0.00 | 0.00 | **1.00** |
| KOOL | 34 | 0.01 | 0.01 | 0.97 | 0.00 | 0.00 |  | 41 | 0.20 | 0.00 | 0.67 | 0.13 |
| KUVZ | 13 | 0.38 | 0.12 | 0.35 | 0.15 | 0.00 |  | 14 | 0.00 | 0.00 | 0.14 | 0.86 |
| LAB combined | 355 | 0.01 | 0.01 | 0.88 | 0.10 | 0.00 |  | 371 | 0.07 | 0.00 | 0.33 | 0.60 |
| LAB (UK Show) | 91 | 0.00 | 0.01 | 0.92 | 0.07 | 0.00 |  | 104 | 0.03 | 0.00 | 0.51 | 0.46 |
| LAB (US Field) | 174 | 0.02 | 0.01 | 0.86 | 0.11 | 0.00 |  | 175 | 0.13 | 0.00 | 0.26 | 0.61 |
| LAB (US Show) | 90 | 0.00 | 0.01 | 0.87 | 0.13 | 0.00 |  | 92 | 0.01 | 0.00 | 0.24 | 0.75 |
| LAGO | 116 | 0.06 | 0.01 | 0.78 | 0.11 | 0.03 |  | 135 | 0.03 | 0.00 | 0.43 | 0.54 |
| LAKE | 42 | 0.00 | 0.00 | **1.00** | 0.00 | 0.00 |  | 44 | 0.05 | 0.00 | 0.40 | 0.56 |
| LANC | 8 | 0.00 | 0.00 | **1.00** | 0.00 | 0.00 |  | 11 | 0.09 | 0.00 | 0.91 | 0.00 |
| LEOP | 19 | 0.05 | 0.00 | 0.87 | 0.08 | 0.00 |  | 25 | 0.46 | 0.00 | 0.52 | 0.02 |
| LHAS combined | 48 | 0.75 | 0.00 | 0.25 | 0.00 | 0.00 |  | 59 | 0.20 | 0.00 | 0.66 | 0.14 |
| LHAS (UK) | 15 | 0.77 | 0.00 | 0.23 | 0.00 | 0.00 |  | 18 | 0.42 | 0.00 | 0.56 | 0.03 |
| LHAS (US) | 33 | 0.74 | 0.00 | 0.26 | 0.00 | 0.00 |  | 41 | 0.11 | 0.00 | 0.71 | 0.18 |
| LMUN | 18 | 0.03 | 0.00 | 0.94 | 0.03 | 0.00 |  | 21 | 0.40 | 0.02 | 0.57 | 0.00 |
| LOWC | 32 | 0.17 | 0.05 | 0.78 | 0.00 | 0.00 |  | 36 | 0.42 | 0.00 | 0.28 | 0.31 |
| MALT combined | 92 | 0.34 | 0.03 | 0.63 | 0.01 | 0.00 |  | 88 | 0.00 | 0.01 | 0.00 | 0.99 |
| MALT (UK) | 18 | 0.36 | 0.00 | 0.64 | 0.00 | 0.00 |  | 18 | 0.00 | 0.00 | 0.00 | **1.00** |
| MALT (US) | 74 | 0.34 | 0.03 | 0.62 | 0.01 | 0.00 |  | 70 | 0.00 | 0.01 | 0.00 | 0.99 |
| MANT combined | 32 | 0.00 | 0.00 | **1.00** | 0.00 | 0.00 |  | 35 | 0.00 | 0.01 | 0.99 | 0.00 |
| MANT (UK) | 16 | 0.00 | 0.00 | **1.00** | 0.00 | 0.00 |  | 19 | 0.00 | 0.03 | 0.97 | 0.00 |
| MANT (US) | 16 | 0.00 | 0.00 | **1.00** | 0.00 | 0.00 |  | 16 | 0.00 | 0.00 | **1.00** | 0.00 |
| MARM | 18 | 0.28 | 0.06 | 0.50 | 0.06 | 0.11 |  | 20 | 0.00 | 0.00 | 0.00 | **1.00** |
| MAST | 56 | **1.00** | 0.00 | 0.00 | 0.00 | 0.00 |  | 59 | 0.98 | 0.00 | 0.02 | 0.00 |
| MBLT | 26 | 0.60 | 0.00 | 0.40 | 0.00 | 0.00 |  | 27 | **1.00** | 0.00 | 0.00 | 0.00 |
| MCNB | 26 | 0.10 | 0.10 | 0.62 | 0.19 | 0.00 |  | 27 | 0.22 | 0.00 | 0.78 | 0.00 |
| MGAG | 20 | 0.88 | 0.00 | 0.13 | 0.00 | 0.00 |  | 23 | 0.07 | 0.00 | 0.91 | 0.02 |
| MIKI | 19 | 0.47 | 0.00 | 0.50 | 0.03 | 0.00 |  | 22 | 0.09 | 0.02 | 0.61 | 0.27 |
| MPIN | 51 | 0.21 | 0.00 | 0.79 | 0.00 | 0.00 |  | 52 | 0.14 | 0.00 | 0.42 | 0.43 |
| MSNZ | 82 | 0.00 | 0.63 | 0.37 | 0.00 | 0.00 |  | 83 | 0.29 | 0.00 | 0.57 | 0.14 |
| MUDI | 32 | 0.02 | 0.06 | 0.02 | 0.91 | 0.00 |  | 39 | 0.13 | 0.00 | 0.53 | 0.35 |
| NBUH | 11 | 0.91 | 0.00 | 0.09 | 0.00 | 0.00 |  | 11 | 0.09 | 0.05 | 0.77 | 0.09 |
| NEAP | 25 | 0.70 | 0.00 | 0.16 | 0.14 | 0.00 |  | 39 | **1.00** | 0.00 | 0.00 | 0.00 |
| NELK | 90 | 0.00 | **1.00** | 0.00 | 0.00 | 0.00 |  | 90 | **1.00** | 0.00 | 0.00 | 0.00 |
| NEWF | 55 | 0.00 | 0.01 | 0.45 | 0.55 | 0.00 |  | 66 | 0.20 | 0.00 | 0.80 | 0.01 |
| NLUN | 9 | **1.00** | 0.00 | 0.00 | 0.00 | 0.00 |  | 17 | 0.00 | 0.03 | 0.97 | 0.00 |
| NORF | 44 | 0.63 | 0.00 | 0.38 | 0.00 | 0.00 |  | 51 | 0.69 | 0.00 | 0.30 | 0.01 |
| NOWT | 60 | 0.39 | 0.00 | 0.61 | 0.00 | 0.00 |  | 60 | 0.43 | 0.00 | 0.43 | 0.13 |
| NSDT | 35 | 0.96 | 0.00 | 0.04 | 0.00 | 0.00 |  | 37 | 0.00 | 0.00 | 0.11 | 0.89 |
| OES | 59 | 0.00 | 0.28 | 0.72 | 0.00 | 0.00 |  | 64 | 0.43 | 0.00 | 0.57 | 0.00 |
| OTTR | 30 | 0.00 | 0.00 | 0.98 | 0.02 | 0.00 |  | 34 | 0.44 | 0.00 | 0.56 | 0.00 |
| PAP | 71 | 0.42 | 0.00 | 0.49 | 0.09 | 0.00 |  | 78 | 0.40 | 0.00 | 0.54 | 0.06 |
| PBGV | 46 | 0.07 | 0.54 | 0.39 | 0.00 | 0.00 |  | 47 | 0.00 | 0.00 | 0.76 | 0.24 |
| PDCN | 19 | 0.82 | 0.05 | 0.13 | 0.00 | 0.00 |  | 20 | 0.03 | 0.00 | 0.00 | 0.98 |
| PEKE | 50 | 0.96 | 0.00 | 0.04 | 0.00 | 0.00 |  | 56 | 0.46 | 0.00 | 0.29 | 0.26 |
| PEMB combined | 71 | 0.48 | 0.00 | 0.51 | 0.01 | 0.00 |  | 75 | 0.03 | 0.01 | 0.95 | 0.01 |
| PEMB (UK) | 24 | 0.48 | 0.00 | 0.50 | 0.02 | 0.00 |  | 28 | 0.00 | 0.02 | 0.98 | 0.00 |
| PEMB (US) | 47 | 0.48 | 0.00 | 0.52 | 0.00 | 0.00 |  | 47 | 0.05 | 0.00 | 0.94 | 0.01 |
| PHAR | 21 | **1.00** | 0.00 | 0.00 | 0.00 | 0.00 |  | 21 | 0.00 | 0.00 | 0.00 | **1.00** |
| PLOT | 23 | 0.46 | 0.02 | 0.52 | 0.00 | 0.00 |  | 25 | 0.14 | 0.00 | 0.64 | 0.22 |
| PNTR | 73 | 0.00 | 0.03 | 0.88 | 0.10 | 0.00 |  | 76 | 0.00 | 0.00 | 0.47 | 0.53 |
| POLG | 19 | 0.11 | 0.16 | 0.74 | 0.00 | 0.00 |  | 21 | 0.48 | 0.43 | 0.07 | 0.02 |
| Poodle combined | 246 | 0.13 | 0.05 | 0.70 | 0.12 | 0.00 |  | 273 | 0.12 | 0.00 | 0.25 | 0.62 |
| MePOO | 13 | 0.08 | 0.04 | 0.73 | 0.15 | 0.00 |  | 17 | 0.26 | 0.00 | 0.29 | 0.44 |
| MPOO | 125 | 0.10 | 0.10 | 0.74 | 0.07 | 0.00 |  | 126 | 0.04 | 0.00 | 0.11 | 0.85 |
| SPOO | 73 | 0.27 | 0.00 | 0.49 | 0.24 | 0.00 |  | 83 | 0.11 | 0.00 | 0.28 | 0.61 |
| TPOO | 35 | 0.01 | 0.03 | 0.96 | 0.00 | 0.00 |  | 47 | 0.34 | 0.01 | 0.55 | 0.10 |
| POM combined | 77 | 0.76 | 0.02 | 0.21 | 0.01 | 0.00 |  | 89 | 0.01 | 0.01 | 0.54 | 0.44 |
| POM (UK) | 12 | 0.88 | 0.04 | 0.08 | 0.00 | 0.00 |  | 14 | 0.07 | 0.00 | 0.68 | 0.25 |
| POM (US) | 65 | 0.74 | 0.02 | 0.24 | 0.01 | 0.00 |  | 75 | 0.00 | 0.01 | 0.52 | 0.47 |
| PONS | 19 | 0.26 | 0.00 | 0.74 | 0.00 | 0.00 |  | 21 | 0.45 | 0.00 | 0.50 | 0.05 |
| PRES | 23 | 0.98 | 0.02 | 0.00 | 0.00 | 0.00 |  | 28 | 0.84 | 0.00 | 0.16 | 0.00 |
| PRUS | 110 | 0.24 | 0.00 | 0.76 | 0.00 | 0.00 |  | 123 | 0.59 | 0.00 | 0.38 | 0.03 |
| PTWD | 41 | 0.23 | 0.00 | 0.38 | 0.39 | 0.00 |  | 50 | 0.54 | 0.00 | 0.46 | 0.00 |
| PUG | 54 | **1.00** | 0.00 | 0.00 | 0.00 | 0.00 |  | 59 | **1.00** | 0.00 | 0.00 | 0.00 |
| PULI | 25 | 0.14 | 0.00 | 0.36 | 0.50 | 0.00 |  | 38 | 0.11 | 0.03 | 0.68 | 0.18 |
| PUMI | 42 | 0.11 | 0.06 | 0.52 | 0.31 | 0.00 |  | 51 | 0.09 | 0.00 | 0.55 | 0.36 |
| PYRS | 13 | 0.88 | 0.00 | 0.08 | 0.04 | 0.00 |  | 13 | 0.88 | 0.00 | 0.08 | 0.04 |
| REDB | 7 | 0.86 | 0.00 | 0.14 | 0.00 | 0.00 |  | 9 | 0.28 | 0.00 | 0.72 | 0.00 |
| RHOD | 57 | 0.98 | 0.00 | 0.02 | 0.00 | 0.00 |  | 62 | 0.61 | 0.00 | 0.39 | 0.00 |
| ROTT | 110 | 0.00 | 0.00 | 0.99 | 0.00 | 0.00 |  | 117 | 0.06 | 0.00 | 0.94 | 0.00 |
| RUSS | 83 | 0.44 | 0.00 | 0.56 | 0.00 | 0.00 |  | 89 | 0.46 | 0.00 | 0.43 | 0.11 |
| RWST | 9 | 0.22 | 0.06 | 0.72 | 0.00 | 0.00 |  | 8 | 0.00 | 0.00 | 0.00 | **1.00** |
| SALU | 109 | 0.31 | 0.01 | 0.68 | 0.00 | 0.00 |  | 116 | 0.01 | 0.31 | 0.31 | 0.36 |
| SAMO | 18 | 0.00 | 0.00 | 0.08 | 0.92 | 0.00 |  | 18 | 0.00 | 0.00 | 0.00 | **1.00** |
| SCOT | 39 | 0.14 | 0.46 | 0.13 | 0.27 | 0.00 |  | 70 | 0.30 | 0.00 | 0.50 | 0.20 |
| SCWT combined | 49 | 0.97 | 0.00 | 0.03 | 0.00 | 0.00 |  | 51 | 0.85 | 0.00 | 0.00 | 0.15 |
| SCWT (UK) | 3 | 0.83 | 0.00 | 0.17 | 0.00 | 0.00 |  | 5 | **1.00** | 0.00 | 0.00 | 0.00 |
| SCWT (US) | 46 | 0.98 | 0.00 | 0.02 | 0.00 | 0.00 |  | 46 | 0.84 | 0.00 | 0.00 | 0.16 |
| SEAL | 25 | 0.02 | 0.00 | 0.98 | 0.00 | 0.00 |  | 29 | 0.91 | 0.00 | 0.09 | 0.00 |
| SFOX | 46 | 0.12 | 0.00 | 0.88 | 0.00 | 0.00 |  | 47 | **1.00** | 0.00 | 0.00 | 0.00 |
| SHAR | 54 | 0.81 | 0.17 | 0.02 | 0.00 | 0.00 |  | 62 | 0.44 | 0.00 | 0.31 | 0.25 |
| SHIB | 62 | 0.60 | 0.00 | 0.40 | 0.00 | 0.00 |  | 65 | 0.00 | 0.00 | 0.75 | 0.25 |
| SHIH | 85 | 0.92 | 0.00 | 0.08 | 0.00 | 0.00 |  | 87 | 0.66 | 0.00 | 0.26 | 0.09 |
| SILK | 42 | 0.00 | 0.00 | **1.00** | 0.00 | 0.00 |  | 45 | 0.54 | 0.00 | 0.46 | 0.00 |
| SKIP combined | 28 | 0.00 | 0.02 | 0.38 | 0.61 | 0.00 |  | 48 | 0.23 | 0.00 | 0.72 | 0.05 |
| SKIP (UK) | 2 | 0.00 | 0.00 | 0.50 | 0.50 | 0.00 |  | 5 | 0.00 | 0.00 | 0.50 | 0.50 |
| SKIP (US) | 26 | 0.00 | 0.02 | 0.37 | 0.62 | 0.00 |  | 43 | 0.26 | 0.00 | 0.74 | 0.00 |
| SKYE | 10 | 0.95 | 0.00 | 0.05 | 0.00 | 0.00 |  | 8 | **1.00** | 0.00 | 0.00 | 0.00 |
| SLOU | 19 | 0.55 | 0.08 | 0.37 | 0.00 | 0.00 |  | 21 | 0.76 | 0.00 | 0.21 | 0.02 |
| SMUN | 2 | 0.00 | 0.00 | 0.50 | 0.50 | 0.00 |  | 8 | 0.25 | 0.00 | 0.75 | 0.00 |
| SPIN | 51 | 0.04 | 0.03 | 0.88 | 0.05 | 0.00 |  | 54 | 0.06 | 0.00 | 0.04 | 0.90 |
| SPWD | 12 | 0.00 | 0.04 | 0.96 | 0.00 | 0.00 |  | 14 | 0.21 | 0.00 | 0.43 | 0.36 |
| SSHP combined | 72 | 0.42 | 0.00 | 0.44 | 0.13 | 0.00 |  | 87 | 0.00 | 0.00 | **1.00** | 0.00 |
| SSHP (UK) | 25 | 0.36 | 0.00 | 0.64 | 0.00 | 0.00 |  | 32 | 0.00 | 0.00 | **1.00** | 0.00 |
| SSHP (US) | 47 | 0.46 | 0.00 | 0.34 | 0.20 | 0.00 |  | 55 | 0.00 | 0.00 | **1.00** | 0.00 |
| SSNZ | 32 | 0.00 | 0.98 | 0.02 | 0.00 | 0.00 |  | 32 | 0.59 | 0.00 | 0.41 | 0.00 |
| STAF | 67 | 0.67 | 0.00 | 0.32 | 0.01 | 0.00 |  | 74 | 0.48 | 0.00 | 0.51 | 0.01 |
| SUSX | 16 | 0.00 | 0.19 | 0.63 | 0.19 | 0.00 |  | 17 | 0.00 | 0.00 | **1.00** | 0.00 |
| SVAL | 38 | 0.00 | **1.00** | 0.00 | 0.00 | 0.00 |  | 38 | 0.08 | 0.00 | 0.88 | 0.04 |
| TAIG | 19 | 0.71 | 0.05 | 0.24 | 0.00 | 0.00 |  | 22 | 0.02 | 0.11 | 0.32 | 0.55 |
| TENT | 8 | 0.06 | 0.00 | 0.94 | 0.00 | 0.00 |  | 10 | 0.50 | 0.00 | 0.50 | 0.00 |
| TERV | 35 | **1.00** | 0.00 | 0.00 | 0.00 | 0.00 |  | 39 | **1.00** | 0.00 | 0.00 | 0.00 |
| TIBM combined | 83 | 0.11 | 0.01 | 0.72 | 0.13 | 0.04 |  | 93 | 0.10 | 0.01 | 0.84 | 0.04 |
| TIBM (China) | 53 | 0.15 | 0.01 | 0.78 | 0.05 | 0.01 |  | 57 | 0.13 | 0.00 | 0.80 | 0.07 |
| TIBM (US) | 30 | 0.05 | 0.00 | 0.60 | 0.27 | 0.08 |  | 36 | 0.06 | 0.03 | 0.92 | 0.00 |
| TIBS | 46 | 0.74 | 0.01 | 0.24 | 0.00 | 0.01 |  | 49 | 0.04 | 0.00 | 0.84 | 0.12 |
| TIBT | 51 | 0.47 | 0.01 | 0.40 | 0.12 | 0.00 |  | 67 | 0.37 | 0.00 | 0.45 | 0.19 |
| TMNT | 36 | 0.00 | 0.01 | 0.99 | 0.00 | 0.00 |  | 39 | 0.00 | 0.00 | **1.00** | 0.00 |
| TREE | 42 | 0.02 | 0.00 | 0.96 | 0.01 | 0.00 |  | 41 | 0.07 | 0.01 | 0.87 | 0.05 |
| VIZS combined | 83 | 0.11 | 0.01 | 0.88 | 0.01 | 0.00 |  | 88 | 0.00 | 0.00 | 0.00 | **1.00** |
| VIZS (UK) | 21 | 0.05 | 0.02 | 0.90 | 0.02 | 0.00 |  | 25 | 0.00 | 0.00 | 0.00 | **1.00** |
| VIZS (US) | 62 | 0.13 | 0.00 | 0.87 | 0.00 | 0.00 |  | 63 | 0.00 | 0.00 | 0.00 | **1.00** |
| VPIN | 19 | 0.74 | 0.21 | 0.00 | 0.05 | 0.00 |  | 19 | 0.00 | 0.00 | 0.00 | **1.00** |
| WEIM combined | 56 | 0.00 | 0.00 | 0.83 | 0.17 | 0.00 |  | 63 | 0.08 | 0.00 | 0.92 | 0.00 |
| WEIM (UK) | 16 | 0.00 | 0.00 | 0.72 | 0.28 | 0.00 |  | 20 | 0.05 | 0.00 | 0.95 | 0.00 |
| WEIM (US) | 40 | 0.00 | 0.00 | 0.88 | 0.13 | 0.00 |  | 43 | 0.09 | 0.00 | 0.91 | 0.00 |
| WELT combined | 57 | 0.00 | 0.00 | **1.00** | 0.00 | 0.00 |  | 66 | 0.06 | 0.00 | 0.94 | 0.00 |
| WELT (UK) | 16 | 0.00 | 0.00 | **1.00** | 0.00 | 0.00 |  | 17 | 0.00 | 0.00 | **1.00** | 0.00 |
| WELT (US) | 41 | 0.00 | 0.00 | **1.00** | 0.00 | 0.00 |  | 49 | 0.08 | 0.00 | 0.92 | 0.00 |
| WFOX | 25 | 0.00 | 0.00 | **1.00** | 0.00 | 0.00 |  | 31 | 0.05 | 0.00 | 0.95 | 0.00 |
| WHIP combined | 68 | 0.99 | 0.00 | 0.00 | 0.00 | 0.01 |  | 76 | 0.38 | 0.00 | 0.53 | 0.09 |
| WHIP (Racing) | 9 | 0.94 | 0.00 | 0.00 | 0.00 | 0.06 |  | 9 | 0.67 | 0.00 | 0.33 | 0.00 |
| WHIP (UK Show) | 29 | **1.00** | 0.00 | 0.00 | 0.00 | 0.00 |  | 37 | 0.50 | 0.00 | 0.45 | 0.05 |
| WHIP (US Show) | 30 | **1.00** | 0.00 | 0.00 | 0.00 | 0.00 |  | 30 | 0.15 | 0.00 | 0.70 | 0.15 |
| WHPG | 19 | 0.08 | 0.03 | 0.66 | 0.24 | 0.00 |  | 20 | 0.00 | 0.00 | **1.00** | 0.00 |
| WHWT | 69 | 0.99 | 0.01 | 0.00 | 0.00 | 0.00 |  | 69 | 0.00 | 0.00 | 0.00 | **1.00** |
| WOLF | 11 | 0.00 | 0.86 | 0.14 | 0.00 | 0.00 |  | 10 | 0.00 | 0.00 | **1.00** | 0.00 |
| WSSP | 47 | 0.05 | 0.00 | 0.95 | 0.00 | 0.00 |  | 54 | 0.00 | 0.00 | 0.00 | **1.00** |
| WVIZ | 13 | 0.08 | 0.00 | 0.85 | 0.08 | 0.00 |  | 13 | 0.00 | 0.00 | 0.00 | **1.00** |
| XOLO | 12 | 0.21 | 0.25 | 0.38 | 0.17 | 0.00 |  | 17 | 0.26 | 0.00 | 0.56 | 0.18 |
| YORK combined | 112 | 0.01 | 0.00 | 0.99 | 0.00 | 0.00 |  | 129 | 0.21 | 0.00 | 0.78 | 0.01 |
| YORK (UK) | 14 | 0.00 | 0.00 | **1.00** | 0.00 | 0.00 |  | 24 | 0.15 | 0.02 | 0.83 | 0.00 |
| YORK (US) | 98 | 0.02 | 0.00 | 0.98 | 0.00 | 0.00 |  | 105 | 0.22 | 0.00 | 0.77 | 0.01 |

**S2 Table (b).** Allele frequencies for the brown (*TYRP1*) and dominant black (*CBD103*) genes. Breeds fixed for a single allele at any gene are indicated with bold text.

|  | ***TYRP1*** | | | |  | ***CBD103*** | | |
| --- | --- | --- | --- | --- | --- | --- | --- | --- |
| **Breed** | **n** | ***B*** | ***b^s^*** | ***b^c^*** |  | **n** | ***K^B^* or *k^br^*** | ***k^y^*** |
| ABUL | 39 | 0.73 | 0.00 | 0.27 |  | 39 | 0.32 | 0.68 |
| ACKR | 83 | 0.70 | 0.07 | 0.22 |  | 84 | 0.46 | 0.54 |
| AESK | 59 | 0.98 | 0.02 | 0.00 |  | 59 | 0.29 | 0.71 |
| AFFN | 40 | **1.00** | 0.00 | 0.00 |  | 40 | 0.75 | 0.25 |
| AFGH | 66 | **1.00** | 0.00 | 0.00 |  | 69 | 0.14 | 0.86 |
| AIRT | 58 | **1.00** | 0.00 | 0.00 |  | 59 | 0.00 | **1.00** |
| AKIT | 77 | 0.99 | 0.01 | 0.00 |  | 79 | 0.10 | 0.90 |
| AMAL | 70 | 0.84 | 0.00 | 0.16 |  | 70 | 0.00 | **1.00** |
| AMST | 294 | 0.66 | 0.16 | 0.17 |  | 297 | 0.42 | 0.58 |
| ANAT | 23 | 0.96 | 0.04 | 0.00 |  | 27 | 0.09 | 0.91 |
| AUCD | 41 | 0.95 | 0.05 | 0.00 |  | 45 | 0.03 | 0.97 |
| AUSS | 136 | 0.56 | 0.25 | 0.18 |  | 137 | 0.04 | 0.96 |
| AUST | 27 | **1.00** | 0.00 | 0.00 |  | 27 | 0.00 | **1.00** |
| AWSP | 34 | 0.00 | 0.57 | 0.43 |  | 34 | **1.00** | 0.00 |
| AZWK | 30 | **1.00** | 0.00 | 0.00 |  | 30 | 0.12 | 0.88 |
| BARB | 19 | 0.63 | 0.32 | 0.05 |  | 19 | 0.68 | 0.32 |
| BASS combined | 75 | **1.00** | 0.00 | 0.00 |  | 75 | 0.00 | **1.00** |
| BASS (UK) | 14 | **1.00** | 0.00 | 0.00 |  | 14 | 0.00 | **1.00** |
| BASS (US) | 61 | **1.00** | 0.00 | 0.00 |  | 61 | 0.00 | **1.00** |
| BBLS | 35 | **1.00** | 0.00 | 0.00 |  | 36 | 0.00 | **1.00** |
| BEAG combined | 116 | 0.84 | 0.01 | 0.15 |  | 117 | 0.00 | **1.00** |
| BEAG (UK) | 18 | 0.97 | 0.03 | 0.00 |  | 18 | 0.00 | **1.00** |
| BEAG (US Field) | 54 | 0.74 | 0.01 | 0.25 |  | 55 | 0.00 | **1.00** |
| BEAG (US Show) | 44 | 0.92 | 0.00 | 0.08 |  | 44 | 0.00 | **1.00** |
| BEAU | 29 | **1.00** | 0.00 | 0.00 |  | 29 | 0.00 | **1.00** |
| BEDT | 60 | 0.49 | 0.00 | 0.51 |  | 61 | 0.75 | 0.25 |
| BELS | 46 | **1.00** | 0.00 | 0.00 |  | 46 | 0.93 | 0.07 |
| BERD | 60 | 0.50 | 0.44 | 0.06 |  | 60 | 0.83 | 0.18 |
| BERG | 9 | 0.94 | 0.06 | 0.00 |  | 9 | 0.89 | 0.11 |
| BICH | 81 | 0.96 | 0.04 | 0.00 |  | 82 | 0.18 | 0.82 |
| BIEW | 30 | 0.97 | 0.03 | 0.00 |  | 30 | 0.00 | **1.00** |
| BLAB | 29 | 0.67 | 0.26 | 0.07 |  | 29 | 0.38 | 0.62 |
| BLDH | 44 | 0.61 | 0.00 | 0.39 |  | 44 | 0.00 | **1.00** |
| BMAL | 52 | 0.99 | 0.00 | 0.01 |  | 52 | 0.00 | **1.00** |
| BMD | 17 | **1.00** | 0.00 | 0.00 |  | 17 | 0.00 | **1.00** |
| BOER | 21 | 0.90 | 0.02 | 0.07 |  | 23 | 0.09 | 0.91 |
| BOLO | 19 | 0.97 | 0.00 | 0.03 |  | 20 | 0.15 | 0.85 |
| BORD | 95 | 0.72 | 0.28 | 0.00 |  | 99 | 0.54 | 0.46 |
| BORT | 93 | 0.98 | 0.02 | 0.00 |  | 93 | 0.00 | **1.00** |
| BORZ | 73 | **1.00** | 0.00 | 0.00 |  | 74 | 0.21 | 0.79 |
| BOST | 71 | 0.90 | 0.08 | 0.01 |  | 72 | 0.50 | 0.50 |
| BOUV | 46 | **1.00** | 0.00 | 0.00 |  | 46 | 0.47 | 0.53 |
| BOX | 77 | 0.99 | 0.01 | 0.00 |  | 78 | 0.22 | 0.78 |
| BOYK | 10 | 0.05 | 0.20 | 0.75 |  | 10 | 0.90 | 0.10 |
| BPIC | 11 | **1.00** | 0.00 | 0.00 |  | 11 | 0.27 | 0.73 |
| BRAC | 13 | 0.00 | 0.38 | 0.62 |  | 13 | 0.46 | 0.54 |
| BRIA | 54 | **1.00** | 0.00 | 0.00 |  | 55 | 0.13 | 0.87 |
| BRIT | 53 | 0.12 | 0.34 | 0.54 |  | 53 | 0.77 | 0.23 |
| BRTR | 36 | **1.00** | 0.00 | 0.00 |  | 36 | 0.93 | 0.07 |
| BRUS | 49 | 0.98 | 0.00 | 0.02 |  | 49 | 0.12 | 0.88 |
| BULD | 79 | 0.94 | 0.00 | 0.06 |  | 79 | 0.16 | 0.84 |
| BULM combined | 61 | 0.94 | 0.02 | 0.03 |  | 61 | 0.07 | 0.93 |
| BULM (UK) | 15 | 0.87 | 0.10 | 0.03 |  | 15 | 0.07 | 0.93 |
| BULM (US) | 46 | 0.97 | 0.00 | 0.03 |  | 46 | 0.07 | 0.93 |
| BULT | 60 | 0.93 | 0.03 | 0.03 |  | 63 | 0.38 | 0.62 |
| CAAN | 18 | 0.83 | 0.00 | 0.17 |  | 18 | 0.39 | 0.61 |
| CAIR | 61 | 0.99 | 0.01 | 0.00 |  | 63 | 0.32 | 0.68 |
| CANE | 45 | 0.96 | 0.00 | 0.04 |  | 46 | 0.46 | 0.54 |
| CARD | 52 | 0.97 | 0.03 | 0.00 |  | 53 | 0.38 | 0.62 |
| CASD | 15 | **1.00** | 0.00 | 0.00 |  | 25 | 0.20 | 0.80 |
| CAUC | 34 | 0.93 | 0.07 | 0.00 |  | 35 | 0.09 | 0.91 |
| CCRT | 79 | 0.54 | 0.46 | 0.01 |  | 79 | 0.96 | 0.04 |
| CESK | 17 | 0.97 | 0.03 | 0.00 |  | 17 | 0.50 | 0.50 |
| CHIH | 53 | 0.72 | 0.26 | 0.02 |  | 55 | 0.02 | 0.98 |
| CHIN | 49 | **1.00** | 0.00 | 0.00 |  | 50 | 0.58 | 0.42 |
| CHOW | 56 | 0.98 | 0.00 | 0.02 |  | 56 | 0.16 | 0.84 |
| CIRN | 16 | 0.00 | 0.44 | 0.56 |  | 16 | 0.09 | 0.91 |
| CKCS | 134 | 0.97 | 0.01 | 0.02 |  | 135 | 0.00 | **1.00** |
| CLSP | 57 | 0.00 | 0.42 | 0.58 |  | 57 | 0.30 | 0.70 |
| COLL combined | 92 | 0.99 | 0.01 | 0.00 |  | 93 | 0.00 | **1.00** |
| COLL (UK) | 27 | 0.98 | 0.02 | 0.00 |  | 27 | 0.00 | **1.00** |
| COLL (US) | 65 | **1.00** | 0.00 | 0.00 |  | 66 | 0.00 | **1.00** |
| COOK | 36 | **1.00** | 0.00 | 0.00 |  | 36 | 0.00 | **1.00** |
| COTO | 82 | 0.98 | 0.02 | 0.01 |  | 84 | 0.01 | 0.99 |
| COYO (Eastern) | 28 | 0.98 | 0.02 | 0.00 |  | 29 | 0.00 | **1.00** |
| COYO (Western) | 18 | **1.00** | 0.00 | 0.00 |  | 19 | 0.00 | **1.00** |
| CPBR | 45 | 0.00 | 0.61 | 0.39 |  | 45 | 0.72 | 0.28 |
| CRES | 22 | 0.86 | 0.14 | 0.00 |  | 31 | 0.23 | 0.77 |
| DACH combined | 172 | 0.84 | 0.05 | 0.11 |  | 178 | 0.01 | 0.99 |
| DACH (Miniature Longhair) | 23 | 0.65 | 0.04 | 0.30 |  | 26 | 0.06 | 0.94 |
| DACH (Miniature Shorthair) | 48 | 0.76 | 0.10 | 0.14 |  | 50 | 0.01 | 0.99 |
| DACH (Miniature Wirehair) | 28 | 0.88 | 0.02 | 0.11 |  | 28 | 0.00 | **1.00** |
| DACH (Standard Longhair) | 23 | **1.00** | 0.00 | 0.00 |  | 24 | 0.00 | **1.00** |
| DACH (Standard Shorthair) | 19 | 0.92 | 0.05 | 0.03 |  | 19 | 0.00 | **1.00** |
| DACH (Standard Wirehair) | 31 | 0.90 | 0.02 | 0.08 |  | 31 | 0.00 | **1.00** |
| DALM combined | 46 | 0.60 | 0.40 | 0.00 |  | 46 | 0.97 | 0.03 |
| DALM (UK) | 12 | 0.50 | 0.50 | 0.00 |  | 12 | **1.00** | 0.00 |
| DALM (US) | 34 | 0.63 | 0.37 | 0.00 |  | 34 | 0.96 | 0.04 |
| DANE | 86 | 0.99 | 0.01 | 0.01 |  | 88 | 0.66 | 0.34 |
| DDBX | 31 | 0.03 | 0.97 | 0.00 |  | 34 | 0.00 | **1.00** |
| DDMT | 41 | **1.00** | 0.00 | 0.00 |  | 41 | 0.00 | **1.00** |
| DEER | 37 | 0.99 | 0.01 | 0.00 |  | 38 | 0.50 | 0.50 |
| DING | 12 | 0.92 | 0.08 | 0.00 |  | 12 | 0.00 | **1.00** |
| DOBP | 115 | 0.44 | 0.02 | 0.54 |  | 115 | 0.00 | **1.00** |
| DOGO | 14 | 0.93 | 0.07 | 0.00 |  | 14 | 0.54 | 0.46 |
| ECKR combined | 89 | 0.87 | 0.08 | 0.05 |  | 89 | 0.62 | 0.38 |
| ECKR (Field) | 17 | 0.41 | 0.41 | 0.18 |  | 17 | 0.79 | 0.21 |
| ECKR (Show) | 72 | 0.97 | 0.01 | 0.02 |  | 72 | 0.58 | 0.42 |
| ESET | 59 | 0.93 | 0.07 | 0.00 |  | 63 | 0.40 | 0.60 |
| ESSP combined | 97 | 0.98 | 0.01 | 0.01 |  | 99 | 0.77 | 0.23 |
| ESSP (UK Field) | 32 | 0.22 | 0.30 | 0.48 |  | 33 | 0.85 | 0.15 |
| ESSP (UK Show) | 28 | 0.16 | 0.32 | 0.52 |  | 29 | 0.55 | 0.45 |
| ESSP (US Show) | 37 | 0.32 | 0.53 | 0.15 |  | 37 | 0.86 | 0.14 |
| EURA | 25 | **1.00** | 0.00 | 0.00 |  | 26 | 0.00 | **1.00** |
| FBUL | 67 | 0.99 | 0.01 | 0.00 |  | 68 | 0.41 | 0.59 |
| FCR | 82 | 0.80 | 0.20 | 0.00 |  | 82 | 0.90 | 0.10 |
| FIEL | 41 | 0.10 | 0.17 | 0.73 |  | 41 | 0.60 | 0.40 |
| FLAP | 26 | 0.65 | 0.00 | 0.35 |  | 26 | 0.02 | 0.98 |
| FOXH | 31 | 0.98 | 0.02 | 0.00 |  | 33 | 0.02 | 0.98 |
| GALG | 25 | 0.94 | 0.06 | 0.00 |  | 25 | 0.52 | 0.48 |
| GLEN | 15 | 0.77 | 0.23 | 0.00 |  | 15 | 0.40 | 0.60 |
| GOLD combined | 181 | 0.98 | 0.01 | 0.01 |  | 181 | 0.77 | 0.23 |
| GOLD (UK) | 38 | 0.97 | 0.03 | 0.00 |  | 41 | 0.55 | 0.45 |
| GOLD (US) | 143 | 0.99 | 0.00 | 0.01 |  | 140 | 0.84 | 0.16 |
| GORD | 45 | **1.00** | 0.00 | 0.00 |  | 45 | 0.00 | **1.00** |
| GPIN | 25 | 0.96 | 0.00 | 0.04 |  | 25 | 0.00 | **1.00** |
| GPYR | 51 | 0.98 | 0.01 | 0.01 |  | 52 | 0.01 | 0.99 |
| GREY combined | 105 | 0.97 | 0.00 | 0.02 |  | 109 | 0.39 | 0.61 |
| GREY (Racing) | 81 | 0.96 | 0.01 | 0.03 |  | 84 | 0.40 | 0.60 |
| GREY (Show) | 24 | **1.00** | 0.00 | 0.00 |  | 25 | 0.36 | 0.64 |
| GSD | 158 | 0.98 | 0.01 | 0.01 |  | 162 | 0.00 | **1.00** |
| GSHP combined | 73 | 0.00 | 0.71 | 0.29 |  | 74 | 0.99 | 0.01 |
| GSHP (UK) | 36 | 0.00 | 0.83 | 0.17 |  | 37 | **1.00** | 0.00 |
| GSHP (US) | 37 | 0.00 | 0.58 | 0.42 |  | 37 | 0.99 | 0.01 |
| GSNZ | 41 | **1.00** | 0.00 | 0.00 |  | 44 | 0.75 | 0.25 |
| GSPZ combined | 46 | 0.48 | 0.47 | 0.05 |  | 46 | 0.28 | 0.72 |
| GSPZ (UK) | 38 | 0.47 | 0.46 | 0.07 |  | 38 | 0.33 | 0.67 |
| GSPZ (US) | 8 | 0.50 | 0.50 | 0.00 |  | 8 | 0.06 | 0.94 |
| GWHP | 33 | 0.02 | 0.67 | 0.32 |  | 33 | 0.98 | 0.02 |
| HARR | 33 | **1.00** | 0.00 | 0.00 |  | 33 | 0.00 | **1.00** |
| HAVA | 61 | 0.87 | 0.07 | 0.06 |  | 61 | 0.27 | 0.73 |
| HUSK | 115 | 0.50 | 0.50 | 0.00 |  | 115 | 0.00 | **1.00** |
| IBIZ | 36 | 0.01 | 0.99 | 0.00 |  | 36 | 0.10 | 0.90 |
| ICES | 26 | 0.81 | 0.19 | 0.00 |  | 29 | 0.02 | 0.98 |
| INCA | 19 | 0.61 | 0.16 | 0.24 |  | 21 | 0.17 | 0.83 |
| IRIT | 33 | **1.00** | 0.00 | 0.00 |  | 33 | 0.00 | **1.00** |
| ISET combined | 72 | 0.98 | 0.02 | 0.00 |  | 74 | 0.61 | 0.39 |
| ISET (UK) | 22 | 0.93 | 0.07 | 0.00 |  | 24 | 0.25 | 0.75 |
| ISET (US) | 50 | **1.00** | 0.00 | 0.00 |  | 50 | 0.79 | 0.21 |
| ITGY | 68 | 0.93 | 0.01 | 0.06 |  | 69 | 0.31 | 0.69 |
| IWOF | 65 | 0.99 | 0.01 | 0.00 |  | 65 | 0.40 | 0.60 |
| IWSP | 32 | 0.00 | 0.69 | 0.31 |  | 32 | **1.00** | 0.00 |
| JIND | 11 | 0.86 | 0.00 | 0.14 |  | 11 | 0.00 | **1.00** |
| JSPZ | 21 | **1.00** | 0.00 | 0.00 |  | 21 | 0.00 | **1.00** |
| KEES combined | 53 | **1.00** | 0.00 | 0.00 |  | 54 | 0.00 | **1.00** |
| KEES (UK) | 18 | **1.00** | 0.00 | 0.00 |  | 18 | 0.00 | **1.00** |
| KEES (US) | 35 | **1.00** | 0.00 | 0.00 |  | 36 | 0.00 | **1.00** |
| KELP | 60 | 0.43 | 0.50 | 0.08 |  | 60 | 0.42 | 0.58 |
| KERY | 41 | **1.00** | 0.00 | 0.00 |  | 41 | 0.65 | 0.35 |
| KKLG | 43 | 0.81 | 0.09 | 0.09 |  | 43 | 0.34 | 0.66 |
| KOMO | 26 | **1.00** | 0.00 | 0.00 |  | 26 | 0.15 | 0.85 |
| KOOL | 41 | 0.49 | 0.39 | 0.12 |  | 42 | 0.50 | 0.50 |
| KUVZ | 15 | **1.00** | 0.00 | 0.00 |  | 15 | 0.37 | 0.63 |
| LAB combined | 376 | 0.61 | 0.32 | 0.08 |  | 376 | 0.92 | 0.08 |
| LAB (UK Show) | 107 | 0.61 | 0.38 | 0.01 |  | 105 | 0.93 | 0.07 |
| LAB (US Field) | 177 | 0.49 | 0.37 | 0.14 |  | 179 | 0.93 | 0.07 |
| LAB (US Show) | 92 | 0.83 | 0.15 | 0.03 |  | 92 | 0.90 | 0.10 |
| LAGO | 134 | 0.00 | 0.29 | 0.71 |  | 139 | 0.82 | 0.18 |
| LAKE | 44 | 0.98 | 0.02 | 0.00 |  | 45 | 0.01 | 0.99 |
| LANC | 10 | 0.90 | 0.10 | 0.00 |  | 11 | 0.00 | **1.00** |
| LEOP | 26 | 0.48 | 0.42 | 0.10 |  | 25 | 0.26 | 0.74 |
| LHAS combined | 56 | 0.93 | 0.07 | 0.00 |  | 60 | 0.05 | 0.95 |
| LHAS (UK) | 16 | **1.00** | 0.00 | 0.00 |  | 18 | 0.08 | 0.92 |
| LHAS (US) | 40 | 0.90 | 0.10 | 0.00 |  | 42 | 0.04 | 0.96 |
| LMUN | 21 | 0.81 | 0.00 | 0.19 |  | 21 | 0.88 | 0.12 |
| LOWC | 37 | 0.65 | 0.32 | 0.03 |  | 37 | 0.01 | 0.99 |
| MALT combined | 92 | 0.97 | 0.02 | 0.02 |  | 95 | 0.05 | 0.95 |
| MALT (UK) | 19 | **1.00** | 0.00 | 0.00 |  | 21 | 0.00 | **1.00** |
| MALT (US) | 73 | 0.96 | 0.02 | 0.02 |  | 74 | 0.06 | 0.94 |
| MANT combined | 35 | **1.00** | 0.00 | 0.00 |  | 35 | 0.00 | **1.00** |
| MANT (UK) | 19 | **1.00** | 0.00 | 0.00 |  | 19 | 0.00 | **1.00** |
| MANT (US) | 16 | **1.00** | 0.00 | 0.00 |  | 16 | 0.00 | **1.00** |
| MARM | 22 | **1.00** | 0.00 | 0.00 |  | 22 | 0.23 | 0.77 |
| MAST | 58 | **1.00** | 0.00 | 0.00 |  | 59 | 0.08 | 0.92 |
| MBLT | 28 | **1.00** | 0.00 | 0.00 |  | 28 | 0.38 | 0.63 |
| MCNB | 28 | 0.68 | 0.29 | 0.04 |  | 28 | 0.59 | 0.41 |
| MGAG | 23 | **1.00** | 0.00 | 0.00 |  | 24 | 0.31 | 0.69 |
| MIKI | 22 | 0.77 | 0.23 | 0.00 |  | 22 | 0.23 | 0.77 |
| MPIN | 52 | 0.81 | 0.19 | 0.00 |  | 52 | 0.00 | **1.00** |
| MSNZ | 84 | 0.96 | 0.01 | 0.02 |  | 85 | 0.16 | 0.84 |
| MUDI | 39 | 0.78 | 0.05 | 0.17 |  | 39 | 0.73 | 0.27 |
| NBUH | 12 | **1.00** | 0.00 | 0.00 |  | 12 | 0.00 | **1.00** |
| NEAP | 39 | 0.62 | 0.09 | 0.29 |  | 39 | 0.50 | 0.50 |
| NELK | 91 | **1.00** | 0.00 | 0.00 |  | 91 | 0.00 | **1.00** |
| NEWF | 63 | 0.84 | 0.01 | 0.15 |  | 66 | 0.98 | 0.02 |
| NLUN | 17 | **1.00** | 0.00 | 0.00 |  | 17 | 0.00 | **1.00** |
| NORF | 50 | **1.00** | 0.00 | 0.00 |  | 52 | 0.00 | **1.00** |
| NOWT | 61 | **1.00** | 0.00 | 0.00 |  | 61 | 0.00 | **1.00** |
| NSDT | 38 | 0.12 | 0.01 | 0.87 |  | 38 | 0.00 | **1.00** |
| OES | 67 | **1.00** | 0.00 | 0.00 |  | 68 | 0.75 | 0.25 |
| OTTR | 34 | 0.65 | 0.35 | 0.00 |  | 34 | 0.00 | **1.00** |
| PAP | 80 | 0.98 | 0.02 | 0.00 |  | 81 | 0.15 | 0.85 |
| PBGV | 47 | 0.96 | 0.04 | 0.00 |  | 47 | 0.00 | **1.00** |
| PDCN | 20 | 0.05 | 0.83 | 0.13 |  | 20 | 0.23 | 0.78 |
| PEKE | 56 | 0.99 | 0.01 | 0.00 |  | 58 | 0.07 | 0.93 |
| PEMB combined | 75 | 0.99 | 0.01 | 0.00 |  | 75 | 0.00 | **1.00** |
| PEMB (UK) | 28 | 0.98 | 0.02 | 0.00 |  | 28 | 0.00 | **1.00** |
| PEMB (US) | 47 | **1.00** | 0.00 | 0.00 |  | 47 | 0.00 | **1.00** |
| PHAR | 20 | 0.00 | 0.60 | 0.40 |  | 21 | 0.00 | **1.00** |
| PLOT | 25 | 0.92 | 0.06 | 0.02 |  | 24 | 0.46 | 0.54 |
| PNTR | 77 | 0.53 | 0.32 | 0.14 |  | 77 | 0.99 | 0.01 |
| POLG | 21 | 0.98 | 0.02 | 0.00 |  | 23 | 0.11 | 0.89 |
| Poodle combined | 275 | 0.81 | 0.07 | 0.13 |  | 281 | 0.74 | 0.26 |
| MEPOO | 17 | 0.79 | 0.06 | 0.15 |  | 18 | 0.72 | 0.28 |
| MPOO | 126 | 0.80 | 0.08 | 0.12 |  | 130 | 0.57 | 0.43 |
| SPOO | 86 | 0.90 | 0.09 | 0.01 |  | 86 | 0.91 | 0.09 |
| TPOO | 46 | 0.64 | 0.02 | 0.34 |  | 47 | 0.89 | 0.11 |
| POM combined | 90 | 0.88 | 0.12 | 0.01 |  | 90 | 0.08 | 0.87 |
| POM (UK) | 14 | 0.96 | 0.00 | 0.04 |  | 14 | 0.11 | 0.89 |
| POM (US) | 76 | 0.86 | 0.14 | 0.00 |  | 76 | 0.14 | 0.86 |
| PONS | 21 | 0.67 | 0.33 | 0.00 |  | 21 | 0.00 | 1.00 |
| PRES | 29 | 0.97 | 0.00 | 0.03 |  | 30 | 0.43 | 0.57 |
| PRUS | 123 | 0.94 | 0.04 | 0.02 |  | 124 | 0.03 | 0.97 |
| PTWD | 50 | 0.67 | 0.25 | 0.08 |  | 50 | 0.95 | 0.05 |
| PUG | 59 | **1.00** | 0.00 | 0.00 |  | 59 | 0.14 | 0.86 |
| PULI | 39 | **1.00** | 0.00 | 0.00 |  | 39 | 0.76 | 0.24 |
| PUMI | 51 | 0.96 | 0.04 | 0.00 |  | 51 | 0.71 | 0.29 |
| PYRS | 14 | **1.00** | 0.00 | 0.00 |  | 14 | 0.36 | 0.64 |
| REDB | 9 | **1.00** | 0.00 | 0.00 |  | 9 | 0.00 | **1.00** |
| RHOD | 63 | 0.71 | 0.29 | 0.00 |  | 64 | 0.00 | **1.00** |
| ROTT | 116 | 0.99 | 0.01 | 0.00 |  | 118 | 0.00 | **1.00** |
| RUSS | 89 | 0.94 | 0.04 | 0.02 |  | 89 | 0.03 | 0.97 |
| RWST | 10 | 0.95 | 0.05 | 0.00 |  | 10 | 0.40 | 0.60 |
| SALU | 116 | 0.81 | 0.17 | 0.02 |  | 117 | 0.01 | 0.99 |
| SAMO | 18 | **1.00** | 0.00 | 0.00 |  | 18 | 0.39 | 0.61 |
| SCOT | 69 | 0.99 | 0.01 | 0.00 |  | 71 | 0.50 | 0.50 |
| SCWT combined | 52 | 0.88 | 0.12 | 0.00 |  | 52 | **1.00** | 0.00 |
| SCWT (UK) | 5 | 0.80 | 0.20 | 0.00 |  | 5 | 0.00 | **1.00** |
| SCWT (US) | 47 | 0.89 | 0.11 | 0.00 |  | 47 | 0.00 | **1.00** |
| SEAL | 29 | **1.00** | 0.00 | 0.00 |  | 29 | 0.31 | 0.69 |
| SFOX | 45 | **1.00** | 0.00 | 0.00 |  | 47 | 0.00 | **1.00** |
| SHAR | 61 | 0.74 | 0.19 | 0.07 |  | 63 | 0.17 | 0.83 |
| SHIB | 64 | **1.00** | 0.00 | 0.00 |  | 65 | 0.00 | **1.00** |
| SHIH | 89 | 0.89 | 0.11 | 0.00 |  | 90 | 0.25 | 0.75 |
| SILK | 45 | 0.96 | 0.01 | 0.03 |  | 46 | 0.00 | **1.00** |
| SKIP combined | 50 | 0.96 | 0.04 | 0.00 |  | 50 | 0.99 | 0.01 |
| SKIP (UK) | 6 | **1.00** | 0.00 | 0.00 |  | 6 | **1.00** | 0.00 |
| SKIP (US) | 44 | 0.95 | 0.05 | 0.00 |  | 44 | 0.99 | 0.01 |
| SKYE | 11 | 0.95 | 0.05 | 0.00 |  | 11 | 0.41 | 0.59 |
| SLOU | 21 | 0.93 | 0.07 | 0.00 |  | 21 | 0.14 | 0.86 |
| SMUN | 8 | 0.00 | 0.44 | 0.56 |  | 8 | 0.81 | 0.19 |
| SPIN | 54 | 0.00 | 0.64 | 0.36 |  | 54 | 0.98 | 0.02 |
| SPWD | 14 | 0.36 | 0.11 | 0.54 |  | 14 | 0.79 | 0.21 |
| SSHP combined | 87 | **1.00** | 0.00 | 0.00 |  | 90 | 0.00 | **1.00** |
| SSHP (UK) | 32 | **1.00** | 0.00 | 0.00 |  | 34 | 0.00 | **1.00** |
| SSHP (US) | 55 | **1.00** | 0.00 | 0.00 |  | 56 | 0.00 | **1.00** |
| SSNZ | 32 | **1.00** | 0.00 | 0.00 |  | 32 | 0.05 | 0.95 |
| STAF | 73 | 0.95 | 0.01 | 0.05 |  | 74 | 0.39 | 0.61 |
| SUSX | 18 | 0.00 | 0.64 | 0.36 |  | 18 | **1.00** | 0.00 |
| SVAL | 38 | **1.00** | 0.00 | 0.00 |  | 38 | 0.00 | **1.00** |
| TAIG | 22 | **1.00** | 0.00 | 0.00 |  | 22 | 0.64 | 0.36 |
| TENT | 10 | 0.75 | 0.20 | 0.05 |  | 10 | 0.05 | 0.95 |
| TERV | 39 | **1.00** | 0.00 | 0.00 |  | 39 | 0.00 | **1.00** |
| TIBM combined | 93 | 0.96 | 0.03 | 0.01 |  | 97 | 0.02 | 0.98 |
| TIBM (China) | 57 | 0.94 | 0.04 | 0.02 |  | 61 | 0.02 | 0.98 |
| TIBM (US) | 36 | **1.00** | 0.00 | 0.00 |  | 36 | 0.03 | 0.97 |
| TIBS | 49 | 0.95 | 0.00 | 0.05 |  | 49 | 0.01 | 0.99 |
| TIBT | 67 | 0.93 | 0.06 | 0.01 |  | 69 | 0.30 | 0.70 |
| TMNT | 39 | 0.99 | 0.01 | 0.00 |  | 39 | 0.00 | **1.00** |
| TREE | 45 | 0.96 | 0.02 | 0.02 |  | 45 | 0.01 | 0.99 |
| VIZS combined | 90 | 0.00 | 0.21 | 0.79 |  | 92 | 0.58 | 0.42 |
| VIZS (UK) | 26 | 0.00 | 0.12 | 0.88 |  | 28 | 0.39 | 0.61 |
| VIZS (US) | 64 | 0.00 | 0.24 | 0.76 |  | 64 | 0.66 | 0.34 |
| VPIN | 18 | 0.89 | 0.11 | 0.00 |  | 19 | 0.34 | 0.66 |
| WEIM combined | 61 | 0.02 | 0.16 | 0.83 |  | 64 | 0.93 | 0.07 |
| WEIM (UK) | 19 | 0.00 | 0.18 | 0.82 |  | 21 | 0.88 | 0.12 |
| WEIM (US) | 42 | 0.02 | 0.14 | 0.83 |  | 43 | 0.95 | 0.05 |
| WELT (UK) | 17 | **1.00** | 0.00 | 0.00 |  | 17 | 0.00 | **1.00** |
| WELT (US) | 48 | **1.00** | 0.00 | 0.00 |  | 49 | 0.00 | **1.00** |
| WFOX | 31 | 0.97 | 0.03 | 0.00 |  | 31 | 0.00 | **1.00** |
| WHIP combined | 65 | **1.00** | 0.00 | 0.00 |  | 66 | 0.00 | **1.00** |
| WHIP (Racing) | 8 | 0.94 | 0.06 | 0.00 |  | 9 | 0.39 | 0.61 |
| WHIP (UK Show) | 36 | **1.00** | 0.00 | 0.00 |  | 37 | 0.30 | 0.70 |
| WHIP (US Show) | 30 | **1.00** | 0.00 | 0.00 |  | 30 | 0.28 | 0.72 |
| WHPG | 20 | 0.00 | 0.60 | 0.40 |  | 20 | 0.88 | 0.13 |
| WHWT | 74 | **1.00** | 0.00 | 0.00 |  | 77 | 0.50 | 0.50 |
| WOLF | 12 | **1.00** | 0.00 | 0.00 |  | 12 | 0.00 | **1.00** |
| WSSP | 53 | 0.93 | 0.00 | 0.07 |  | 54 | 0.72 | 0.28 |
| WVIZ | 17 | 0.00 | 0.35 | 0.65 |  | 17 | 0.74 | 0.26 |
| XOLO | 17 | 0.82 | 0.15 | 0.03 |  | 17 | 0.09 | 0.91 |
| YORK combined | 131 | 0.96 | 0.02 | 0.02 |  | 131 | 0.00 | **1.00** |
| YORK (UK) | 24 | 0.98 | 0.02 | 0.00 |  | 24 | 0.00 | **1.00** |
| YORK (US) | 107 | 0.96 | 0.02 | 0.02 |  | 107 | 0.00 | **1.00** |

**S2 Table (c).** Allele frequencies for the white spotting (*MITF*), harlequin (*PSMB71*), and saddle tan (*RALY*) genes. Breeds fixed for a single allele at any gene are indicated with bold text.

|  | ***MITF*** | | |  | ***PSMB71*** | | |  | ***RALY*** | | |
| --- | --- | --- | --- | --- | --- | --- | --- | --- | --- | --- | --- |
| **Breed** | **n** | ***S*** | ***s^p^*** |  | **n** | ***H*** | ***h*** |  | **n** | ***No Duplication*** | ***Duplication*** |
| ABUL | 39 | 0.35 | 0.65 |  | 39 | **1.00** | 0.00 |  | 39 | 0.03 | 0.97 |
| ACKR | 83 | 0.45 | 0.55 |  | 84 | **1.00** | 0.00 |  | 84 | 0.08 | 0.92 |
| AESK | 59 | 0.08 | 0.92 |  | 59 | **1.00** | 0.00 |  | 59 | 0.34 | 0.66 |
| AFFN | 40 | **1.00** | 0.00 |  | 40 | **1.00** | 0.00 |  | 40 | 0.14 | 0.86 |
| AFGH | 69 | **1.00** | 0.00 |  | 69 | **1.00** | 0.00 |  | 69 | 0.00 | **1.00** |
| AIRT | 59 | **1.00** | 0.00 |  | 59 | **1.00** | 0.00 |  | 58 | 0.00 | **1.00** |
| AKIT | 78 | 0.06 | 0.94 |  | 79 | **1.00** | 0.00 |  | 79 | 0.03 | 0.97 |
| AMAL | 70 | 0.44 | 0.56 |  | 70 | **1.00** | 0.00 |  | 68 | 0.04 | 0.96 |
| AMST | 297 | 0.72 | 0.28 |  | 297 | **1.00** | 0.00 |  | 293 | 0.36 | 0.64 |
| ANAT | 27 | 0.56 | 0.44 |  | 27 | **1.00** | 0.00 |  | 27 | 0.30 | 0.70 |
| AUCD | 45 | 0.00 | **1.00** |  | 45 | **1.00** | 0.00 |  | 44 | 0.48 | 0.52 |
| AUSS | 137 | 0.94 | 0.06 |  | 137 | **1.00** | 0.00 |  | 136 | 0.92 | 0.08 |
| AUST | 27 | **1.00** | 0.00 |  | 27 | **1.00** | 0.00 |  | 27 | 0.00 | **1.00** |
| AWSP | 34 | **1.00** | 0.00 |  | 34 | **1.00** | 0.00 |  | 32 | 0.16 | 0.84 |
| AZWK | 30 | 0.93 | 0.07 |  | 30 | **1.00** | 0.00 |  | 30 | 0.00 | **1.00** |
| BARB | 19 | **1.00** | 0.00 |  | 19 | **1.00** | 0.00 |  | 18 | 0.72 | 0.28 |
| BASS combined | 74 | 0.02 | 0.98 |  | 75 | **1.00** | 0.00 |  | 75 | 0.31 | 0.69 |
| BASS (UK) | 13 | 0.04 | 0.96 |  | 14 | **1.00** | 0.00 |  | 14 | 0.36 | 0.64 |
| BASS (US) | 61 | 0.02 | 0.98 |  | 61 | **1.00** | 0.00 |  | 61 | 0.30 | 0.70 |
| BBLS | 36 | **1.00** | 0.00 |  | 36 | **1.00** | 0.00 |  | 36 | 0.25 | 0.75 |
| BEAG combined | 117 | 0.09 | 0.91 |  | 117 | **1.00** | 0.00 |  | 116 | 0.02 | 0.98 |
| BEAG (UK) | 18 | 0.00 | **1.00** |  | 18 | **1.00** | 0.00 |  | 17 | 0.00 | **1.00** |
| BEAG (US Field) | 55 | 0.15 | 0.85 |  | 55 | **1.00** | 0.00 |  | 55 | 0.05 | 0.95 |
| BEAG (US Show) | 44 | 0.06 | 0.94 |  | 44 | **1.00** | 0.00 |  | 44 | 0.00 | **1.00** |
| BEAU | 29 | 0.95 | 0.05 |  | 29 | **1.00** | 0.00 |  | 29 | 0.72 | 0.28 |
| BEDT | 61 | **1.00** | 0.00 |  | 61 | **1.00** | 0.00 |  | 61 | 0.86 | 0.14 |
| BELS | 46 | 0.91 | 0.09 |  | 46 | **1.00** | 0.00 |  | 46 | 0.08 | 0.92 |
| BERD | 60 | 0.93 | 0.07 |  | 60 | **1.00** | 0.00 |  | 59 | 0.60 | 0.40 |
| BERG | 9 | **1.00** | 0.00 |  | 9 | **1.00** | 0.00 |  | 8 | 0.81 | 0.19 |
| BICH | 81 | 0.06 | 0.94 |  | 82 | **1.00** | 0.00 |  | 76 | 0.20 | 0.80 |
| BIEW | 30 | 0.08 | 0.92 |  | 30 | **1.00** | 0.00 |  | 29 | 0.21 | 0.79 |
| BLAB | 29 | 0.09 | 0.91 |  | 29 | **1.00** | 0.00 |  | 29 | 0.24 | 0.76 |
| BLDH | 44 | **1.00** | 0.00 |  | 44 | **1.00** | 0.00 |  | 43 | 0.07 | 0.93 |
| BMAL | 52 | **1.00** | 0.00 |  | 52 | **1.00** | 0.00 |  | 52 | 0.06 | 0.94 |
| BMD | 17 | **1.00** | 0.00 |  | 17 | **1.00** | 0.00 |  | 17 | 0.82 | 0.18 |
| BOER | 23 | 0.91 | 0.09 |  | 23 | **1.00** | 0.00 |  | 22 | 0.00 | **1.00** |
| BOLO | 20 | 0.20 | 0.80 |  | 20 | **1.00** | 0.00 |  | 18 | 0.72 | 0.28 |
| BORD | 99 | 0.89 | 0.11 |  | 98 | **1.00** | 0.00 |  | 96 | 0.62 | 0.38 |
| BORT | 94 | **1.00** | 0.00 |  | 94 | **1.00** | 0.00 |  | 94 | 0.00 | **1.00** |
| BORZ | 74 | 0.06 | 0.94 |  | 74 | **1.00** | 0.00 |  | 72 | 0.16 | 0.84 |
| BOST | 72 | 0.94 | 0.06 |  | 72 | **1.00** | 0.00 |  | 72 | 0.03 | 0.97 |
| BOUV | 46 | **1.00** | 0.00 |  | 46 | **1.00** | 0.00 |  | 46 | 0.11 | 0.89 |
| BOX | 78 | 0.61 | 0.39 |  | 78 | **1.00** | 0.00 |  | 78 | 0.00 | **1.00** |
| BOYK | 10 | **1.00** | 0.00 |  | 10 | **1.00** | 0.00 |  | 10 | 0.45 | 0.55 |
| BPIC | 11 | **1.00** | 0.00 |  | 11 | **1.00** | 0.00 |  | 11 | 0.00 | **1.00** |
| BRAC | 13 | 0.00 | **1.00** |  | 13 | **1.00** | 0.00 |  | 13 | 0.31 | 0.69 |
| BRIA | 55 | 0.93 | 0.07 |  | 55 | **1.00** | 0.00 |  | 55 | 0.16 | 0.84 |
| BRIT | 53 | 0.00 | **1.00** |  | 53 | **1.00** | 0.00 |  | 52 | 0.62 | 0.38 |
| BRTR | 36 | 0.96 | 0.04 |  | 36 | **1.00** | 0.00 |  | 36 | 0.90 | 0.10 |
| BRUS | 49 | **1.00** | 0.00 |  | 49 | **1.00** | 0.00 |  | 49 | 0.14 | 0.86 |
| BULD | 78 | 0.76 | 0.24 |  | 79 | **1.00** | 0.00 |  | 79 | 0.03 | 0.97 |
| BULM combined | 61 | **1.00** | 0.00 |  | 61 | **1.00** | 0.00 |  | 61 | 0.00 | **1.00** |
| BULM (UK) | 15 | **1.00** | 0.00 |  | 15 | **1.00** | 0.00 |  | 15 | 0.00 | **1.00** |
| BULM (US) | 46 | **1.00** | 0.00 |  | 46 | **1.00** | 0.00 |  | 46 | 0.00 | **1.00** |
| BULT | 63 | 0.27 | 0.73 |  | 62 | **1.00** | 0.00 |  | 63 | 0.55 | 0.45 |
| CAAN | 18 | 0.42 | 0.58 |  | 18 | **1.00** | 0.00 |  | 18 | 0.28 | 0.72 |
| CAIR | 63 | **1.00** | 0.00 |  | 63 | **1.00** | 0.00 |  | 63 | 0.01 | 0.99 |
| CANE | 46 | 0.99 | 0.01 |  | 46 | **1.00** | 0.00 |  | 45 | 0.28 | 0.72 |
| CARD | 53 | **1.00** | 0.00 |  | 53 | **1.00** | 0.00 |  | 52 | 0.29 | 0.71 |
| CASD | 21 | 0.33 | 0.67 |  | 25 | **1.00** | 0.00 |  | 25 | 0.08 | 0.92 |
| CAUC | 35 | 0.84 | 0.16 |  | 35 | **1.00** | 0.00 |  | 35 | 0.40 | 0.60 |
| CCRT | 79 | **1.00** | 0.00 |  | 79 | **1.00** | 0.00 |  | 76 | 0.86 | 0.14 |
| CESK | 17 | **1.00** | 0.00 |  | 17 | **1.00** | 0.00 |  | 17 | 0.18 | 0.82 |
| CHIH | 54 | 0.58 | 0.42 |  | 55 | **1.00** | 0.00 |  | 55 | 0.50 | 0.50 |
| CHIN | 50 | 0.00 | **1.00** |  | 50 | **1.00** | 0.00 |  | 49 | 0.64 | 0.36 |
| CHOW | 56 | **1.00** | 0.00 |  | 56 | **1.00** | 0.00 |  | 56 | 0.08 | 0.92 |
| CIRN | 16 | **1.00** | 0.00 |  | 16 | **1.00** | 0.00 |  | 16 | 0.03 | 0.97 |
| CKCS | 134 | 0.19 | 0.81 |  | 135 | **1.00** | 0.00 |  | 135 | 0.92 | 0.08 |
| CLSP | 57 | 0.00 | **1.00** |  | 57 | **1.00** | 0.00 |  | 51 | 0.45 | 0.55 |
| COLL combined | 93 | 0.92 | 0.08 |  | 93 | **1.00** | 0.00 |  | 92 | 0.73 | 0.27 |
| COLL (UK) | 27 | **1.00** | 0.00 |  | 27 | **1.00** | 0.00 |  | 26 | 0.58 | 0.42 |
| COLL (US) | 66 | 0.89 | 0.11 |  | 66 | **1.00** | 0.00 |  | 66 | 0.80 | 0.20 |
| COOK | 36 | **1.00** | 0.00 |  | 36 | **1.00** | 0.00 |  | 36 | 0.10 | 0.90 |
| COTO | 84 | 0.01 | 0.99 |  | 84 | **1.00** | 0.00 |  | 84 | 0.15 | 0.85 |
| COYO (Eastern) | 28 | 0.88 | 0.13 |  | 25 | **1.00** | 0.00 |  | 24 | 0.17 | 0.83 |
| COYO (Western) | 19 | **1.00** | 0.00 |  | 19 | **1.00** | 0.00 |  | 17 | 0.00 | **1.00** |
| CPBR | 45 | **1.00** | 0.00 |  | 45 | **1.00** | 0.00 |  | 45 | 0.00 | **1.00** |
| CRES | 28 | 0.66 | 0.34 |  | 31 | **1.00** | 0.00 |  | 31 | 0.27 | 0.73 |
| DACH combined | 181 | 0.82 | 0.18 |  | 180 | **1.00** | 0.00 |  | 173 | 0.71 | 0.29 |
| DACH (Miniature Longhair) | 27 | 0.61 | 0.39 |  | 26 | **1.00** | 0.00 |  | 24 | 0.71 | 0.29 |
| DACH (Miniature Shorthair) | 52 | 0.68 | 0.32 |  | 52 | **1.00** | 0.00 |  | 47 | 0.72 | 0.28 |
| DACH (Miniature Wirehair) | 28 | 0.88 | 0.13 |  | 28 | **1.00** | 0.00 |  | 28 | 0.63 | 0.38 |
| DACH (Standard Longhair) | 24 | 0.92 | 0.08 |  | 24 | **1.00** | 0.00 |  | 24 | 0.46 | 0.54 |
| DACH (Standard Shorthair) | 19 | **1.00** | 0.00 |  | 19 | **1.00** | 0.00 |  | 19 | 0.79 | 0.21 |
| DACH (Standard Wirehair) | 31 | **1.00** | 0.00 |  | 31 | **1.00** | 0.00 |  | 31 | 0.92 | 0.08 |
| DALM combined | 46 | 0.00 | **1.00** |  | 46 | **1.00** | 0.00 |  | 46 | 0.93 | 0.07 |
| DALM (UK) | 12 | 0.00 | **1.00** |  | 12 | **1.00** | 0.00 |  | 12 | 0.92 | 0.08 |
| DALM (US) | 34 | 0.00 | **1.00** |  | 34 | **1.00** | 0.00 |  | 34 | 0.94 | 0.06 |
| DANE | 88 | 0.94 | 0.06 |  | 88 | 0.79 | 0.21 |  | 88 | 0.14 | 0.86 |
| DDBX | 34 | **1.00** | 0.00 |  | 34 | **1.00** | 0.00 |  | 34 | 0.00 | **1.00** |
| DDMT | 41 | **1.00** | 0.00 |  | 41 | **1.00** | 0.00 |  | 41 | 0.52 | 0.48 |
| DEER | 38 | **1.00** | 0.00 |  | 38 | **1.00** | 0.00 |  | 38 | 0.11 | 0.89 |
| DING | 12 | 0.13 | 0.88 |  | 12 | **1.00** | 0.00 |  | 12 | 0.17 | 0.83 |
| DOBP | 115 | 0.57 | 0.43 |  | 115 | **1.00** | 0.00 |  | 110 | 0.75 | 0.25 |
| DOGO | 14 | 0.00 | **1.00** |  | 14 | **1.00** | 0.00 |  | 14 | 0.04 | 0.96 |
| ECKR combined | 89 | 0.30 | 0.70 |  | 89 | **1.00** | 0.00 |  | 89 | 0.66 | 0.34 |
| ECKR (Field) | 17 | 0.53 | 0.47 |  | 17 | **1.00** | 0.00 |  | 17 | 0.35 | 0.65 |
| ECKR (Show) | 72 | 0.25 | 0.75 |  | 72 | **1.00** | 0.00 |  | 72 | 0.73 | 0.27 |
| ESET | 61 | 0.03 | 0.97 |  | 63 | **1.00** | 0.00 |  | 63 | 0.67 | 0.33 |
| ESSP combined | 99 | 0.01 | 0.99 |  | 99 | **1.00** | 0.00 |  | 98 | 0.69 | 0.31 |
| ESSP (UK Field) | 33 | 0.02 | 0.98 |  | 33 | **1.00** | 0.00 |  | 32 | 0.41 | 0.59 |
| ESSP (UK Show) | 29 | 0.00 | **1.00** |  | 29 | **1.00** | 0.00 |  | 29 | 0.69 | 0.31 |
| ESSP (US Show) | 37 | 0.00 | **1.00** |  | 37 | **1.00** | 0.00 |  | 37 | 0.93 | 0.07 |
| EURA | 25 | 0.98 | 0.02 |  | 26 | **1.00** | 0.00 |  | 26 | 0.35 | 0.65 |
| FBUL | 68 | 0.63 | 0.38 |  | 68 | **1.00** | 0.00 |  | 68 | 0.05 | 0.95 |
| FCR | 82 | **1.00** | 0.00 |  | 82 | **1.00** | 0.00 |  | 81 | 0.12 | 0.88 |
| FIEL | 42 | 0.89 | 0.11 |  | 42 | **1.00** | 0.00 |  | 41 | 0.98 | 0.02 |
| FLAP | 25 | 0.86 | 0.14 |  | 26 | **1.00** | 0.00 |  | 26 | 0.40 | 0.60 |
| FOXH | 32 | 0.20 | 0.80 |  | 33 | **1.00** | 0.00 |  | 32 | 0.05 | 0.95 |
| GALG | 25 | 0.96 | 0.04 |  | 25 | **1.00** | 0.00 |  | 25 | 0.10 | 0.90 |
| GLEN | 15 | 0.97 | 0.03 |  | 15 | **1.00** | 0.00 |  | 14 | 0.00 | **1.00** |
| GOLD combined | 184 | **1.00** | 0.00 |  | 184 | **1.00** | 0.00 |  | 131 | 0.90 | 0.10 |
| GOLD (UK) | 41 | **1.00** | 0.00 |  | 41 | **1.00** | 0.00 |  | 39 | 0.67 | 0.33 |
| GOLD (US) | 143 | **1.00** | 0.00 |  | 143 | **1.00** | 0.00 |  | 140 | 0.65 | 0.35 |
| GORD | 45 | **1.00** | 0.00 |  | 45 | **1.00** | 0.00 |  | 45 | **1.00** | 0.00 |
| GPIN | 25 | **1.00** | 0.00 |  | 25 | **1.00** | 0.00 |  | 24 | 0.17 | 0.83 |
| GPYR | 51 | 0.01 | 0.99 |  | 52 | **1.00** | 0.00 |  | 52 | 0.12 | 0.88 |
| GREY combined | 109 | 0.53 | 0.47 |  | 109 | **1.00** | 0.00 |  | 108 | 0.03 | 0.97 |
| GREY (Racing) | 84 | 0.62 | 0.38 |  | 84 | **1.00** | 0.00 |  | 83 | 0.04 | 0.96 |
| GREY (Show) | 25 | 0.22 | 0.78 |  | 25 | **1.00** | 0.00 |  | 25 | 0.00 | **1.00** |
| GSD | 162 | **1.00** | 0.00 |  | 162 | **1.00** | 0.00 |  | 159 | 0.20 | 0.80 |
| GSHP combined | 73 | 0.05 | 0.95 |  | 74 | **1.00** | 0.00 |  | 72 | 0.53 | 0.47 |
| GSHP (UK) | 36 | 0.08 | 0.92 |  | 37 | **1.00** | 0.00 |  | 36 | 0.44 | 0.56 |
| GSHP (US) | 37 | 0.03 | 0.97 |  | 37 | **1.00** | 0.00 |  | 36 | 0.61 | 0.39 |
| GSNZ | 44 | 0.97 | 0.03 |  | 44 | **1.00** | 0.00 |  | 43 | 0.78 | 0.22 |
| GSPZ combined | 46 | 0.59 | 0.41 |  | 46 | **1.00** | 0.00 |  | 46 | 0.17 | 0.83 |
| GSPZ (UK) | 38 | 0.62 | 0.38 |  | 38 | **1.00** | 0.00 |  | 38 | 0.14 | 0.86 |
| GSPZ (US) | 8 | 0.44 | 0.56 |  | 8 | **1.00** | 0.00 |  | 8 | 0.31 | 0.69 |
| GWHP | 33 | 0.05 | 0.95 |  | 33 | **1.00** | 0.00 |  | 33 | 0.91 | 0.09 |
| HARR | 33 | 0.24 | 0.76 |  | 33 | **1.00** | 0.00 |  | 33 | 0.00 | **1.00** |
| HAVA | 61 | 0.34 | 0.66 |  | 61 | **1.00** | 0.00 |  | 60 | 0.65 | 0.35 |
| HUSK | 115 | 0.21 | 0.79 |  | 115 | **1.00** | 0.00 |  | 115 | 0.23 | 0.77 |
| IBIZ | 36 | 0.88 | 0.13 |  | 36 | **1.00** | 0.00 |  | 35 | 0.16 | 0.84 |
| ICES | 26 | 0.79 | 0.21 |  | 29 | **1.00** | 0.00 |  | 29 | 0.34 | 0.66 |
| INCA | 18 | 0.42 | 0.58 |  | 21 | **1.00** | 0.00 |  | 21 | 0.48 | 0.52 |
| IRIT | 33 | **1.00** | 0.00 |  | 33 | **1.00** | 0.00 |  | 33 | 0.03 | 0.97 |
| ISET combined | 75 | **1.00** | 0.00 |  | 73 | **1.00** | 0.00 |  | 74 | 0.86 | 0.14 |
| ISET (UK) | 25 | **1.00** | 0.00 |  | 23 | **1.00** | 0.00 |  | 24 | 0.69 | 0.31 |
| ISET (US) | 50 | **1.00** | 0.00 |  | 50 | **1.00** | 0.00 |  | 50 | 0.95 | 0.05 |
| ITGY | 69 | 0.75 | 0.25 |  | 69 | **1.00** | 0.00 |  | 69 | 0.09 | 0.91 |
| IWOF | 65 | **1.00** | 0.00 |  | 65 | **1.00** | 0.00 |  | 65 | 0.53 | 0.47 |
| IWSP | 32 | **1.00** | 0.00 |  | 32 | **1.00** | 0.00 |  | 32 | 0.84 | 0.16 |
| JIND | 11 | 0.86 | 0.14 |  | 11 | **1.00** | 0.00 |  | 11 | 0.09 | 0.91 |
| JSPZ | 20 | 0.00 | **1.00** |  | 21 | **1.00** | 0.00 |  | 21 | 0.31 | 0.69 |
| KEES combined | 54 | **1.00** | 0.00 |  | 54 | **1.00** | 0.00 |  | 54 | 0.98 | 0.02 |
| KEES (UK) | 18 | **1.00** | 0.00 |  | 18 | **1.00** | 0.00 |  | 18 | 0.97 | 0.03 |
| KEES (US) | 36 | **1.00** | 0.00 |  | 36 | **1.00** | 0.00 |  | 36 | 0.99 | 0.01 |
| KELP | 60 | **1.00** | 0.00 |  | 60 | **1.00** | 0.00 |  | 57 | 0.90 | 0.10 |
| KERY | 41 | **1.00** | 0.00 |  | 41 | **1.00** | 0.00 |  | 41 | 0.22 | 0.78 |
| KKLG | 43 | 0.78 | 0.22 |  | 43 | **1.00** | 0.00 |  | 43 | 0.44 | 0.56 |
| KOMO | 26 | 0.75 | 0.25 |  | 26 | **1.00** | 0.00 |  | 25 | 0.36 | 0.64 |
| KOOL | 42 | 0.98 | 0.02 |  | 42 | **1.00** | 0.00 |  | 42 | 0.76 | 0.24 |
| KUVZ | 15 | 0.83 | 0.17 |  | 15 | **1.00** | 0.00 |  | 14 | 0.68 | 0.32 |
| LAB combined | 380 | **1.00** | 0.00 |  | 380 | **1.00** | 0.00 |  | 366 | 0.86 | 0.14 |
| LAB (UK Show) | 107 | **1.00** | 0.00 |  | 107 | **1.00** | 0.00 |  | 107 | 0.88 | 0.12 |
| LAB (US Field) | 180 | **1.00** | 0.00 |  | 180 | **1.00** | 0.00 |  | 170 | 0.83 | 0.17 |
| LAB (US Show) | 93 | **1.00** | 0.00 |  | 93 | **1.00** | 0.00 |  | 89 | 0.87 | 0.13 |
| LAGO | 136 | 0.21 | 0.79 |  | 139 | **1.00** | 0.00 |  | 136 | 0.86 | 0.14 |
| LAKE | 45 | **1.00** | 0.00 |  | 45 | **1.00** | 0.00 |  | 45 | 0.00 | **1.00** |
| LANC | 11 | **1.00** | 0.00 |  | 11 | **1.00** | 0.00 |  | 10 | 0.65 | 0.35 |
| LEOP | 26 | 0.94 | 0.06 |  | 26 | **1.00** | 0.00 |  | 26 | 0.69 | 0.31 |
| LHAS combined | 60 | 0.34 | 0.43 |  | 60 | **1.00** | 0.00 |  | 58 | 0.10 | 0.90 |
| LHAS (UK) | 18 | 0.50 | 0.50 |  | 18 | **1.00** | 0.00 |  | 18 | 0.03 | 0.97 |
| LHAS (US) | 42 | 0.61 | 0.39 |  | 42 | **1.00** | 0.00 |  | 40 | 0.14 | 0.86 |
| LMUN | 21 | 0.00 | **1.00** |  | 21 | **1.00** | 0.00 |  | 21 | 0.95 | 0.05 |
| LOWC | 37 | 0.93 | 0.07 |  | 37 | **1.00** | 0.00 |  | 37 | 0.03 | 0.97 |
| MALT combined | 92 | 0.02 | 0.98 |  | 95 | **1.00** | 0.00 |  | 92 | 0.44 | 0.56 |
| MALT (UK) | 20 | 0.00 | **1.00** |  | 21 | **1.00** | 0.00 |  | 21 | 0.52 | 0.48 |
| MALT (US) | 72 | 0.03 | 0.97 |  | 74 | **1.00** | 0.00 |  | 71 | 0.42 | 0.58 |
| MANT combined | 35 | **1.00** | 0.00 |  | 35 | **1.00** | 0.00 |  | 34 | **1.00** | 0.00 |
| MANT (UK) | 19 | **1.00** | 0.00 |  | 19 | **1.00** | 0.00 |  | 19 | **1.00** | 0.00 |
| MANT (US) | 16 | **1.00** | 0.00 |  | 16 | **1.00** | 0.00 |  | 15 | **1.00** | 0.00 |
| MARM | 22 | 0.05 | 0.95 |  | 22 | **1.00** | 0.00 |  | 21 | 0.86 | 0.14 |
| MAST | 58 | 0.90 | 0.10 |  | 59 | **1.00** | 0.00 |  | 59 | 0.12 | 0.88 |
| MBLT | 28 | 0.36 | 0.64 |  | 28 | **1.00** | 0.00 |  | 27 | 0.39 | 0.61 |
| MCNB | 28 | 0.95 | 0.05 |  | 28 | **1.00** | 0.00 |  | 28 | 0.57 | 0.43 |
| MGAG | 24 | 0.13 | 0.88 |  | 24 | **1.00** | 0.00 |  | 24 | 0.02 | 0.98 |
| MIKI | 22 | 0.23 | 0.77 |  | 22 | **1.00** | 0.00 |  | 22 | 0.34 | 0.66 |
| MPIN | 52 | **1.00** | 0.00 |  | 52 | **1.00** | 0.00 |  | 51 | 0.79 | 0.21 |
| MSNZ | 85 | 0.96 | 0.04 |  | 85 | **1.00** | 0.00 |  | 83 | 0.92 | 0.08 |
| MUDI | 39 | 0.99 | 0.01 |  | 39 | **1.00** | 0.00 |  | 39 | 0.10 | 0.90 |
| NBUH | 12 | **1.00** | 0.00 |  | 12 | **1.00** | 0.00 |  | 12 | 0.08 | 0.92 |
| NEAP | 39 | **1.00** | 0.00 |  | 39 | **1.00** | 0.00 |  | 39 | 0.29 | 0.71 |
| NELK | 91 | 0.73 | 0.27 |  | 91 | **1.00** | 0.00 |  | 90 | 0.62 | 0.38 |
| NEWF | 66 | 0.67 | 0.33 |  | 66 | **1.00** | 0.00 |  | 63 | 0.39 | 0.61 |
| NLUN | 17 | 0.97 | 0.03 |  | 17 | **1.00** | 0.00 |  | 17 | 0.00 | **1.00** |
| NORF | 52 | **1.00** | 0.00 |  | 52 | **1.00** | 0.00 |  | 52 | 0.00 | **1.00** |
| NOWT | 61 | **1.00** | 0.00 |  | 61 | **1.00** | 0.00 |  | 61 | 0.00 | **1.00** |
| NSDT | 38 | **1.00** | 0.00 |  | 38 | **1.00** | 0.00 |  | 38 | 0.01 | 0.99 |
| OES | 68 | **1.00** | 0.00 |  | 68 | **1.00** | 0.00 |  | 65 | 0.66 | 0.34 |
| OTTR | 34 | 0.97 | 0.03 |  | 34 | **1.00** | 0.00 |  | 34 | 0.16 | 0.84 |
| PAP | 80 | 0.04 | 0.96 |  | 81 | **1.00** | 0.00 |  | 81 | 0.39 | 0.61 |
| PBGV | 47 | 0.18 | 0.82 |  | 47 | **1.00** | 0.00 |  | 45 | 0.76 | 0.24 |
| PDCN | 20 | 0.88 | 0.13 |  | 20 | **1.00** | 0.00 |  | 20 | 0.58 | 0.43 |
| PEKE | 58 | 0.69 | 0.31 |  | 58 | **1.00** | 0.00 |  | 57 | 0.03 | 0.97 |
| PEMB combined | 75 | 0.99 | 0.01 |  | 75 | **1.00** | 0.00 |  | 72 | 0.18 | 0.82 |
| PEMB (UK) | 28 | **1.00** | 0.00 |  | 28 | **1.00** | 0.00 |  | 27 | 0.07 | 0.93 |
| PEMB (US) | 47 | 0.98 | 0.02 |  | 47 | **1.00** | 0.00 |  | 45 | 0.24 | 0.76 |
| PHAR | 21 | **1.00** | 0.00 |  | 21 | **1.00** | 0.00 |  | 21 | 0.00 | **1.00** |
| PLOT | 25 | 0.92 | 0.08 |  | 25 | **1.00** | 0.00 |  | 25 | 0.16 | 0.84 |
| PNTR | 78 | 0.01 | 0.99 |  | 78 | **1.00** | 0.00 |  | 78 | **1.00** | 0.00 |
| POLG | 23 | 0.61 | 0.39 |  | 23 | **1.00** | 0.00 |  | 22 | 0.61 | 0.39 |
| Poodle Combined | 281 | 0.89 | 0.11 |  | 281 | **1.00** | 0.00 |  | 276 | 0.66 | 0.34 |
| MePOO | 18 | 0.92 | 0.08 |  | 18 | **1.00** | 0.00 |  | 18 | 0.83 | 0.17 |
| MPOO | 130 | 0.82 | 0.18 |  | 130 | **1.00** | 0.00 |  | 128 | 0.76 | 0.24 |
| SPOO | 86 | 0.94 | 0.06 |  | 86 | **1.00** | 0.00 |  | 84 | 0.32 | 0.68 |
| TPOO | 47 | **1.00** | 0.00 |  | 47 | **1.00** | 0.00 |  | 46 | 0.96 | 0.04 |
| POM combined | 90 | 0.79 | 0.21 |  | 90 | **1.00** | 0.00 |  | 89 | 0.21 | 0.79 |
| POM (UK) | 14 | 0.89 | 0.11 |  | 14 | **1.00** | 0.00 |  | 14 | 0.11 | 0.89 |
| POM (US) | 76 | 0.77 | 0.23 |  | 76 | **1.00** | 0.00 |  | 76 | 0.22 | 0.78 |
| PONS | 21 | 0.38 | 0.62 |  | 21 | **1.00** | 0.00 |  | 21 | **1.00** | 0.00 |
| PRES | 30 | **1.00** | 0.00 |  | 30 | **1.00** | 0.00 |  | 30 | 0.02 | 0.98 |
| PRUS | 121 | 0.00 | **1.00** |  | 124 | **1.00** | 0.00 |  | 123 | 0.18 | 0.82 |
| PTWD | 50 | 0.83 | 0.17 |  | 50 | **1.00** | 0.00 |  | 49 | 0.33 | 0.67 |
| PUG | 59 | **1.00** | 0.00 |  | 59 | **1.00** | 0.00 |  | 58 | 0.03 | 0.97 |
| PULI | 39 | 0.99 | 0.01 |  | 39 | **1.00** | 0.00 |  | 39 | 0.29 | 0.71 |
| PUMI | 51 | **1.00** | 0.00 |  | 51 | **1.00** | 0.00 |  | 51 | 0.36 | 0.64 |
| PYRS | 14 | **1.00** | 0.00 |  | 14 | **1.00** | 0.00 |  | 14 | 0.04 | 0.96 |
| REDB | 9 | **1.00** | 0.00 |  | 9 | **1.00** | 0.00 |  | 9 | 0.00 | **1.00** |
| RHOD | 64 | **1.00** | 0.00 |  | 64 | **1.00** | 0.00 |  | 62 | 0.02 | 0.98 |
| ROTT | 118 | **1.00** | 0.00 |  | 118 | **1.00** | 0.00 |  | 115 | **1.00** | 0.00 |
| RUSS | 89 | 0.04 | 0.96 |  | 89 | **1.00** | 0.00 |  | 88 | 0.18 | 0.82 |
| RWST | 8 | 0.00 | **1.00** |  | 10 | **1.00** | 0.00 |  | 10 | 0.80 | 0.20 |
| SALU | 116 | 0.78 | 0.22 |  | 117 | **1.00** | 0.00 |  | 116 | 0.11 | 0.89 |
| SAMO | 18 | 0.28 | 0.72 |  | 18 | **1.00** | 0.00 |  | 18 | 0.58 | 0.42 |
| SCOT | 71 | **1.00** | 0.00 |  | 71 | **1.00** | 0.00 |  | 71 | 0.73 | 0.27 |
| SCWT combined | 52 | **1.00** | 0.00 |  | 52 | **1.00** | 0.00 |  | 52 | 0.00 | **1.00** |
| SCWT (UK) | 5 | **1.00** | 0.00 |  | 5 | **1.00** | 0.00 |  | 5 | 0.00 | **1.00** |
| SCWT (US) | 47 | **1.00** | 0.00 |  | 47 | **1.00** | 0.00 |  | 47 | 0.00 | **1.00** |
| SEAL | 29 | 0.00 | **1.00** |  | 29 | **1.00** | 0.00 |  | 29 | 0.12 | 0.88 |
| SFOX | 47 | 0.00 | **1.00** |  | 47 | **1.00** | 0.00 |  | 47 | 0.30 | 0.70 |
| SHAR | 63 | 0.94 | 0.06 |  | 63 | **1.00** | 0.00 |  | 62 | 0.17 | 0.83 |
| SHIB | 65 | 0.95 | 0.05 |  | 65 | **1.00** | 0.00 |  | 63 | 0.29 | 0.71 |
| SHIH | 90 | 0.17 | 0.83 |  | 90 | **1.00** | 0.00 |  | 90 | 0.07 | 0.93 |
| SILK | 46 | 0.99 | 0.01 |  | 46 | **1.00** | 0.00 |  | 46 | 0.00 | **1.00** |
| SKIP combined | 50 | **1.00** | 0.00 |  | 50 | **1.00** | 0.00 |  | 49 | 0.31 | 0.69 |
| SKIP (UK) | 6 | **1.00** | 0.00 |  | 6 | **1.00** | 0.00 |  | 6 | 0.67 | 0.33 |
| SKIP (US) | 44 | **1.00** | 0.00 |  | 44 | **1.00** | 0.00 |  | 43 | 0.26 | 0.74 |
| SKYE | 11 | **1.00** | 0.00 |  | 11 | **1.00** | 0.00 |  | 11 | 0.00 | **1.00** |
| SLOU | 21 | **1.00** | 0.00 |  | 21 | **1.00** | 0.00 |  | 21 | 0.29 | 0.71 |
| SMUN | 8 | 0.00 | **1.00** |  | 8 | **1.00** | 0.00 |  | 8 | **1.00** | 0.00 |
| SPIN | 54 | 0.00 | **1.00** |  | 54 | **1.00** | 0.00 |  | 52 | 0.97 | 0.03 |
| SPWD | 14 | 0.64 | 0.36 |  | 14 | **1.00** | 0.00 |  | 14 | 0.89 | 0.11 |
| SSHP combined | 90 | 0.90 | 0.10 |  | 89 | **1.00** | 0.00 |  | 88 | 0.43 | 0.57 |
| SSHP (UK) | 34 | 0.84 | 0.16 |  | 34 | **1.00** | 0.00 |  | 33 | 0.62 | 0.38 |
| SSHP (US) | 56 | 0.94 | 0.06 |  | 55 | **1.00** | 0.00 |  | 55 | 0.31 | 0.69 |
| SSNZ | 32 | 0.83 | 0.17 |  | 32 | **1.00** | 0.00 |  | 32 | 0.92 | 0.08 |
| STAF | 74 | 0.84 | 0.16 |  | 74 | **1.00** | 0.00 |  | 74 | 0.32 | 0.68 |
| SUSX | 18 | **1.00** | 0.00 |  | 18 | **1.00** | 0.00 |  | 17 | **1.00** | 0.00 |
| SVAL | 38 | **1.00** | 0.00 |  | 38 | **1.00** | 0.00 |  | 38 | 0.95 | 0.05 |
| TAIG | 22 | 0.86 | 0.14 |  | 22 | **1.00** | 0.00 |  | 22 | 0.27 | 0.73 |
| TENT | 10 | 0.05 | 0.95 |  | 10 | **1.00** | 0.00 |  | 10 | 0.65 | 0.35 |
| TERV | 39 | 0.97 | 0.03 |  | 39 | **1.00** | 0.00 |  | 39 | 0.00 | **1.00** |
| TIBM combined | 97 | 0.96 | 0.04 |  | 97 | **1.00** | 0.00 |  | 94 | 0.21 | 0.79 |
| TIBM (China) | 61 | 0.98 | 0.02 |  | 61 | **1.00** | 0.00 |  | 60 | 0.17 | 0.83 |
| TIBM (US) | 36 | 0.94 | 0.06 |  | 36 | **1.00** | 0.00 |  | 34 | 0.28 | 0.72 |
| TIBS | 49 | 0.67 | 0.33 |  | 49 | **1.00** | 0.00 |  | 48 | 0.07 | 0.93 |
| TIBT | 69 | 0.41 | 0.59 |  | 69 | **1.00** | 0.00 |  | 69 | 0.49 | 0.51 |
| TMNT | 39 | **1.00** | 0.00 |  | 39 | **1.00** | 0.00 |  | 38 | **1.00** | 0.00 |
| TREE | 44 | 0.02 | 0.98 |  | 45 | **1.00** | 0.00 |  | 45 | 0.24 | 0.76 |
| VIZS combined | 92 | 0.80 | 0.20 |  | 92 | **1.00** | 0.00 |  | 88 | 0.77 | 0.23 |
| VIZS (UK) | 28 | **1.00** | 0.00 |  | 28 | **1.00** | 0.00 |  | 28 | 0.82 | 0.18 |
| VIZS (US) | 64 | 0.72 | 0.28 |  | 64 | **1.00** | 0.00 |  | 60 | 0.75 | 0.25 |
| VPIN | 16 | 0.00 | **1.00** |  | 19 | **1.00** | 0.00 |  | 19 | 0.26 | 0.74 |
| WEIM combined | 64 | **1.00** | 0.00 |  | 64 | **1.00** | 0.00 |  | 63 | **1.00** | 0.00 |
| WEIM (UK) | 21 | **1.00** | 0.00 |  | 21 | **1.00** | 0.00 |  | 21 | **1.00** | 0.00 |
| WEIM (US) | 43 | **1.00** | 0.00 |  | 43 | **1.00** | 0.00 |  | 42 | **1.00** | 0.00 |
| WELT combined | 66 | **1.00** | 0.00 |  | 66 | **1.00** | 0.00 |  | 67 | 0.00 | **1.00** |
| WELT (UK) | 17 | **1.00** | 0.00 |  | 17 | **1.00** | 0.00 |  | 17 | 0.00 | **1.00** |
| WELT (US) | 49 | **1.00** | 0.00 |  | 49 | **1.00** | 0.00 |  | 49 | 0.00 | **1.00** |
| WFOX | 31 | 0.00 | **1.00** |  | 31 | **1.00** | 0.00 |  | 31 | 0.02 | 0.98 |
| WHIP combined | 75 | 0.29 | 0.71 |  | 76 | **1.00** | 0.00 |  | 76 | 0.00 | **1.00** |
| WHIP (Racing) | 9 | 0.72 | 0.28 |  | 9 | **1.00** | 0.00 |  | 9 | 0.00 | **1.00** |
| WHIP (UK Show) | 36 | 0.38 | 0.63 |  | 37 | **1.00** | 0.00 |  | 37 | 0.00 | **1.00** |
| WHIP (US Show) | 30 | 0.07 | 0.93 |  | 30 | **1.00** | 0.00 |  | 30 | 0.00 | **1.00** |
| WHPG | 20 | 0.00 | **1.00** |  | 20 | **1.00** | 0.00 |  | 20 | 0.70 | 0.30 |
| WHWT | 77 | **1.00** | 0.00 |  | 77 | **1.00** | 0.00 |  | 76 | 0.00 | **1.00** |
| WOLF | 12 | 0.92 | 0.08 |  | 11 | **1.00** | 0.00 |  | 9 | 0.61 | 0.39 |
| WSSP | 54 | 0.00 | **1.00** |  | 54 | **1.00** | 0.00 |  | 53 | 0.42 | 0.58 |
| WVIZ | 17 | 0.97 | 0.03 |  | 17 | **1.00** | 0.00 |  | 16 | 0.78 | 0.22 |
| XOLO | 17 | 0.71 | 0.29 |  | 17 | **1.00** | 0.00 |  | 17 | 0.44 | 0.56 |
| YORK combined | 131 | 0.96 | 0.04 |  | 131 | 0.98 | 0.02 |  | 131 | 0.00 | **1.00** |
| YORK (UK) | 24 | **1.00** | 0.00 |  | 24 | 0.92 | 0.08 |  | 24 | 0.02 | 0.98 |
| YORK (US) | 107 | 0.95 | 0.05 |  | 107 | **1.00** | 0.00 |  | 107 | 0.00 | **1.00** |

**S2 Table (d).** Allele frequencies for hair length (*FGF5*), hair curl (*KRT71*), and ear set. Breeds fixed for a single allele at any gene are indicated with bold text.

|  | ***FGF5*** | | |  | ***KRT71*** | | | |  | **Ear Set** | | |
| --- | --- | --- | --- | --- | --- | --- | --- | --- | --- | --- | --- | --- |
| **Breed** | **n** | ***Short*** | ***Long*** |  | **n** | | ***No-curl*** | ***Curled*** |  | **n** | ***Erect*** | ***Drop*** |
| ABUL | 39 | 0.96 | 0.04 |  | 21 | | **1.00** | 0.00 |  | 39 | 0.83 | 0.17 |
| ACKR | 80 | 0.01 | 0.99 |  | 73 | | 0.97 | 0.03 |  | 84 | 0.00 | **1.00** |
| AESK | 52 | 0.09 | 0.91 |  | 47 | | 0.99 | 0.01 |  | 59 | 0.99 | 0.01 |
| AFFN | 40 | 0.95 | 0.05 |  | 39 | | 0.99 | 0.01 |  | 40 | 0.89 | 0.11 |
| AFGH | 63 | 0.79 | 0.21 |  | 51 | | 0.98 | 0.02 |  | 69 | 0.64 | 0.36 |
| AIRT | 58 | 0.81 | 0.19 |  | 56 | | 0.00 | **1.00** |  | 59 | 1.00 | 0.00 |
| AKIT | 74 | 0.99 | 0.01 |  | 49 | | 0.99 | 0.01 |  | 79 | 0.47 | 0.53 |
| AMAL | 70 | 0.71 | 0.29 |  | 51 | | 0.90 | 0.10 |  | 70 | 0.99 | 0.01 |
| AMST | 284 | 0.99 | 0.01 |  | 166 | | 0.94 | 0.06 |  | 297 | 0.78 | 0.22 |
| ANAT | 23 | 0.70 | 0.30 |  | 20 | | 0.93 | 0.08 |  | 27 | 0.61 | 0.39 |
| AUCD | 41 | 0.93 | 0.07 |  | 37 | | 0.96 | 0.04 |  | 45 | **1.00** | 0.00 |
| AUSS | 134 | 0.00 | **1.00** |  | 100 | | 0.97 | 0.04 |  | 137 | 0.86 | 0.14 |
| AUST | 27 | **1.00** | 0.00 |  | 27 | | **1.00** | 0.00 |  | 27 | 1.00 | 0.00 |
| AWSP | 34 | 0.00 | **1.00** |  | 32 | | 0.48 | 0.52 |  | 34 | 0.00 | **1.00** |
| AZWK | 30 | **1.00** | 0.00 |  | 17 | | 0.97 | 0.03 |  | 30 | 0.10 | 0.90 |
| BARB | 19 | 0.82 | 0.18 |  | 0 | | 0.00 | 0.00 |  | 19 | 0.89 | 0.11 |
| BASS combined | 73 | 0.97 | 0.03 |  | 71 | | 0.99 | 0.01 |  | 75 | 0.01 | 0.99 |
| BASS (UK) | 12 | 0.92 | 0.08 |  | 12 | | 0.96 | 0.04 |  | 14 | 0.00 | **1.00** |
| BASS (US) | 61 | 0.98 | 0.02 |  | 59 | | 0.99 | 0.01 |  | 61 | 0.02 | 0.98 |
| BBLS | 36 | 0.32 | 0.68 |  | 13 | | 0.96 | 0.04 |  | 36 | **1.00** | 0.00 |
| BEAG combined | 111 | 0.99 | 0.01 |  | 64 | | 0.95 | 0.05 |  | 117 | 0.08 | 0.92 |
| BEAG (UK) | 16 | 0.97 | 0.03 |  | 15 | | 0.90 | 0.10 |  | 18 | 0.00 | **1.00** |
| BEAG (US Field) | 53 | 0.99 | 0.01 |  | 28 | | 0.91 | 0.09 |  | 55 | 0.07 | 0.93 |
| BEAG (US Show) | 42 | **1.00** | 0.00 |  | 36 | | 0.99 | 0.01 |  | 44 | 0.13 | 0.88 |
| BEAU | 26 | 0.98 | 0.02 |  | 21 | | **1.00** | 0.00 |  | 29 | 0.74 | 0.26 |
| BEDT | 57 | **1.00** | 0.00 |  | 58 | | 0.00 | **1.00** |  | 61 | 0.31 | 0.69 |
| BELS | 46 | 0.00 | **1.00** |  | 42 | | **1.00** | 0.00 |  | 46 | **1.00** | 0.00 |
| BERD | 56 | 0.02 | 0.98 |  | 48 | | 0.97 | 0.03 |  | 60 | 0.41 | 0.59 |
| BERG | 4 | 0.13 | 0.88 |  | 2 | | 0.50 | 0.50 |  | 9 | 0.83 | 0.17 |
| BICH | 75 | 0.03 | 0.97 |  | 73 | | 0.08 | 0.92 |  | 82 | 0.79 | 0.21 |
| BIEW | 30 | 0.08 | 0.92 |  | 25 | | 0.98 | 0.02 |  | 30 | 0.93 | 0.07 |
| BLAB | 29 | **1.00** | 0.00 |  | 24 | | 0.96 | 0.04 |  | 29 | 0.67 | 0.33 |
| BLDH | 44 | **1.00** | 0.00 |  | 44 | | 0.98 | 0.02 |  | 44 | 0.00 | **1.00** |
| BMAL | 51 | 0.89 | 0.11 |  | 43 | | 0.97 | 0.03 |  | 52 | **1.00** | 0.00 |
| BMD | 17 | 0.00 | **1.00** |  | 17 | | **1.00** | 0.00 |  | 17 | 0.03 | 0.97 |
| BOER | 21 | 0.93 | 0.07 |  | 3 | | **1.00** | 0.00 |  | 23 | 0.65 | 0.35 |
| BOLO | 8 | 0.00 | **1.00** |  | 17 | | 0.12 | 0.88 |  | 20 | 0.78 | 0.23 |
| BORD | 96 | 0.13 | 0.88 |  | 73 | | 0.90 | 0.10 |  | 99 | 0.88 | 0.12 |
| BORT | 89 | 0.99 | 0.01 |  | 87 | | 0.96 | 0.04 |  | 94 | **1.00** | 0.00 |
| BORZ | 73 | 0.00 | **1.00** |  | 51 | | 0.94 | 0.06 |  | 74 | **1.00** | 0.00 |
| BOST | 68 | 0.99 | 0.01 |  | 61 | | 0.98 | 0.02 |  | 72 | 0.99 | 0.01 |
| BOUV | 46 | 0.00 | **1.00** |  | 43 | | **1.00** | 0.00 |  | 46 | **1.00** | 0.00 |
| BOX | 71 | **1.00** | 0.00 |  | 60 | | 0.96 | 0.04 |  | 78 | 0.08 | 0.92 |
| BOYK | 9 | 0.06 | 0.94 |  | 7 | | 0.71 | 0.29 |  | 10 | 0.00 | **1.00** |
| BPIC | 10 | **1.00** | 0.00 |  | 10 | | **1.00** | 0.00 |  | 11 | **1.00** | 0.00 |
| BRAC | 13 | **1.00** | 0.00 |  | 13 | | **1.00** | 0.00 |  | 13 | 0.00 | **1.00** |
| BRIA | 55 | 0.00 | **1.00** |  | 53 | | **1.00** | 0.00 |  | 55 | 0.24 | 0.76 |
| BRIT | 53 | 0.00 | **1.00** |  | 49 | | **1.00** | 0.00 |  | 53 | 0.00 | **1.00** |
| BRTR | 35 | 0.01 | 0.99 |  | 30 | | 0.97 | 0.03 |  | 36 | 0.76 | 0.24 |
| BRUS | 46 | 0.89 | 0.11 |  | 43 | | 0.97 | 0.03 |  | 49 | 0.74 | 0.26 |
| BULD | 75 | 0.99 | 0.01 |  | 52 | | 0.98 | 0.02 |  | 79 | 0.90 | 0.10 |
| BULM combined | 57 | 0.95 | 0.05 |  | 55 | | 0.95 | 0.05 |  | 61 | 0.82 | 0.18 |
| BULM (UK) | 12 | 0.96 | 0.04 |  | 13 | | 0.85 | 0.15 |  | 15 | **1.00** | 0.00 |
| BULM (US) | 45 | 0.94 | 0.06 |  | 42 | | 0.99 | 0.01 |  | 46 | 0.76 | 0.24 |
| BULT | 58 | 0.96 | 0.04 |  | 55 | | 0.92 | 0.08 |  | 63 | **1.00** | 0.00 |
| CAAN | 18 | 0.81 | 0.19 |  | 18 | | **1.00** | 0.00 |  | 18 | 0.94 | 0.06 |
| CAIR | 58 | 0.92 | 0.08 |  | 52 | | 0.93 | 0.07 |  | 63 | 0.56 | 0.44 |
| CANE | 43 | 0.97 | 0.03 |  | 31 | | 0.95 | 0.05 |  | 46 | 0.20 | 0.80 |
| CARD | 52 | 0.70 | 0.30 |  | 48 | | 0.99 | 0.01 |  | 53 | 0.87 | 0.13 |
| CASD | 25 | 0.78 | 0.22 |  | 25 | | **1.00** | 0.00 |  | 25 | 0.44 | 0.56 |
| CAUC | 35 | 0.29 | 0.71 |  | 35 | | **1.00** | 0.00 |  | 35 | 0.11 | 0.89 |
| CCRT | 79 | 0.02 | 0.98 |  | 39 | | **1.00** | 0.00 |  | 79 | 0.83 | 0.17 |
| CESK | 13 | **1.00** | 0.00 |  | 14 | | 0.82 | 0.18 |  | 17 | **1.00** | 0.00 |
| CHIH | 52 | 0.48 | 0.52 |  | 53 | | 0.93 | 0.07 |  | 55 | 0.92 | 0.08 |
| CHIN | 49 | 0.00 | **1.00** |  | 45 | | **1.00** | 0.00 |  | 50 | 0.80 | 0.20 |
| CHOW | 54 | 0.39 | 0.61 |  | 50 | | 0.95 | 0.05 |  | 56 | 0.79 | 0.21 |
| CIRN | 13 | **1.00** | 0.00 |  | 11 | | **1.00** | 0.00 |  | 16 | **1.00** | 0.00 |
| CKCS | 121 | 0.00 | **1.00** |  | 110 | | 0.96 | 0.04 |  | 135 | 0.00 | **1.00** |
| CLSP | 57 | 0.00 | **1.00** |  | 55 | | **1.00** | 0.00 |  | 57 | 0.00 | **1.00** |
| COLL combined | 87 | 0.23 | 0.77 |  | 74 | | 0.95 | 0.05 |  | 93 | **1.00** | 0.00 |
| COLL (UK) | 21 | 0.00 | **1.00** |  | 15 | 0.83 | | 0.17 |  | 27 | **1.00** | 0.00 |
| COLL (US) | 66 | 0.30 | 0.70 |  | 59 | 0.98 | | 0.02 |  | 66 | **1.00** | 0.00 |
| COOK | 36 | 0.88 | 0.13 |  | 35 | **1.00** | | 0.00 |  | 36 | 0.72 | 0.28 |
| COTO | 77 | 0.03 | 0.97 |  | 36 | 0.88 | | 0.13 |  | 84 | 0.93 | 0.07 |
| COYO (Eastern) | 27 | **1.00** | 0.00 |  | 24 | **1.00** | | 0.00 |  | 29 | 0.91 | 0.09 |
| COYO (Western) | 12 | 0.79 | 0.21 |  | 11 | **1.00** | | 0.00 |  | 18 | **1.00** | 0.00 |
| CPBR | 43 | 0.95 | 0.05 |  | 42 | 0.43 | | 0.57 |  | 45 | 0.00 | 1.00 |
| CRES | 31 | 0.00 | **1.00** |  | 30 | 0.95 | | 0.05 |  | 31 | 0.60 | 0.40 |
| DACH combined | 166 | 0.59 | 0.41 |  | 143 | 0.96 | | 0.04 |  | 175 | 0.09 | 0.91 |
| DACH (Miniature Longhair) | 23 | 0.07 | 0.93 |  | 17 | 0.85 | | 0.15 |  | 25 | 0.10 | 0.90 |
| DACH (Miniature Shorthair) | 50 | 0.64 | 0.36 |  | 39 | 0.96 | | 0.04 |  | 49 | 0.13 | 0.87 |
| DACH (Miniature Wirehair) | 26 | 0.94 | 0.06 |  | 24 | 0.94 | | 0.06 |  | 27 | 0.02 | 0.98 |
| DACH (Standard Longhair) | 23 | 0.00 | **1.00** |  | 21 | 0.98 | | 0.02 |  | 24 | 0.27 | 0.73 |
| DACH (Standard Shorthair) | 18 | 0.86 | 0.14 |  | 18 | **1.00** | | 0.00 |  | 19 | 0.00 | **1.00** |
| DACH (Standard Wirehair) | 26 | 0.96 | 0.04 |  | 24 | **1.00** | | 0.00 |  | 31 | 0.00 | **1.00** |
| DALM combined | 46 | 0.98 | 0.02 |  | 43 | 0.97 | | 0.03 |  | 46 | 0.04 | 0.96 |
| DALM (UK) | 12 | 0.96 | 0.04 |  | 10 | 0.85 | | 0.15 |  | 12 | 0.00 | **1.00** |
| DALM (US) | 34 | 0.99 | 0.01 |  | 33 | **1.00** | | 0.00 |  | 34 | 0.06 | 0.94 |
| DANE | 87 | 0.97 | 0.03 |  | 69 | 0.98 | | 0.02 |  | 88 | 0.30 | 0.70 |
| DDBX | 32 | 0.98 | 0.02 |  | 23 | **1.00** | | 0.00 |  | 34 | 0.13 | 0.87 |
| DDMT | 41 | **1.00** | 0.00 |  | 39 | 0.99 | | 0.01 |  | 41 | 0.13 | 0.87 |
| DEER | 37 | 0.88 | 0.12 |  | 30 | 0.98 | | 0.02 |  | 38 | **1.00** | 0.00 |
| DING | 12 | **1.00** | 0.00 |  | 3 | **1.00** | | 0.00 |  | 12 | 0.83 | 0.17 |
| DOBP | 110 | **1.00** | 0.00 |  | 103 | 0.99 | | 0.01 |  | 115 | 0.54 | 0.46 |
| DOGO | 12 | 0.96 | 0.04 |  | 11 | 0.95 | | 0.05 |  | 14 | 0.96 | 0.04 |
| ECKR combined | 86 | 0.01 | 0.99 |  | 70 | 0.99 | | 0.01 |  | 89 | 0.03 | 0.97 |
| ECKR (Field) | 16 | 0.00 | **1.00** |  | 9 | **1.00** | | 0.00 |  | 17 | 0.18 | 0.82 |
| ECKR (Show) | 70 | 0.01 | 0.99 |  | 61 | 0.98 | | 0.02 |  | 72 | 0.00 | **1.00** |
| ESET | 54 | 0.01 | 0.99 |  | 56 | 0.94 | | 0.06 |  | 63 | 0.01 | 0.99 |
| ESSP combined | 97 | 0.01 | 0.99 |  | 79 | 0.96 | | 0.04 |  | 99 | 0.11 | 0.89 |
| ESSP (UK Field) | 31 | 0.00 | **1.00** |  | 28 | 0.91 | | 0.09 |  | 33 | 0.32 | 0.68 |
| ESSP (UK Show) | 29 | 0.02 | 0.98 |  | 16 | 0.97 | | 0.03 |  | 29 | 0.00 | **1.00** |
| ESSP (US Show) | 37 | 0.00 | **1.00** |  | 35 | **1.00** | | 0.00 |  | 37 | 0.01 | 0.99 |
| EURA | 26 | 0.15 | 0.85 |  | 26 | **1.00** | | 0.00 |  | 25 | 0.98 | 0.02 |
| FBUL | 61 | **1.00** | 0.00 |  | 58 | 0.97 | | 0.03 |  | 68 | 0.82 | 0.18 |
| FCR | 78 | 0.00 | **1.00** |  | 59 | 0.99 | | 0.01 |  | 82 | 0.14 | 0.86 |
| FIEL | 41 | 0.00 | **1.00** |  | 39 | **1.00** | | 0.00 |  | 42 | 0.00 | **1.00** |
| FLAP | 22 | 0.09 | 0.91 |  | 12 | 0.96 | | 0.04 |  | 26 | 0.90 | 0.10 |
| FOXH | 31 | 0.98 | 0.02 |  | 30 | 0.95 | | 0.05 |  | 33 | 0.15 | 0.85 |
| GALG | 25 | 0.82 | 0.18 |  | 24 | 0.96 | | 0.04 |  | 25 | 0.96 | 0.04 |
| GLEN | 15 | 0.97 | 0.03 |  | 13 | **1.00** | | 0.00 |  | 15 | 0.90 | 0.10 |
| GOLD combined | 180 | 0.00 | **1.00** |  | 154 | 0.94 | | 0.06 |  | 184 | 0.16 | 0.84 |
| GOLD (UK) | 39 | 0.00 | **1.00** |  | 30 | 0.92 | | 0.08 |  | 41 | 0.11 | 0.89 |
| GOLD (US) | 141 | 0.00 | **1.00** |  | 124 | 0.95 | | 0.05 |  | 143 | 0.17 | 0.83 |
| GORD | 45 | 0.00 | **1.00** |  | 44 | **1.00** | | 0.00 |  | 45 | 0.07 | 0.93 |
| GPIN | 24 | **1.00** | 0.00 |  | 21 | 0.76 | | 0.24 |  | 25 | **1.00** | 0.00 |
| GPYR | 50 | 0.02 | 0.98 |  | 39 | 0.99 | | 0.01 |  | 52 | 0.02 | 0.98 |
| GREY combined | 105 | 0.99 | 0.01 |  | 81 | 0.98 | | 0.02 |  | 109 | 0.99 | 0.01 |
| GREY (Racing) | 82 | 0.98 | 0.02 |  | 62 | 0.99 | | 0.01 |  | 84 | 0.98 | 0.02 |
| GREY (Show) | 23 | **1.00** | 0.00 |  | 19 | 0.95 | | 0.05 |  | 25 | **1.00** | 0.00 |
| GSD | 158 | 0.68 | 0.32 |  | 114 | 0.87 | | 0.13 |  | 162 | **1.00** | 0.00 |
| GSHP combined | 68 | 0.99 | 0.01 |  | 75 | 0.93 | | 0.07 |  | 74 | **1.00** | 0.00 |
| GSHP (UK) | 31 | 0.97 | 0.03 |  | 34 | 0.87 | | 0.13 |  | 37 | 0.00 | **1.00** |
| GSHP (US) | 37 | **1.00** | 0.00 |  | 36 | 0.99 | | 0.01 |  | 37 | 0.00 | **1.00** |
| GSNZ | 43 | 0.33 | 0.67 |  | 35 | 0.96 | | 0.04 |  | 44 | 0.47 | 0.53 |
| GSPZ (UK) | 30 | 0.02 | 0.98 |  | 30 | 0.95 | | 0.05 |  | 38 | 0.97 | 0.03 |
| GWHP | 33 | 0.91 | 0.09 |  | 26 | 0.98 | | 0.02 |  | 33 | 0.11 | 0.89 |
| HARR | 32 | **1.00** | 0.00 |  | 29 | **1.00** | | 0.00 |  | 33 | 0.00 | **1.00** |
| HAVA | 60 | 0.00 | **1.00** |  | 43 | 0.81 | | 0.19 |  | 61 | 0.58 | 0.42 |
| HUSK | 108 | 0.97 | 0.03 |  | 70 | 0.91 | | 0.09 |  | 115 | 0.99 | 0.01 |
| IBIZ | 36 | **1.00** | 0.00 |  | 25 | **1.00** | | 0.00 |  | 36 | 0.50 | 0.50 |
| ICES | 29 | 0.28 | 0.72 |  | 28 | 0.93 | | 0.07 |  | 29 | 0.71 | 0.29 |
| INCA | 21 | 0.43 | 0.57 |  | 20 | 0.73 | | 0.28 |  | 21 | 0.88 | 0.12 |
| IRIT | 33 | **1.00** | 0.00 |  | 31 | 0.98 | | 0.02 |  | 33 | **1.00** | 0.00 |
| ISET combined | 67 | 0.02 | 0.98 |  | 62 | 0.94 | | 0.06 |  | 75 | 0.01 | 0.99 |
| ISET (UK) | 19 | 0.05 | 0.95 |  | 20 | 0.85 | | 0.15 |  | 25 | 0.02 | 0.98 |
| ISET (US) | 48 | 0.01 | 0.99 |  | 42 | 0.99 | | 0.01 |  | 50 | 0.00 | **1.00** |
| ITGY | 68 | **1.00** | 0.00 |  | 60 | 0.99 | | 0.01 |  | 69 | 0.99 | 0.01 |
| IWOF | 62 | 0.97 | 0.03 |  | 44 | **1.00** | | 0.00 |  | 65 | **1.00** | 0.00 |
| IWSP | 32 | 0.00 | **1.00** |  | 32 | 0.00 | | **1.00** |  | 32 | 0.00 | **1.00** |
| JIND | 9 | **1.00** | 0.00 |  | 8 | **1.00** | | 0.00 |  | 11 | 0.64 | 0.36 |
| JSPZ | 16 | 0.03 | 0.97 |  | 15 | 0.97 | | 0.03 |  | 21 | **1.00** | 0.00 |
| KEES combined | 50 | 0.01 | 0.99 |  | 49 | 0.98 | | 0.02 |  | 54 | **1.00** | 0.00 |
| KEES (UK) | 18 | 0.00 | **1.00** |  | 14 | 0.96 | | 0.04 |  | 18 | **1.00** | 0.00 |
| KEES (US) | 32 | 0.02 | 0.98 |  | 35 | 0.99 | | 0.01 |  | 36 | **1.00** | 0.00 |
| KELP | 57 | 0.96 | 0.04 |  | 30 | 0.95 | | 0.05 |  | 60 | 0.95 | 0.05 |
| KERY | 41 | 0.54 | 0.46 |  | 37 | 0.88 | | 0.12 |  | 41 | **1.00** | 0.00 |
| KKLG | 42 | 0.95 | 0.05 |  | 23 | 0.78 | | 0.22 |  | 43 | 0.69 | 0.31 |
| KOMO | 26 | 0.00 | **1.00** |  | 26 | 0.00 | | **1.00** |  | 26 | 0.25 | 0.75 |
| KOOL | 39 | 0.67 | 0.33 |  | 33 | 0.83 | | 0.17 |  | 42 | 0.82 | 0.18 |
| KUVZ | 13 | 0.08 | 0.92 |  | 12 | 0.13 | | 0.88 |  | 15 | 0.33 | 0.67 |
| LAB combined | 367 | 0.98 | 0.02 |  | 272 | 0.95 | | 0.05 |  | 380 | 0.28 | 0.72 |
| LAB (UK Show) | 98 | 0.97 | 0.03 |  | 74 | 0.85 | | 0.15 |  | 107 | 0.32 | 0.68 |
| LAB (US Field) | 178 | 0.97 | 0.03 |  | 119 | 0.98 | | 0.02 |  | 180 | 0.31 | 0.69 |
| LAB (US Show) | 91 | **1.00** | 0.00 |  | 79 | 0.99 | | 0.01 |  | 93 | 0.19 | 0.81 |
| LAGO | 117 | 0.03 | 0.97 |  | 45 | 0.13 | | 0.87 |  | 139 | 0.06 | 0.94 |
| LAKE | 43 | **1.00** | 0.00 |  | 45 | 0.00 | | **1.00** |  | 45 | **1.00** | 0.00 |
| LANC | 7 | 0.71 | 0.29 |  | 6 | **1.00** | | 0.00 |  | 11 | **1.00** | 0.00 |
| LEOP | 23 | 0.96 | 0.04 |  | 21 | 0.88 | | 0.12 |  | 26 | 0.69 | 0.31 |
| LHAS combined | 57 | 0.00 | **1.00** |  | 45 | 0.94 | | 0.06 |  | 60 | 0.79 | 0.21 |
| LHAS (UK) | 16 | 0.00 | **1.00** |  | 13 | 0.85 | | 0.15 |  | 18 | 0.83 | 0.17 |
| LHAS (US) | 41 | 0.00 | **1.00** |  | 32 | 0.98 | | 0.02 |  | 42 | 0.77 | 0.23 |
| LMUN | 20 | 0.00 | **1.00** |  | 17 | 0.88 | | 0.12 |  | 21 | 0.00 | **1.00** |
| LOWC | 37 | 0.00 | **1.00** |  | 35 | 0.93 | | 0.07 |  | 37 | 0.91 | 0.09 |
| MALT combined | 88 | 0.01 | 0.99 |  | 75 | 0.94 | | 0.06 |  | 95 | 0.83 | 0.17 |
| MALT (UK) | 17 | 0.03 | 0.97 |  | 18 | 0.92 | | 0.08 |  | 21 | **1.00** | 0.00 |
| MALT (US) | 71 | 0.01 | 0.99 |  | 57 | 0.95 | | 0.05 |  | 74 | 0.78 | 0.22 |
| MANT combined | 32 | **1.00** | 0.00 |  | 30 | 0.97 | | 0.03 |  | 35 | **1.00** | 0.00 |
| MANT (UK) | 16 | **1.00** | 0.00 |  | 15 | 0.93 | | 0.07 |  | 19 | **1.00** | 0.00 |
| MANT (US) | 16 | **1.00** | 0.00 |  | 15 | **1.00** | | 0.00 |  | 16 | **1.00** | 0.00 |
| MARM | 17 | 0.00 | **1.00** |  | 20 | 0.98 | | 0.03 |  | 22 | 0.34 | 0.66 |
| MAST | 57 | 0.80 | 0.20 |  | 42 | 0.98 | | 0.02 |  | 59 | 0.11 | 0.89 |
| MBLT | 25 | **1.00** | 0.00 |  | 13 | **1.00** | | 0.00 |  | 28 | **1.00** | 0.00 |
| MCNB | 25 | 0.92 | 0.08 |  | 23 | 0.89 | | 0.11 |  | 28 | **1.00** | 0.00 |
| MGAG | 22 | 0.95 | 0.05 |  | 14 | **1.00** | | 0.00 |  | 24 | 0.92 | 0.08 |
| MIKI | 21 | 0.00 | **1.00** |  | 15 | 0.93 | | 0.07 |  | 22 | 0.52 | 0.48 |
| MPIN | 52 | 0.97 | 0.03 |  | 48 | **1.00** | | 0.00 |  | 52 | 0.98 | 0.02 |
| MSNZ | 79 | 0.89 | 0.11 |  | 56 | 0.97 | | 0.03 |  | 85 | **1.00** | 0.00 |
| MUDI | 38 | 0.05 | 0.95 |  | 33 | 0.33 | | 0.67 |  | 39 | **1.00** | 0.00 |
| NBUH | 12 | **1.00** | 0.00 |  | 10 | 0.90 | | 0.10 |  | 12 | **1.00** | 0.00 |
| NEAP | 37 | **1.00** | 0.00 |  | 32 | 0.95 | | 0.05 |  | 39 | 0.03 | 0.97 |
| NELK | 87 | **1.00** | 0.00 |  | 67 | **1.00** | | 0.00 |  | 91 | **1.00** | 0.00 |
| NEWF | 62 | 0.01 | 0.99 |  | 62 | 0.97 | | 0.03 |  | 66 | 0.05 | 0.95 |
| NLUN | 16 | **1.00** | 0.00 |  | 16 | **1.00** | | 0.00 |  | 17 | **1.00** | 0.00 |
| NORF | 44 | 0.98 | 0.02 |  | 46 | 0.96 | | 0.04 |  | 52 | **1.00** | 0.00 |
| NOWT | 61 | 0.91 | 0.09 |  | 59 | 0.98 | | 0.02 |  | 61 | **1.00** | 0.00 |
| NSDT | 36 | 0.00 | **1.00** |  | 34 | **1.00** | | 0.00 |  | 38 | 0.62 | 0.38 |
| OES | 62 | 0.02 | 0.98 |  | 41 | 0.90 | | 0.10 |  | 68 | 0.15 | 0.85 |
| OTTR | 34 | **1.00** | 0.00 |  | 29 | **1.00** | | 0.00 |  | 34 | 0.15 | 0.85 |
| PAP | 75 | 0.01 | 0.99 |  | 67 | 0.96 | | 0.04 |  | 81 | 0.57 | 0.43 |
| PBGV | 45 | **1.00** | 0.00 |  | 40 | **1.00** | | 0.00 |  | 47 | 0.00 | 1.00 |
| PDCN | 19 | **1.00** | 0.00 |  | 11 | **1.00** | | 0.00 |  | 20 | 0.93 | 0.08 |
| PEKE | 52 | 0.00 | **1.00** |  | 52 | 0.95 | | 0.05 |  | 58 | 0.23 | 0.77 |
| PEMB combined | 74 | 0.71 | 0.29 |  | 70 | 0.94 | | 0.06 |  | 75 | **1.00** | 0.00 |
| PEMB (UK) | 27 | 0.63 | 0.37 |  | 26 | 0.88 | | 0.12 |  | 28 | **1.00** | 0.00 |
| PEMB (US) | 47 | 0.76 | 0.24 |  | 44 | 0.98 | | 0.02 |  | 47 | **1.00** | 0.00 |
| PHAR | 21 | **1.00** | 0.00 |  | 20 | **1.00** | | 0.00 |  | 21 | **1.00** | 0.00 |
| PLOT | 25 | **1.00** | 0.00 |  | 24 | 0.94 | | 0.06 |  | 25 | 0.00 | **1.00** |
| PNTR | 73 | 0.99 | 0.01 |  | 75 | 0.95 | | 0.05 |  | 78 | 0.00 | **1.00** |
| POLG | 20 | 0.88 | 0.13 |  | 17 | 0.91 | | 0.09 |  | 23 | 0.85 | 0.15 |
| Poodle combined | 261 | 0.01 | 0.99 |  | 205 | 0.03 | | 0.97 |  | 281 | 0.65 | 0.35 |
| MEPOO | 15 | 0.00 | **1.00** |  | 10 | 0.00 | | **1.00** |  | 18 | 0.83 | 0.17 |
| MPOO | 125 | 0.02 | 0.98 |  | 96 | 0.05 | | 0.95 |  | 130 | 0.83 | 0.17 |
| SPOO | 80 | 0.01 | 0.99 |  | 58 | 0.02 | | 0.98 |  | 86 | 0.19 | 0.81 |
| TPOO | 41 | 0.01 | 0.99 |  | 41 | 0.00 | | **1.00** |  | 47 | 0.91 | 0.09 |
| POM combined | 86 | 0.00 | **1.00** |  | 52 | 0.95 | | 0.05 |  | 90 | 0.88 | 0.12 |
| POM (UK) | 11 | 0.00 | **1.00** |  | 8 | 0.81 | | 0.19 |  | 14 | 0.79 | 0.21 |
| POM (US) | 75 | 0.00 | **1.00** |  | 44 | 0.98 | | 0.02 |  | 76 | 0.90 | 0.10 |
| PONS | 21 | 0.00 | **1.00** |  | 20 | **1.00** | | 0.00 |  | 21 | 0.14 | 0.86 |
| PRES | 21 | 0.98 | 0.02 |  | 23 | **1.00** | | 0.00 |  | 30 | 0.45 | 0.55 |
| PRUS | 109 | 0.98 | 0.02 |  | 58 | 0.62 | | 0.38 |  | 124 | 0.96 | 0.04 |
| PTWD | 50 | 0.00 | **1.00** |  | 45 | 0.52 | | 0.48 |  | 50 | 0.18 | 0.82 |
| PUG | 58 | 0.99 | 0.01 |  | 54 | 0.99 | | 0.01 |  | 59 | **1.00** | 0.00 |
| PULI | 35 | 0.04 | 0.96 |  | 39 | 0.00 | | **1.00** |  | 39 | 0.64 | 0.36 |
| PUMI | 49 | 0.00 | **1.00** |  | 29 | 0.03 | | 0.97 |  | 51 | 1.00 | 0.00 |
| PYRS | 14 | 0.00 | **1.00** |  | 13 | **1.00** | | 0.00 |  | 14 | 0.89 | 0.11 |
| REDB | 9 | **1.00** | 0.00 |  | 8 | 0.94 | | 0.06 |  | 9 | 0.00 | **1.00** |
| RHOD | 62 | 0.98 | 0.02 |  | 53 | 0.98 | | 0.02 |  | 64 | 0.13 | 0.88 |
| ROTT | 113 | 0.88 | 0.12 |  | 95 | 0.96 | | 0.04 |  | 118 | 0.76 | 0.24 |
| RUSS | 87 | 0.96 | 0.04 |  | 53 | 0.75 | | 0.25 |  | 89 | 0.92 | 0.08 |
| RWST | 6 | 0.08 | 0.92 |  | 8 | 0.94 | | 0.06 |  | 10 | 0.00 | 1.00 |
| SALU | 116 | 0.46 | 0.54 |  | 95 | 0.98 | | 0.02 |  | 117 | 0.08 | 0.92 |
| SAMO | 18 | 0.31 | 0.69 |  | 17 | 0.91 | | 0.09 |  | 18 | **1.00** | 0.00 |
| SCOT | 62 | 0.98 | 0.02 |  | 55 | 0.93 | | 0.07 |  | 71 | **1.00** | 0.00 |
| SCWT combined | 50 | 0.01 | 0.99 |  | 43 | 0.71 | | 0.29 |  | 52 | **1.00** | 0.00 |
| SCWT (UK) | 5 | 0.10 | 0.90 |  | 2 | 0.50 | | 0.50 |  | 5 | **1.00** | 0.00 |
| SCWT (US) | 45 | 0.07 | 0.93 |  | 41 | 0.72 | | 0.28 |  | 47 | **1.00** | 0.00 |
| SEAL | 29 | **1.00** | 0.00 |  | 27 | 0.98 | | 0.02 |  | 29 | **1.00** | 0.00 |
| SFOX | 45 | 0.99 | 0.01 |  | 43 | 0.84 | | 0.16 |  | 47 | **1.00** | 0.00 |
| SHAR | 61 | 0.88 | 0.12 |  | 55 | 0.95 | | 0.05 |  | 63 | 0.88 | 0.12 |
| SHIB | 64 | 0.92 | 0.08 |  | 47 | 0.99 | | 0.01 |  | 65 | 0.97 | 0.03 |
| SHIH | 88 | 0.01 | 0.99 |  | 63 | 0.96 | | 0.04 |  | 90 | 0.65 | 0.35 |
| SILK | 45 | 0.79 | 0.21 |  | 42 | 0.98 | | 0.02 |  | 46 | 0.91 | 0.09 |
| SKIP combined | 46 | 0.82 | 0.08 |  | 43 | **1.00** | | 0.00 |  | 50 | 0.89 | 0.11 |
| SKIP (UK) | 3 | 0.83 | 0.17 |  | 4 | **1.00** | | 0.00 |  | 6 | 0.92 | 0.08 |
| SKIP (US) | 43 | 0.92 | 0.08 |  | 39 | **1.00** | | 0.00 |  | 44 | 0.89 | 0.11 |
| SKYE | 11 | 0.05 | 0.95 |  | 7 | 0.86 | | 0.14 |  | 11 | **1.00** | 0.00 |
| SLOU | 21 | 0.95 | 0.05 |  | 20 | **1.00** | | 0.00 |  | 21 | 0.05 | 0.95 |
| SMUN | 7 | 0.14 | 0.86 |  | 8 | 0.88 | | 0.13 |  | 8 | 0.06 | 0.94 |
| SPIN | 52 | 0.92 | 0.08 |  | 49 | 0.99 | | 0.01 |  | 54 | 0.00 | **1.00** |
| SPWD | 12 | 0.00 | **1.00** |  | 10 | 0.10 | | 0.90 |  | 14 | 0.14 | 0.86 |
| SSHP combined | 84 | 0.01 | 0.99 |  | 68 | 0.97 | | 0.03 |  | 90 | 0.71 | 0.29 |
| SSHP (UK) | 29 | 0.02 | 0.98 |  | 18 | 0.89 | | 0.11 |  | 34 | 0.90 | 0.10 |
| SSHP (US) | 55 | 0.00 | **1.00** |  | 50 | **1.00** | | 0.00 |  | 56 | 0.59 | 0.41 |
| SSNZ | 32 | 0.98 | 0.02 |  | 29 | **1.00** | | 0.00 |  | 32 | 0.78 | 0.22 |
| STAF | 70 | **1.00** | 0.00 |  | 55 | 0.97 | | 0.03 |  | 74 | 0.73 | 0.27 |
| SUSX | 18 | 0.00 | **1.00** |  | 14 | **1.00** | | 0.00 |  | 18 | 0.00 | **1.00** |
| SVAL | 37 | 0.99 | 0.01 |  | 33 | 0.98 | | 0.02 |  | 38 | **1.00** | 0.00 |
| TAIG | 20 | 0.40 | 0.60 |  | 21 | 0.93 | | 0.07 |  | 22 | 0.25 | 0.75 |
| TENT | 10 | 0.95 | 0.05 |  | 7 | 0.93 | | 0.07 |  | 10 | 0.95 | 0.05 |
| TERV | 39 | 0.00 | **1.00** |  | 38 | **1.00** | | 0.00 |  | 39 | **1.00** | 0.00 |
| TIBM combined | 93 | 0.51 | 0.49 |  | 56 | 0.90 | | 0.10 |  | 96 | 0.06 | 0.94 |
| TIBM (China) | 57 | 0.51 | 0.49 |  | 34 | 0.84 | | 0.16 |  | 60 | 0.05 | 0.95 |
| TIBM (US) | 36 | 0.51 | 0.49 |  | 22 | **1.00** | | 0.00 |  | 36 | 0.08 | 0.92 |
| TIBS | 47 | 0.00 | **1.00** |  | 45 | **1.00** | | 0.00 |  | 49 | 0.48 | 0.52 |
| TIBT | 64 | 0.01 | 0.99 |  | 61 | 0.97 | | 0.03 |  | 69 | 0.75 | 0.25 |
| TMNT | 37 | **1.00** | 0.00 |  | 35 | 0.99 | | 0.01 |  | 39 | **1.00** | 0.00 |
| TREE | 44 | 0.98 | 0.02 |  | 35 | 0.97 | | 0.03 |  | 45 | 0.07 | 0.93 |
| VIZS combined | 88 | 0.94 | 0.06 |  | 76 | 0.97 | | 0.03 |  | 92 | 0.03 | 0.97 |
| VIZS (UK) | 24 | 0.88 | 0.13 |  | 19 | 0.92 | | 0.08 |  | 28 | 0.02 | 0.98 |
| VIZS (US) | 64 | 0.97 | 0.03 |  | 57 | 0.99 | | 0.01 |  | 64 | 0.04 | 0.96 |
| VPIN | 18 | 0.00 | **1.00** |  | 19 | 0.95 | | 0.05 |  | 19 | 0.79 | 0.21 |
| WEIM combined | 59 | 0.96 | 0.04 |  | 56 | 0.96 | | 0.04 |  | 64 | 0.01 | 0.99 |
| WEIM (UK) | 18 | 0.94 | 0.06 |  | 17 | 0.88 | | 0.12 |  | 21 | 0.00 | **1.00** |
| WEIM (US) | 41 | 0.96 | 0.04 |  | 39 | **1.00** | | 0.00 |  | 43 | 0.01 | 0.99 |
| WELT combined | 60 | 0.99 | 0.01 |  | 60 | 0.00 | | **1.00** |  | 66 | **1.00** | 0.00 |
| WELT (UK) | 16 | **1.00** | 0.00 |  | 17 | 0.00 | | **1.00** |  | 17 | **1.00** | 0.00 |
| WELT (US) | 44 | 0.99 | 0.01 |  | 48 | 0.00 | | **1.00** |  | 49 | **1.00** | 0.00 |
| WFOX | 26 | 0.98 | 0.02 |  | 28 | 0.00 | | **1.00** |  | 31 | **1.00** | 0.00 |
| WHIP combined | 71 | 0.99 | 0.01 |  | 64 | 0.97 | | 0.03 |  | 76 | **1.00** | 0.00 |
| WHIP (Racing) | 7 | **1.00** | 0.00 |  | 4 | 0.88 | | 0.13 |  | 9 | **1.00** | 0.00 |
| WHIP (UK Show) | 34 | 0.99 | 0.01 |  | 31 | 0.95 | | 0.05 |  | 37 | **1.00** | 0.00 |
| WHIP (US Show) | 30 | **1.00** | 0.00 |  | 29 | **1.00** | | 0.00 |  | 30 | **1.00** | 0.00 |
| WHPG | 20 | 0.80 | 0.20 |  | 19 | **1.00** | | 0.00 |  | 20 | 0.00 | **1.00** |
| WHWT | 73 | 0.77 | 0.23 |  | 66 | 0.92 | | 0.08 |  | 77 | 0.99 | 0.01 |
| WOLF | 11 | **1.00** | 0.00 |  | 10 | **1.00** | | 0.00 |  | 12 | 0.54 | 0.46 |
| WSSP | 50 | 0.00 | **1.00** |  | 42 | 0.96 | | 0.04 |  | 54 | 0.00 | **1.00** |
| WVIZ | 13 | 0.85 | 0.15 |  | 14 | 0.96 | | 0.04 |  | 17 | 0.00 | **1.00** |
| XOLO | 17 | 0.74 | 0.26 |  | 15 | 0.93 | | 0.07 |  | 17 | 0.62 | 0.38 |
| YORK combined | 125 | 0.54 | 0.46 |  | 63 | 0.92 | | 0.08 |  | 131 | **1.00** | 0.00 |
| YORK (UK) | 20 | 0.58 | 0.43 |  | 17 | 0.76 | | 0.24 |  | 24 | **1.00** | 0.00 |
| YORK (US) | 105 | 0.53 | 0.47 |  | 46 | 0.98 | | 0.02 |  | 107 | **1.00** | 0.00 |

**S2 Table (e).** Allele frequencies for skull shape (*BMP3*) and natural taillessness (*T*). Breeds fixed for a single allele at any gene are indicated with bold text.

|  | ***BMP3*** | | |  | ***T*** | | |
| --- | --- | --- | --- | --- | --- | --- | --- |
| **Breed** | **n** | ***Wild-type*** | ***Short Muzzle*** |  | **n** | ***Wild-type*** | ***Tailless*** |
| ABUL | 39 | 0.96 | 0.04 |  | 39 | **1.00** | 0.00 |
| ACKR | 84 | 0.99 | 0.01 |  | 84 | **1.00** | 0.00 |
| AESK | 59 | **1.00** | 0.00 |  | 55 | **1.00** | 0.00 |
| AFFN | 40 | 0.16 | 0.84 |  | 40 | **1.00** | 0.00 |
| AFGH | 69 | **1.00** | 0.00 |  | 67 | **1.00** | 0.00 |
| AIRT | 59 | **1.00** | 0.00 |  | 58 | 0.98 | 0.02 |
| AKIT | 79 | 0.97 | 0.03 |  | 78 | **1.00** | 0.00 |
| AMAL | 70 | **1.00** | 0.00 |  | 70 | **1.00** | 0.00 |
| AMST | 297 | 0.69 | 0.31 |  | 297 | **1.00** | 0.00 |
| ANAT | 27 | **1.00** | 0.00 |  | 27 | **1.00** | 0.00 |
| AUCD | 45 | 0.54 | 0.46 |  | 44 | 0.99 | 0.01 |
| AUSS | 137 | **1.00** | 0.00 |  | 136 | 0.88 | 0.13 |
| AUST | 27 | 0.87 | 0.13 |  | 27 | **1.00** | 0.00 |
| AWSP | 34 | 0.88 | 0.12 |  | 34 | **1.00** | 0.00 |
| AZWK | 30 | **1.00** | 0.00 |  | 29 | **1.00** | 0.00 |
| BARB | 19 | **1.00** | 0.00 |  | 19 | **1.00** | 0.00 |
| BASS combined | 75 | **1.00** | 0.00 |  | 75 | **1.00** | 0.00 |
| BASS (UK) | 14 | **1.00** | 0.00 |  | 14 | **1.00** | 0.00 |
| BASS (US) | 61 | **1.00** | 0.00 |  | 61 | **1.00** | 0.00 |
| BBLS | 36 | **1.00** | 0.00 |  | 36 | **1.00** | 0.00 |
| BEAG combined | 117 | 0.98 | 0.02 |  | 115 | **1.00** | 0.00 |
| BEAG (UK) | 18 | **1.00** | 0.00 |  | 18 | **1.00** | 0.00 |
| BEAG (US Field) | 55 | 0.95 | 0.05 |  | 53 | **1.00** | 0.00 |
| BEAG (US Show) | 44 | **1.00** | 0.00 |  | 44 | **1.00** | 0.00 |
| BEAU | 29 | **1.00** | 0.00 |  | 29 | **1.00** | 0.00 |
| BEDT | 61 | **1.00** | 0.00 |  | 61 | 0.99 | 0.01 |
| BELS | 46 | **1.00** | 0.00 |  | 46 | **1.00** | 0.00 |
| BERD | 60 | **1.00** | 0.00 |  | 60 | **1.00** | 0.00 |
| BERG | 9 | **1.00** | 0.00 |  | 8 | **1.00** | 0.00 |
| BICH | 82 | **1.00** | 0.00 |  | 81 | 0.99 | 0.01 |
| BIEW | 30 | 0.98 | 0.02 |  | 30 | **1.00** | 0.00 |
| BLAB | 29 | 0.98 | 0.02 |  | 29 | **1.00** | 0.00 |
| BLDH | 44 | **1.00** | 0.00 |  | 44 | **1.00** | 0.00 |
| BMAL | 52 | **1.00** | 0.00 |  | 52 | **1.00** | 0.00 |
| BMD | 17 | **1.00** | 0.00 |  | 16 | **1.00** | 0.00 |
| BOER | 23 | 0.89 | 0.11 |  | 23 | **1.00** | 0.00 |
| BOLO | 20 | 0.98 | 0.03 |  | 19 | 0.95 | 0.05 |
| BORD | 99 | **1.00** | 0.00 |  | 97 | 0.98 | 0.02 |
| BORT | 94 | 0.74 | 0.26 |  | 94 | 0.99 | 0.01 |
| BORZ | 74 | **1.00** | 0.00 |  | 74 | **1.00** | 0.00 |
| BOST | 72 | 0.03 | 0.97 |  | 72 | **1.00** | 0.00 |
| BOUV | 46 | **1.00** | 0.00 |  | 46 | **1.00** | 0.00 |
| BOX | 78 | 0.99 | 0.01 |  | 78 | 0.99 | 0.01 |
| BOYK | 10 | 0.55 | 0.45 |  | 10 | **1.00** | 0.00 |
| BPIC | 11 | **1.00** | 0.00 |  | 11 | **1.00** | 0.00 |
| BRAC | 13 | **1.00** | 0.00 |  | 13 | **1.00** | 0.00 |
| BRIA | 55 | **1.00** | 0.00 |  | 55 | **1.00** | 0.00 |
| BRIT | 53 | 0.97 | 0.03 |  | 53 | 0.96 | 0.04 |
| BRTR | 36 | 1.00 | 0.00 |  | 36 | **1.00** | 0.00 |
| BRUS | 49 | 0.01 | 0.99 |  | 49 | **1.00** | 0.00 |
| BULD | 79 | 0.02 | 0.98 |  | 79 | **1.00** | 0.00 |
| BULM combined | 61 | 0.98 | 0.02 |  | 61 | **1.00** | 0.00 |
| BULM (UK) | 15 | **1.00** | 0.00 |  | 15 | **1.00** | 0.00 |
| BULM (US) | 46 | 0.98 | 0.02 |  | 46 | **1.00** | 0.00 |
| BULT | 63 | **1.00** | 0.00 |  | 63 | 0.98 | 0.02 |
| CAAN | 18 | **1.00** | 0.00 |  | 18 | **1.00** | 0.00 |
| CAIR | 63 | **1.00** | 0.00 |  | 62 | 0.99 | 0.01 |
| CANE | 46 | 0.96 | 0.04 |  | 45 | **1.00** | 0.00 |
| CARD | 52 | 0.72 | 0.28 |  | 53 | **1.00** | 0.00 |
| CASD | 25 | **1.00** | 0.00 |  | 25 | **1.00** | 0.00 |
| CAUC | 35 | **1.00** | 0.00 |  | 35 | **1.00** | 0.00 |
| CCRT | 79 | **1.00** | 0.00 |  | 79 | **1.00** | 0.00 |
| CESK | 17 | 0.65 | 0.35 |  | 16 | **1.00** | 0.00 |
| CHIH | 55 | 0.84 | 0.16 |  | 54 | 0.98 | 0.02 |
| CHIN | 50 | 0.03 | 0.97 |  | 50 | **1.00** | 0.00 |
| CHOW | 56 | **1.00** | 0.00 |  | 54 | **1.00** | 0.00 |
| CIRN | 16 | **1.00** | 0.00 |  | 16 | **1.00** | 0.00 |
| CKCS | 135 | **1.00** | 0.00 |  | 135 | **1.00** | 0.00 |
| CLSP | 57 | **1.00** | 0.00 |  | 57 | **1.00** | 0.00 |
| COLL combined | 93 | **1.00** | 0.00 |  | 93 | **1.00** | 0.00 |
| COLL (UK) | 27 | **1.00** | 0.00 |  | 27 | **1.00** | 0.00 |
| COLL (US) | 66 | **1.00** | 0.00 |  | 66 | **1.00** | 0.00 |
| COOK | 36 | **1.00** | 0.00 |  | 36 | **1.00** | 0.00 |
| COTO | 84 | 0.98 | 0.02 |  | 82 | 0.99 | 0.01 |
| COYO (Eastern) | 29 | **1.00** | 0.00 |  | 29 | **1.00** | 0.00 |
| COYO (Western) | 19 | **1.00** | 0.00 |  | 19 | **1.00** | 0.00 |
| CPBR | 45 | **1.00** | 0.00 |  | 45 | **1.00** | 0.00 |
| CRES | 31 | 0.47 | 0.53 |  | 30 | **1.00** | 0.00 |
| DACH combined | 181 | 0.99 | 0.01 |  | 177 | 0.99 | 0.01 |
| DACH (Miniature Longhair) | 27 | **1.00** | 0.00 |  | 27 | 0.98 | 0.02 |
| DACH (Miniature Shorthair) | 52 | 0.97 | 0.03 |  | 49 | 0.98 | 0.02 |
| DACH (Miniature Wirehair) | 28 | 0.96 | 0.04 |  | 27 | **1.00** | 0.00 |
| DACH (Standard Longhair) | 24 | **1.00** | 0.00 |  | 24 | **1.00** | 0.00 |
| DACH (Standard Shorthair) | 19 | **1.00** | 0.00 |  | 19 | 0.97 | 0.03 |
| DACH (Standard Wirehair) | 31 | **1.00** | 0.00 |  | 31 | **1.00** | 0.00 |
| DALM combined | 46 | **1.00** | 0.00 |  | 46 | **1.00** | 0.00 |
| DALM (UK) | 12 | **1.00** | 0.00 |  | 12 | **1.00** | 0.00 |
| DALM (US) | 34 | **1.00** | 0.00 |  | 34 | **1.00** | 0.00 |
| DANE | 88 | **1.00** | 0.00 |  | 88 | **1.00** | 0.00 |
| DDBX | 34 | **1.00** | 0.00 |  | 34 | **1.00** | 0.00 |
| DDMT | 41 | **1.00** | 0.00 |  | 41 | **1.00** | 0.00 |
| DEER | 38 | **1.00** | 0.00 |  | 38 | **1.00** | 0.00 |
| DING | 12 | **1.00** | 0.00 |  | 12 | **1.00** | 0.00 |
| DOBP | 115 | **1.00** | 0.00 |  | 115 | **1.00** | 0.00 |
| DOGO | 14 | 0.93 | 0.07 |  | 12 | **1.00** | 0.00 |
| ECKR combined | 89 | 0.90 | 0.10 |  | 89 | **1.00** | 0.00 |
| ECKR (Field) | 17 | 0.82 | 0.18 |  | 17 | **1.00** | 0.00 |
| ECKR (Show) | 72 | 0.92 | 0.08 |  | 72 | **1.00** | 0.00 |
| ESET | 63 | **1.00** | 0.00 |  | 63 | **1.00** | 0.00 |
| ESSP combined | 99 | 0.96 | 0.04 |  | 99 | **1.00** | 0.00 |
| ESSP (UK Field) | 33 | 0.88 | 0.12 |  | 33 | **1.00** | 0.00 |
| ESSP (UK Show) | 29 | **1.00** | 0.00 |  | 29 | **1.00** | 0.00 |
| ESSP (US Show) | 37 | **1.00** | 0.00 |  | 37 | **1.00** | 0.00 |
| EURA | 26 | **1.00** | 0.00 |  | 26 | **1.00** | 0.00 |
| FBUL | 68 | 0.01 | 0.99 |  | 68 | **1.00** | 0.00 |
| FCR | 82 | **1.00** | 0.00 |  | 82 | **1.00** | 0.00 |
| FIEL | 42 | **1.00** | 0.00 |  | 42 | **1.00** | 0.00 |
| FLAP | 26 | **1.00** | 0.00 |  | 25 | **1.00** | 0.00 |
| FOXH | 33 | **1.00** | 0.00 |  | 32 | **1.00** | 0.00 |
| GALG | 25 | **1.00** | 0.00 |  | 25 | **1.00** | 0.00 |
| GLEN | 15 | 0.70 | 0.30 |  | 14 | **1.00** | 0.00 |
| GOLD combined | 184 | **1.00** | 0.00 |  | 183 | **1.00** | 0.00 |
| GOLD (UK) | 41 | **1.00** | 0.00 |  | 41 | **1.00** | 0.00 |
| GOLD (US) | 143 | **1.00** | 0.00 |  | 142 | **1.00** | 0.00 |
| GORD | 45 | **1.00** | 0.00 |  | 45 | **1.00** | 0.00 |
| GPIN | 25 | **1.00** | 0.00 |  | 25 | **1.00** | 0.00 |
| GPYR | 52 | **1.00** | 0.00 |  | 52 | **1.00** | 0.00 |
| GREY combined | 109 | **1.00** | 0.00 |  | 106 | **1.00** | 0.00 |
| GREY (Racing) | 84 | **1.00** | 0.00 |  | 81 | **1.00** | 0.00 |
| GREY (Show) | 25 | **1.00** | 0.00 |  | 25 | **1.00** | 0.00 |
| GSD | 162 | **1.00** | 0.00 |  | 162 | **1.00** | 0.00 |
| GSHP combined | 74 | **1.00** | 0.00 |  | 74 | **1.00** | 0.00 |
| GSHP (UK) | 37 | **1.00** | 0.00 |  | 37 | **1.00** | 0.00 |
| GSHP (US) | 37 | **1.00** | 0.00 |  | 37 | **1.00** | 0.00 |
| GSNZ | 44 | **1.00** | 0.00 |  | 44 | **1.00** | 0.00 |
| GSPZ combined | 46 | 0.51 | 0.49 |  | 45 | 0.99 | 0.01 |
| GSPZ (UK) | 38 | 0.45 | 0.55 |  | 37 | **1.00** | 0.00 |
| GSPZ (US) | 8 | 0.81 | 0.19 |  | 8 | 0.94 | 0.06 |
| GWHP | 33 | **1.00** | 0.00 |  | 33 | **1.00** | 0.00 |
| HARR | 33 | **1.00** | 0.00 |  | 33 | **1.00** | 0.00 |
| HAVA | 61 | 0.98 | 0.02 |  | 61 | **1.00** | 0.00 |
| HUSK | 115 | **1.00** | 0.00 |  | 112 | **1.00** | 0.00 |
| IBIZ | 36 | **1.00** | 0.00 |  | 36 | **1.00** | 0.00 |
| ICES | 29 | **1.00** | 0.00 |  | 29 | **1.00** | 0.00 |
| INCA | 21 | 0.93 | 0.07 |  | 20 | **1.00** | 0.00 |
| IRIT | 33 | 0.89 | 0.11 |  | 33 | **1.00** | 0.00 |
| ISET combined | 75 | **1.00** | 0.00 |  | 74 | **1.00** | 0.00 |
| ISET (UK) | 25 | **1.00** | 0.00 |  | 24 | **1.00** | 0.00 |
| ISET (US) | 50 | **1.00** | 0.00 |  | 50 | **1.00** | 0.00 |
| ITGY | 69 | **1.00** | 0.00 |  | 69 | 0.99 | 0.01 |
| IWOF | 65 | **1.00** | 0.00 |  | 65 | **1.00** | 0.00 |
| IWSP | 32 | **1.00** | 0.00 |  | 32 | **1.00** | 0.00 |
| JIND | 11 | **1.00** | 0.00 |  | 11 | **1.00** | 0.00 |
| JSPZ | 21 | **1.00** | 0.00 |  | 21 | **1.00** | 0.00 |
| KEES combined | 54 | **1.00** | 0.00 |  | 53 | **1.00** | 0.00 |
| KEES (UK) | 18 | **1.00** | 0.00 |  | 18 | **1.00** | 0.00 |
| KEES (US) | 36 | **1.00** | 0.00 |  | 35 | **1.00** | 0.00 |
| KELP | 60 | **1.00** | 0.00 |  | 59 | **1.00** | 0.00 |
| KERY | 41 | **1.00** | 0.00 |  | 41 | **1.00** | 0.00 |
| KKLG | 43 | **1.00** | 0.00 |  | 43 | **1.00** | 0.00 |
| KOMO | 26 | **1.00** | 0.00 |  | 26 | **1.00** | 0.00 |
| KOOL | 42 | **1.00** | 0.00 |  | 42 | **1.00** | 0.00 |
| KUVZ | 15 | **1.00** | 0.00 |  | 14 | **1.00** | 0.00 |
| LAB combined | 380 | **1.00** | 0.00 |  | 379 | **1.00** | 0.00 |
| LAB (UK Show) | 107 | **1.00** | 0.00 |  | 107 | **1.00** | 0.00 |
| LAB (US Field) | 180 | **1.00** | 0.00 |  | 179 | **1.00** | 0.00 |
| LAB (US Show) | 93 | **1.00** | 0.00 |  | 93 | **1.00** | 0.00 |
| LAGO | 139 | **1.00** | 0.00 |  | 137 | **1.00** | 0.00 |
| LAKE | 45 | 0.07 | 0.93 |  | 45 | **1.00** | 0.00 |
| LANC | 11 | 0.59 | 0.41 |  | 9 | **1.00** | 0.00 |
| LEOP | 26 | 0.88 | 0.12 |  | 26 | **1.00** | 0.00 |
| LHAS combined | 60 | 0.79 | 0.21 |  | 59 | 0.99 | 0.01 |
| LHAS (UK) | 18 | 0.75 | 0.25 |  | 17 | 0.97 | 0.03 |
| LHAS (US) | 42 | 0.81 | 0.19 |  | 42 | **1.00** | 0.00 |
| LMUN | 21 | **1.00** | 0.00 |  | 21 | 0.98 | 0.02 |
| LOWC | 37 | 0.92 | 0.08 |  | 37 | **1.00** | 0.00 |
| MALT combined | 95 | 0.97 | 0.03 |  | 95 | 0.99 | 0.01 |
| MALT (UK) | 21 | **1.00** | 0.00 |  | 21 | **1.00** | 0.00 |
| MALT (US) | 74 | 0.97 | 0.03 |  | 74 | 0.99 | 0.01 |
| MANT combined | 35 | **1.00** | 0.00 |  | 35 | 0.99 | 0.01 |
| MANT (UK) | 19 | **1.00** | 0.00 |  | 19 | 0.97 | 0.03 |
| MANT (US) | 16 | **1.00** | 0.00 |  | 16 | **1.00** | 0.00 |
| MARM | 22 | **1.00** | 0.00 |  | 22 | **1.00** | 0.00 |
| MAST | 59 | 0.97 | 0.03 |  | 59 | **1.00** | 0.00 |
| MBLT | 28 | **1.00** | 0.00 |  | 27 | 0.96 | 0.04 |
| MCNB | 28 | 0.98 | 0.02 |  | 27 | 0.91 | 0.09 |
| MGAG | 24 | **1.00** | 0.00 |  | 23 | **1.00** | 0.00 |
| MIKI | 21 | 0.57 | 0.43 |  | 21 | **1.00** | 0.00 |
| MPIN | 52 | 0.83 | 0.17 |  | 52 | 0.99 | 0.01 |
| MSNZ | 84 | 0.14 | 0.86 |  | 85 | **1.00** | 0.00 |
| MUDI | 39 | 0.92 | 0.08 |  | 39 | 0.94 | 0.06 |
| NBUH | 12 | **1.00** | 0.00 |  | 12 | **1.00** | 0.00 |
| NEAP | 39 | **1.00** | 0.00 |  | 38 | **1.00** | 0.00 |
| NELK | 91 | 0.99 | 0.01 |  | 91 | **1.00** | 0.00 |
| NEWF | 66 | **1.00** | 0.00 |  | 66 | 0.99 | 0.01 |
| NLUN | 17 | **1.00** | 0.00 |  | 17 | **1.00** | 0.00 |
| NORF | 52 | 0.00 | **1.00** |  | 52 | **1.00** | 0.00 |
| NOWT | 61 | 0.75 | 0.25 |  | 60 | **1.00** | 0.00 |
| NSDT | 38 | **1.00** | 0.00 |  | 38 | **1.00** | 0.00 |
| OES | 68 | **1.00** | 0.00 |  | 67 | 0.99 | 0.01 |
| OTTR | 34 | **1.00** | 0.00 |  | 34 | **1.00** | 0.00 |
| PAP | 81 | **1.00** | 0.00 |  | 80 | **1.00** | 0.00 |
| PBGV | 47 | **1.00** | 0.00 |  | 47 | **1.00** | 0.00 |
| PDCN | 20 | **1.00** | 0.00 |  | 20 | **1.00** | 0.00 |
| PEKE | 58 | 0.03 | 0.97 |  | 57 | 0.99 | 0.01 |
| PEMB combined | 75 | 0.29 | 0.71 |  | 75 | 0.97 | 0.03 |
| PEMB (UK) | 28 | 0.32 | 0.68 |  | 28 | 0.95 | 0.05 |
| PEMB (US) | 47 | 0.27 | 0.73 |  | 47 | 0.99 | 0.01 |
| PHAR | 21 | 0.90 | 0.10 |  | 21 | **1.00** | 0.00 |
| PLOT | 25 | **1.00** | 0.00 |  | 25 | **1.00** | 0.00 |
| PNTR | 78 | **1.00** | 0.00 |  | 78 | **1.00** | 0.00 |
| POLG | 23 | **1.00** | 0.00 |  | 23 | **1.00** | 0.00 |
| Poodle combined | 281 | 0.74 | 0.26 |  | 278 | 1.00 | 0.00 |
| MEPOO | 18 | 0.58 | 0.42 |  | 18 | **1.00** | 0.00 |
| MPOO | 130 | 0.70 | 0.30 |  | 128 | 1.00* | 0.00* |
| SPOO | 86 | **1.00** | 0.00 |  | 85 | **1.00** | 0.00 |
| TPOO | 47 | 0.43 | 0.57 |  | 47 | **1.00** | 0.00 |
| POM combined | 80 | 0.44 | 0.56 |  | 90 | 0.99 | 0.01 |
| POM (UK) | 14 | 0.25 | 0.75 |  | 14 | **1.00** | 0.00 |
| POM (US) | 76 | 0.41 | 0.59 |  | 76 | 0.99 | 0.01 |
| PONS | 21 | **1.00** | 0.00 |  | 21 | 0.98 | 0.02 |
| PRES | 30 | 0.77 | 0.23 |  | 29 | **1.00** | 0.00 |
| PRUS | 124 | 0.66 | 0.34 |  | 121 | **1.00** | 0.00 |
| PTWD | 50 | 0.74 | 0.26 |  | 50 | **1.00** | 0.00 |
| PUG | 59 | 0.00 | **1.00** |  | 58 | **1.00** | 0.00 |
| PULI | 39 | 0.63 | 0.37 |  | 39 | **1.00** | 0.00 |
| PUMI | 51 | 0.97 | 0.03 |  | 51 | **1.00** | 0.00 |
| PYRS | 14 | 0.96 | 0.04 |  | 14 | 0.93 | 0.07 |
| REDB | 9 | **1.00** | 0.00 |  | 9 | **1.00** | 0.00 |
| RHOD | 64 | 0.90 | 0.10 |  | 63 | **1.00** | 0.00 |
| ROTT | 118 | **1.00** | 0.00 |  | 118 | **1.00** | 0.00 |
| RUSS | 89 | 0.65 | 0.35 |  | 89 | 0.99 | 0.01 |
| RWST | 10 | **1.00** | 0.00 |  | 9 | 0.94 | 0.06 |
| SALU | 117 | **1.00** | 0.00 |  | 115 | **1.00** | 0.00 |
| SAMO | 18 | **1.00** | 0.00 |  | 17 | **1.00** | 0.00 |
| SCOT | 71 | 0.06 | 0.94 |  | 71 | 0.99 | 0.01 |
| SCWT combined | 52 | 0.94 | 0.06 |  | 52 | **1.00** | 0.00 |
| SCWT (UK) | 5 | **1.00** | 0.00 |  | 5 | **1.00** | 0.00 |
| SCWT (US) | 47 | 0.94 | 0.06 |  | 47 | **1.00** | 0.00 |
| SEAL | 29 | 0.00 | **1.00** |  | 29 | **1.00** | 0.00 |
| SFOX | 47 | 0.03 | 0.97 |  | 47 | **1.00** | 0.00 |
| SHAR | 63 | 0.97 | 0.03 |  | 63 | **1.00** | 0.00 |
| SHIB | 65 | **1.00** | 0.00 |  | 65 | **1.00** | 0.00 |
| SHIH | 90 | 0.38 | 0.62 |  | 90 | 0.99 | 0.01 |
| SILK | 46 | 0.98 | 0.02 |  | 46 | **1.00** | 0.00 |
| SKIP combined | 49 | 0.23 | 0.77 |  | 50 | 0.93 | 0.07 |
| SKIP (UK) | 6 | 0.67 | 0.33 |  | 6 | 0.83 | 0.17 |
| SKIP (US) | 43 | 0.17 | 0.83 |  | 44 | 0.94 | 0.06 |
| SKYE | 11 | **1.00** | 0.00 |  | 11 | **1.00** | 0.00 |
| SLOU | 21 | **1.00** | 0.00 |  | 19 | **1.00** | 0.00 |
| SMUN | 8 | **1.00** | 0.00 |  | 7 | **1.00** | 0.00 |
| SPIN | 54 | **1.00** | 0.00 |  | 54 | **1.00** | 0.00 |
| SPWD | 14 | 0.93 | 0.07 |  | 14 | 0.86 | 0.14 |
| SSHP combined | 90 | 0.98 | 0.02 |  | 89 | 0.99 | 0.01 |
| SSHP (UK) | 34 | **1.00** | 0.00 |  | 34 | **1.00** | 0.00 |
| SSHP (US) | 56 | 0.97 | 0.03 |  | 55 | 0.99 | 0.01 |
| SSNZ | 32 | 0.58 | 0.42 |  | 32 | **1.00** | 0.00 |
| STAF | 74 | 0.03 | 0.97 |  | 73 | 0.99 | 0.01 |
| SUSX | 18 | 0.61 | 0.39 |  | 18 | **1.00** | 0.00 |
| SVAL | 38 | **1.00** | 0.00 |  | 38 | 0.82 | 0.18 |
| TAIG | 22 | **1.00** | 0.00 |  | 22 | **1.00** | 0.00 |
| TENT | 10 | 0.65 | 0.35 |  | 10 | 0.70 | 0.30 |
| TERV | 39 | **1.00** | 0.00 |  | 39 | **1.00** | 0.00 |
| TIBM combined | 97 | 0.99 | 0.01 |  | 96 | **1.00** | 0.00 |
| TIBM (China) | 61 | 0.99 | 0.01 |  | 60 | **1.00** | 0.00 |
| TIBM (US) | 36 | 0.99 | 0.01 |  | 36 | **1.00** | 0.00 |
| TIBS | 49 | 0.73 | 0.27 |  | 49 | **1.00** | 0.00 |
| TIBT | 69 | **1.00** | 0.00 |  | 68 | **1.00** | 0.00 |
| TMNT | 39 | 0.74 | 0.26 |  | 38 | 0.99 | 0.01 |
| TREE | 45 | 0.99 | 0.01 |  | 45 | 0.99 | 0.01 |
| VIZS combined | 92 | **1.00** | 0.00 |  | 91 | 0.99 | 0.01 |
| VIZS (UK) | 28 | **1.00** | 0.00 |  | 27 | 0.98 | 0.02 |
| VIZS (US) | 64 | **1.00** | 0.00 |  | 64 | **1.00** | 0.00 |
| VPIN | 19 | 0.89 | 0.11 |  | 19 | **1.00** | 0.00 |
| WEIM combined | 64 | **1.00** | 0.00 |  | 64 | **1.00** | 0.00 |
| WEIM (UK) | 21 | **1.00** | 0.00 |  | 21 | **1.00** | 0.00 |
| WEIM (US) | 43 | **1.00** | 0.00 |  | 43 | **1.00** | 0.00 |
| WELT combined | 66 | 0.17 | 0.83 |  | 66 | 0.99 | 0.01 |
| WELT (UK) | 17 | 0.26 | 0.74 |  | 17 | **1.00** | 0.00 |
| WELT (US) | 49 | 0.13 | 0.87 |  | 49 | 0.99 | 0.01 |
| WFOX | 31 | 0.47 | 0.53 |  | 31 | **1.00** | 0.00 |
| WHIP combined | 76 | **1.00** | 0.00 |  | 76 | 0.99 | 0.01 |
| WHIP (Racing) | 9 | **1.00** | 0.00 |  | 9 | 0.94 | 0.06 |
| WHIP (UK Show) | 37 | **1.00** | 0.00 |  | 37 | **1.00** | 0.00 |
| WHIP (US Show) | 30 | **1.00** | 0.00 |  | 30 | **1.00** | 0.00 |
| WHPG | 20 | **1.00** | 0.00 |  | 20 | **1.00** | 0.00 |
| WHWT | 77 | **1.00** | 0.00 |  | 74 | **1.00** | 0.00 |
| WOLF | 12 | **1.00** | 0.00 |  | 12 | **1.00** | 0.00 |
| WSSP | 54 | **1.00** | 0.00 |  | 54 | **1.00** | 0.00 |
| WVIZ | 17 | **1.00** | 0.00 |  | 17 | **1.00** | 0.00 |
| XOLO | 17 | 0.94 | 0.06 |  | 17 | **1.00** | 0.00 |
| YORK combined | 131 | 0.99 | 0.01 |  | 80 | **1.00** | 0.00 |
| YORK (UK) | 24 | **1.00** | 0.00 |  | 23 | **1.00** | 0.00 |
| YORK (US) | 107 | 0.99 | 0.01 |  | 107 | **1.00** | 0.00 |

*One Miniature Poodle was observed to be heterozygous for *T*; presence of the *T* allele in 1 heterozygote dog out of 128 total Miniature Poodles results in a *T* allele frequency of 0.004.
