# Supplementary material for: True Colors: Commercially-acquired morphological genotypes reveal hidden allele variation among dog breeds, informing both trait ancestry and breed potential
Source: PLoS One. 2019 Oct 28;14(10):e0223995. doi: 10.1371/journal.pone.0223995 (PMC6816562; doi:10.1371/journal.pone.0223995)
Supplement: S3 Table — Breeds genotyped to have alleles that would produce phenotypes considered as a “fault” by either the American Kennel Club (AKC), Fédération Cynologique Internationale (FCI), United Kennel Club (UKC), or The Kennel Club of the UK (KC). The level of tolerance within each breed registry is designated as either not allowed (N), not preferred (n.p.), allowed (Y), or ambiguously worded (amb.). A breed not recognized by a given organization is indicated with a dash (-). Inheritance of the fault-causing allele is designated as dominant (D), recessive (R), or compound heterozygote (CH). Breed name abbreviations are as listed in S1 Table. Probabilities for producing the non-standard phenotype were calculated assuming random mating within the breed, and account for multi-gene inheritance, expression, and epistatic effects. (DOCX) [file pone.0223995.s005.docx]

**S3 Table. Unfavorable or “fault” phenotypes possible by breed and breed registry.** Breeds genotyped to have alleles that would produce phenotypes considered as a “fault” by either the American Kennel Club (AKC), Fédération Cynologique Internationale (FCI), United Kennel Club (UKC), or The Kennel Club of the UK (KC). The level of tolerance within each breed registry is designated as either not allowed (N), not preferred (n.p.), allowed (Y), or ambiguously worded (amb.). A breed not recognized by a given organization is indicated with a dash (**-**). Inheritance of the fault-causing allele is designated as dominant (D), recessive (R), or compound heterozygote (CH). Breed name abbreviations are as listed in S1 Table. Probabilities for producing the non-standard phenotype were calculated assuming random mating within the breed, and account for multi-gene inheritance, expression, and epistatic effects.

| **Breed** | **Allele** | **Freq. (%)** | **Inherit.** | **Phenotype Produced** | **Prob.** | **AKC** | **FCI** | **UKC** | **KC** |
| --- | --- | --- | --- | --- | --- | --- | --- | --- | --- |
|  |  |  |  |  |  |  |  |  |  |
|  | *ASIP*^1^ |  |  |  |  |  |  |  |  |
| BIEW | *a^y^* | 2 | D | fawn | 0.03956 | - | - | N | - |
| ESSP (UK Field) | *a^y^* | 3 | D | fawn | 0.00133 | N | N | N | N |
| TREE | *a^y^* | 2 | D | fawn | 0.03832 | n.p. | - | n.p. | - |
| YORK (US) | *a^y^* | 2 | D | fawn | 0.03960 | N | N | N | N |
| ACKR | *a^w^* | 1 | R | wolf sable | 0.00403 | N | N | N | N |
| AUSS | *a^w^* | 2 | R | wolf sable | 0.03648 | N | N | N | N |
| BMAL | *a^w^* | 5 | R | wolf sable | 0.00450 | N | N | N | N |
| CKCS | *a^w^* | 1 | R | wolf sable | 0.00716 | N | N | N | N |
| ESSP (UK Field) | *a^w^* | 3 | R | wolf sable | 0.00129 | N | N | N | N |
| ESSP (UK Show) | *a^w^* | 38 | R | wolf sable | 0.12391 | N | N | N | N |
| DANE | *a^w^* | 5 | R | wolf sable | 0.00075 | N | N | N | N |
| ROTT | *a^w^* | < 1 | R | wolf sable | < 0.01990 | N | N | N | N |
| TIBM (China) | *a^w^* | 1 | R | wolf sable | 0.01596 | N | amb. | amb. | amb. |
| TMNT | *a^w^* | 1 | R | wolf sable | 0.01990 | N | N | N | N |
| AMST | *a^t^* | 19 | R | tan points | 0.01212 | n.p. | n.p. | - | - |
| BMAL | *a^t^* | 2 | R | tan points | 0.00040 | N | N | N | N |
| BOX | *a^t^* | 1 | R | tan points | 0.00010 | N | N | N | N |
| BRAC | *a^t^* | 27 | R | tan points | 0.02126 | - | N | N | N |
| BRIA | *a^t^* | 6 | R | tan points | 0.00332 | N | N | N | N |
| BLAB^2^ | *a^t^* | 16 | R | tan points | 0.01102 | - | - | - | - |
| BULD | *a^t^* | 3 | R | tan points | 0.00106 | N | n.p. | n.p. | N |
| CANE | *a^t^* | 24 | R | tan points | 0.01820 | N | N | N | - |
| CASD | *a^t^* | 15 | R | tan points | 0.02400 | - | N | N | - |
| SHAR | *a^t^* | 2 | R | tan points | 0.00025 | N | N | N | N |
| COOK | *a^t^* | 10 | R | tan points | 0.00824 | N | - | N | - |
| CHOW | *a^t^* | 1 | R | tan points | 0.00007 | N | N | N | N |
| CIRN | *a^t^* | 4 | R | tan points | 0.00039 | N | N | N | N |
| CAUC | *a^t^* | 4 | R | tan points | 0.00199 | - | N | N | - |
| FBUL | *a^t^* | 6 | R | tan points | 0.00012 | N | N | N | N |
| DANE | *a^t^* | 3 | R | tan points | 0.00017 | N | N | N | N |
| IRIT | *a^t^* | 3 | R | tan points | 0.00090 | N | N | N | N |
| ITGY | *a^t^* | 4 | R | tan points | 0.00075 | N | N | N | N |
| KEES (UK) | *a^t^* | 3 | R | tan points | 0.00090 | N | N | N | N |
| KEES (US) | *a^t^* | 1 | R | tan points | 0.00010 | N | N | N | N |
| MGAG | *a^t^* | 13 | R | tan points | 0.00804 | - | N | N | - |
| NBUH | *a^t^* | 9 | R | tan points | 0.00179 | N | N | N | N |
| PYRS | *a^t^* | 8 | R | tan points | 0.00524 | N | N | N | n.p. |
| RBCH | *a^t^* | 14 | R | tan points | 0.01960 | N | - | N | - |
| RHOD | *a^t^* | 2 | R | tan points | 0.00040 | N | N | N | N |
| SCOT | *a^t^* | 13 | R | tan points | 0.02090 | N | N | N | N |
| SCWT (UK) | *a^t^* | 17 | R | tan points | 0.02890 | N | N | N | N |
| SCWT (US) | *a^t^* | 2 | R | tan points | 0.00039 | N | N | N | N |
| STAF | *a^t^* | 32 | R | tan points | 0.04048 | N | n.p. | n.p. | n.p. |
| SSNZ | *a^t^* | 2 | R | tan points | 0.00036 | N | N | N | N |
| TAIG | *a^t^* | 24 | R | tan points | 0.00521 | N | - | - | - |
| BULD | *a* | 1 | R | black | 0.00010 | n.p. | n.p. | n.p. | N |
| CAUC | *a* | 1 | R | black | 0.00008 | - | N | N | - |
| FBUL | *a* | 1 | R | black | 0.00008 | N | N | N | N |
| PEMB (UK) | *a* | 2 | R | black | 0.00040 | N | N | N | N |
| ROTT | *a* | < 1 | R | black | < 0.00010 | N | N | N | N |
|  | *MC1R*^3^ |  |  |  |  |  |  |  |  |
| BBLS | *E^M^* | 2 | D | black mask | 0.03859 | - | N | N | N |
| LAKE | *E^M^* | 5 | D | black mask | 0.09654 | N | amb. | amb. | amb. |
| PDCN | *E^M^* | 3 | D | black mask | 0.03632 | - | amb. | amb. | - |
| WELT (US) | *E^M^* | 8 | D | black mask | 0.15360 | N | amb. | N | N |
| MANT (UK) | *E^G^* | 3 | R | grizzle | 0.05910 | N | N | N | N |
| BULM (UK) | *E* | 13 | R | no mask | 0.01690 | n.p. | N | N | N |
| BULM (US) | *E* | 2 | R | no mask | 0.00040 | n.p. | N | N | N |
| DANE | *E* | 45 | R | no mask | 0.02341 | N | n.p. | N | N |
| KEES (US) | *E* | 4 | R | no mask | 0.00240 | N | N | N | N |
| MAST | *E* | 2 | R | no mask | 0.00040 | N | N | N | N |
| PRES | *E* | 16 | R | no mask | 0.00832 | - | n.p. | N | - |
| AUCD | *e* | 1 | R | recessive red | 0.00010 | N | N | N | N |
| BEDT | *e* | 1 | R | recessive red | 0.00010 | N | N | N | N |
| BIEW | *e* | 3 | R | recessive red | 0.00090 | - | - | N | - |
| BRTR | *e* | 1 | R | recessive red | 0.00010 | N | N | N | N |
| BOST | *e* | 1 | R | recessive red | 0.00010 | N | N | N | N |
| CAIR | *e* | 2 | R | recessive red | 0.00040 | N | N | N | N |
| DALM (UK) | *e* | 4 | R | recessive red | 0.00160 | Y | N | N | N |
| DALM (US) | *e* | 4 | R | recessive red | 0.00160 | Y | N | N | N |
| FCR | *e* | 8 | R | recessive red | 0.00640 | N | N | N | N |
| GSD | *e* | 6 | R | recessive red | 0.00360 | N | N | Y | N |
| GWHP | *e* | 2 | R | recessive red | 0.00040 | N | N | N | N |
| GORD | *e* | 9 | R | recessive red | 0.00810 | N | N | N | N |
| KEES (US) | *e* | 1 | R | recessive red | 0.00010 | N | N | N | N |
| MSNZ | *e* | 14 | R | recessive red | 0.01960 | N | Y | Y | Y |
| NEWF | *e* | 1 | R | recessive red | 0.00010 | N | N | N | N |
| PLOT | *e* | 22 | R | recessive red | 0.04840 | n.p. | - | N | - |
| SKIP (UK) | *e* | 50 | R | recessive red | 0.25000 | N | N | Y | Y |
| SVAL | *e* | 4 | R | recessive red | 0.00160 | N | N | N | N |
| TREE | *e* | 5 | R | recessive red | 0.00250 | n.p. | - | n.p. | - |
| YORK (US) | *e* | 1 | R | recessive red | 0.00010 | N | N | N | N |
|  | *CBD103*^4^ | |  |  |  |  |  |  |  |
| BOER | *K^B^* or *k^br^* | 9 | D | dominant black | 0.17183 | amb. | - | amb. | - |
| SALU | *K^B^* or *k^br^* | 1 | D | dominant black | 0.01732 | N | amb. | amb. | amb. |
| TREE | *K^B^* or *k^br^* | 1 | D | dominant black | 0.00005 | n.p. | - | n.p. | - |
| BELS | *k^y^* | 7 | R | anything other than solid | 0.00423 | N | N | N | N |
| BRTR | *k^y^* | 7 | R | anything other than solid or red | 0.00000 | N | N | Y | N |
| BOST^5^ | *k^y^* | 50 | R | tan points | 0.00010 | amb. | amb. | amb. | amb. |
| BOYK | *k^y^* | 10 | R | anything other than solid | 0.01000 | N | - | N | - |
| CCRT | *k^y^* | 4 | R | anything other than solid | 0.00159 | N | N | N | N |
| DALM (US) | *k^y^* | 4 | R | anything other than solid or red | 0.00160 | N | N | N | N |
| FCR | *k^y^* | 10 | R | anything other than solid or red | 0.00801 | N | N | N | N |
| GSHP (US) | *k^y^* | 1 | R | tan points | 0.00010 | N | Y | Y | N |
| GWHP | *k^y^* | 2 | R | anything other than solid or red | 0.00040 | N | N | N | n.p. |
| GSNZ | *k^y^* | 22 | R | anything other than black or salt/pepper | 0.02810 | N | N | N | N |
| KERY | *k^y^* | 35 | R | anything other than solid | 0.12250 | N | N | N | N |
| LAB (UK) | *k^y^* | 7 | R | anything other than solid or red | 0.00430 | N | N | N | N |
| LAB (US Field) | *k^y^* | 7 | R | anything other than solid or red | 0.00038 | N | N | N | N |
| LAB (US Show) | *k^y^* | 10 | R | anything other than solid or red | 0.00307 | N | N | N | N |
| LMUN | *k^y^* | 12 | R | anything other than solid | 0.01439 | - | N | N | N |
| MUDI | *k^y^* | 27 | R | anything other than solid or red | 0.01100 | N | N | N | - |
| NEAP^5^ | *k^y^* | 50 | R | tan points | 0.01760 | N | N | N | N |
| NEWF | *k^y^* | 2 | R | anything other than solid or red | 0.00028 | N | N | N | N |
| PDCN^5^ | *k^y^* | 78 | R | wolf sable or tan points | 0.01971 | - | N | N | - |
| PNTR | *k^y^* | 1 | R | anything other than solid or red | 0.00007 | amb. | amb. | n.p. | Y |
| MePOO | *k^y^* | 28 | R | anything other than solid or red | 0.06180 | N | N | Y | - |
| MPOO | *k^y^* | 43 | R | anything other than solid or red | 0.05106 | N | N | Y | N |
| SPOO | *k^y^* | 9 | R | anything other than solid or red | 0.00479 | N | N | Y | N |
| TPOO | *k^y^* | 11 | R | anything other than solid or red | 0.01198 | N | N | Y | N |
| PTWD | *k^y^* | 5 | R | anything other than solid | 0.00212 | N | N | N | N |
| PULI | *k^y^* | 24 | R | anything other than solid or red | 0.00084 | N | amb. | N | N |
| PUMI | *k^y^* | 30 | R | anything other than solid or red | 0.01054 | N | N | amb. | N |
| SKIP (US) | *k^y^* | 1 | R | anything other than solid | 0.00006 | N | N | N | N |
| SPWD | *k^y^* | 21 | R | anything other than solid or red | 0.03838 | N | N | N | N |
| SPIN | *k^y^* | 2 | R | anything other than solid or red | 0.00008 | N | N | N | N |
| WEIM (UK) | *k^y^* | 12 | R | anything other than solid | 0.01327 | N | N | N | N |
| WEIM (US) | *k^y^* | 5 | R | anything other than solid | 0.00251 | N | N | N | N |
| WHPG | *k^y^* | 13 | R | anything other than solid | 0.01593 | N | N | N | N |
|  | *TYRP1* |  |  |  |  |  |  |  |  |
| BOYK | *B* | 5 | D | black nose | 0.09750 | N | - | N | - |
| BRIT | *B* | 12 | D | black nose | 0.22560 | N | Y | Y | Y |
| GWHP | *B* | 2 | D | black nose | 0.04000 | N | Y | Y | Y |
| IBIZ | *B* | 1 | D | black nose | 0.01990 | N | N | N | N |
| WEIM (US) | *B* | 2 | D | black nose | 0.03920 | N | N | N | N |
| AKIT | *b^s^* | 1 | CH | brown nose | 0.00010 | N | N | amb. | N |
| AMST | *b^c^* | 17 | CH | brown nose | 0.10890 | n.p. | N | - | - |
| AMST | *b^s^* | 16 | CH | combined with *b^c^*, above |  |  |  |  |  |
| AUCD | *b^s^* | 5 | CH | brown nose | 0.00250 | N | N | N | N |
| BMAL | *b^c^* | 1 | CH | brown nose | 0.00010 | N | N | N | N |
| BERG | *b^s^* | 6 | CH | brown nose | 0.00360 | N | N | N | N |
| BICH | *b^s^* | 4 | CH | brown nose | 0.00360 | N | N | N | N |
| BIEW | *b^s^* | 3 | CH | brown nose | 0.00090 | - | - | N | - |
| BOER | *b^c^* | 8 | CH | brown nose | 0.00810 | N | - | N | - |
| BOER | *b^s^* | 2 | CH | combined with *b^c^*, above |  |  |  |  |  |
| BOLO | *b^c^* | 3 | CH | brown nose | 0.00090 | - | N | N | N |
| BORT | *b^s^* | 2 | CH | brown nose | 0.00040 | N | N | n.p. | Y |
| BOST | *b^c^* | 1 | CH | brown nose | 0.00810 | N | N | N | N |
| BOST | *b^s^* | 8 | CH | combined with *b^c^*, above |  |  |  |  |  |
| BOX | *b^s^* | 1 | CH | brown nose | 0.00010 | N | N | N | N |
| BRUS | *b^c^* | 2 | CH | brown nose | 0.00040 | N | N | N | N |
| BULT | *b^c^* | 3 | CH | brown nose | 0.00360 | N | n.p. | N | N |
| BULT | *b^s^* | 3 | CH | combined with *b^c^*, above |  |  |  |  |  |
| BULD | *b^c^* | 6 | CH | brown nose | 0.00360 | N | N | N | N |
| BULM (UK) | *b^c^* | 3 | CH | brown nose | 0.01690 | N | N | N | N |
| BULM (UK) | *b^s^* | 10 | CH | combined with *b^c^*, above |  |  |  |  |  |
| BULM (US) | *b^c^* | 3 | CH | brown nose | 0.00090 | N | N | N | N |
| CAIR | *b^s^* | 1 | CH | brown nose | 0.00010 | N | N | N | N |
| CANE | *b^c^* | 4 | CH | brown nose | 0.00160 | N | N | N | - |
| CARD | *b^s^* | 3 | CH | brown nose | 0.00090 | N | N | N | N |
| CAUC | *b^s^* | 7 | CH | brown nose | 0.00490 | - | N | N | - |
| CKCS | *b^c^* | 2 | CH | brown nose | 0.00090 | N | N | N | N |
| CKCS | *b^s^* | 1 | CH | combined with *b^c^*, above |  |  |  |  |  |
| CHOW | *b^s^* | 2 | CH | brown nose | 0.00040 | N | N | N | N |
| COLL (UK) | *b^s^* | 2 | CH | brown nose | 0.00040 | N | N | N | N |
| COTO | *b^c^* | 1 | CH | brown nose | 0.00090 | N | Y | Y | N |
| COTO | *b^s^* | 2 | CH | combined with *b^c^*, above |  |  |  |  |  |
| DOGO | *b^s^* | 7 | CH | brown nose | 0.00490 | N | N | N | - |
| FBUL | *b^s^* | 1 | CH | brown nose | 0.00010 | N | N | N | N |
| GALG | *b^s^* | 6 | CH | brown nose | 0.00360 | - | N | N | - |
| GPIN | *b^c^* | 4 | CH | brown nose | 0.00160 | N | N | N | N |
| GSD | *b^c^* | 1 | CH | brown nose | 0.00040 | N | N | n.p. | n.p. |
| GSD | *b^s^* | 1 | CH | combined with *b^c^*, above |  |  |  |  |  |
| GLEN | *b^s^* | 23 | CH | brown nose | 0.05290 | N | N | N | N |
| DANE | *b^c^* | 1 | CH | brown nose | 0.00040 | N | N | N | N |
| DANE | *b^s^* | 1 | CH | combined with *b^c^*, above |  |  |  |  |  |
| GPYR | *b^c^* | 1 | CH | brown nose | 0.00040 | N | N | N | N |
| GPYR | *b^s^* | 1 | CH | combined with *b^c^*, above |  |  |  |  |  |
| IWOF | *b^s^* | 1 | CH | brown nose | 0.00010 | N | N | N | N |
| JIND | *b^c^* | 14 | CH | brown nose | 0.01960 | - | N | N | N |
| LMUN | *b^c^* | 19 | CH | brown nose | 0.03610 | - | N | N | N |
| LHAS (US) | *b^s^* | 10 | CH | brown nose | 0.01000 | N | N | N | N |
| MALT (US) | *b^c^* | 2 | CH | brown nose | 0.00160 | N | N | N | N |
| MALT (US) | *b^s^* | 2 | CH | combined with *b^c^*, above |  |  |  |  |  |
| MSNZ | *b^s^* | 1 | CH | brown nose | 0.00090 | N | N | N | N |
| PAPI | *b^s^* | 2 | CH | brown nose | 0.00040 | N | N | N | N |
| PRUS | *b^c^* | 2 | CH | brown nose | 0.00360 | N | N | N | N |
| PRUS | *b^s^* | 7 | CH | combined with *b^c^*, above |  |  |  |  |  |
| PEKE | *b^s^* | 1 | CH | brown nose | 0.00010 | N | N | N | N |
| PEMB (UK) | *b^s^* | 2 | CH | brown nose | 0.00040 | N | N | N | N |
| PBGV | *b^s^* | 4 | CH | brown nose | 0.00160 | N | N | N | N |
| PLOT | *b^c^* | 2 | CH | brown nose | 0.00640 | N | - | N | - |
| PLOT | *b^s^* | 6 | CH | combined with *b^c^*, above |  |  |  |  |  |
| PRES | *b^c^* | 3 | CH | brown nose | 0.00090 | - | N | N | - |
| ROTT | *b^c^* | < 1 | CH | brown nose | 0.00010 | N | N | N | N |
| ROTT | *b^s^* | 1 | CH | combined with *b^c^*, above |  |  |  |  |  |
| RUSS | *b^c^* | 2 | CH | brown nose | 0.00360 | N | - | - | N |
| RUSS | *b^s^* | 4 | CH | combined with *b^c^*, above |  |  |  |  |  |
| SKIP (US) | *b^s^* | 5 | CH | brown nose | 0.00160 | N | N | N | N |
| DEER | *b^s^* | 1 | CH | brown nose | 0.00010 | N | N | N | N |
| SCOT | *b^s^* | 1 | CH | brown nose | 0.00010 | N | N | N | N |
| SILK | *b^c^* | 3 | CH | brown nose | 0.00160 | N | N | N | N |
| SILK | *b^s^* | 1 | CH | combined with *b^c^*, above |  |  |  |  |  |
| SKYE | *b^s^* | 5 | CH | brown nose | 0.00250 | N | N | N | N |
| SLOU | *b^s^* | 7 | CH | brown nose | 0.00490 | N | N | N | amb. |
| SCWT (UK) | *b^s^* | 20 | CH | brown nose | 0.04000 | N | N | N | N |
| SCWT (US) | *b^s^* | 11 | CH | brown nose | 0.01210 | N | N | N | N |
| STAF | *b^c^* | 5 | CH | brown nose | 0.00360 | N | N | N | n.p. |
| STAF | *b^s^* | 1 | CH | combined with *b^c^*, above |  |  |  |  |  |
| TIBM (China) | *b^c^* | 2 | CH | brown nose | 0.00360 | Y | N | Y | n.p. |
| TIBM (China) | *b^s^* | 4 | CH | combined with *b^c^*, above |  |  |  |  |  |
| TIBS | *b^c^* | 5 | CH | brown nose | 0.00250 | N | n.p. | n.p. | N |
| TIBT | *b^c^* | 1 | CH | brown nose | 0.00490 | N | N | N | N |
| TIBT | *b^s^* | 6 | CH | combined with *b^c^*, above |  |  |  |  |  |
| TMNT | *b^s^* | 1 | CH | brown nose | 0.00010 | N | N | N | N |
| TREE | *b^c^* | 2 | CH | brown nose | 0.00160 | N | - | N | - |
| TREE | *b^s^* | 2 | CH | combined with *b^c^*, above |  |  |  |  |  |
| VPIN | *b^s^* | 11 | CH | brown nose | 0.01210 | - | N | N | - |
| WFOX | *b^s^* | 3 | CH | brown nose | 0.00090 | N | N | N | N |
| YORK (UK) | *b^s^* | 2 | CH | brown nose | 0.00040 | N | N | N | N |
| YORK (US) | *b^c^* | 2 | CH | brown nose | 0.00810 | N | N | N | N |
| YORK (US) | *b^s^* | 2 | CH | combined with *b^c^*, above |  |  |  |  |  |
|  | *MITF* |  |  |  |  |  |  |  |  |
| BIEW | *S* | 8 | D | no spotting | 0.15360 | - | - | N | - |
| ESET | *S* | 3 | D | no spotting | 0.05910 | N | N | N | N |
| PAPI | *S* | 4 | D | no spotting | 0.07840 | N | N | N | N |
| RUSS | *S* | 4 | D | no spotting | 0.07840 | N | - | - | N |
| TENT^6^ | *S* | 5 | D | no spotting | 0.09750 | - | - | - | - |
| AUSS | *s^p^* | 6 | R | piebald | 0.00360 | N | N | N | N |
| BERD | *s^p^* | 7 | R | piebald | 0.00490 | N | N | N | N |
| BEAU | *s^p^* | 5 | R | piebald | 0.00250 | N | N | N | N |
| BELS | *s^p^* | 9 | R | piebald | 0.00810 | N | N | N | N |
| TERV | *s^p^* | 3 | R | piebald | 0.00090 | N | N | N | N |
| BRTR | *s^p^* | 4 | R | piebald | 0.00160 | N | N | N | N |
| BORD | *s^p^* | 11 | R | piebald | 0.01210 | N | N | N | N |
| BOX | *s^p^* | 39 | R | piebald | 0.15210 | N | N | N | N |
| BRIA | *s^p^* | 7 | R | piebald | 0.00490 | N | N | N | N |
| CANE | *s^p^* | 1 | R | piebald | 0.00010 | N | N | N | - |
| SHAR | *s^p^* | 6 | R | piebald | 0.00360 | N | N | N | N |
| DOBP | *s^p^* | 43 | R | piebald | 0.18490 | N | N | N | N |
| EURA | *s^p^* | 2 | R | piebald | 0.00040 | - | N | N | N |
| FLAP | *s^p^* | 14 | R | piebald | 0.01960 | N | N | N | N |
| GSNZ | *s^p^* | 3 | R | piebald | 0.00090 | N | N | N | N |
| GLEN | *s^p^* | 3 | R | piebald | 0.00090 | N | N | N | N |
| GOLD (US) | *s^p^* | < 1 | R | piebald | < 0.00010 | N | N | N | N |
| DANE | *s^p^* | 6 | R | piebald | 0.00360 | amb. | amb. | amb. | amb. |
| LAB (US Field) | *s^p^* | < 1 | R | piebald | < 0.00010 | N | N | N | N |
| MAST | *s^p^* | 10 | R | piebald | 0.01000 | N | N | N | N |
| MSNZ | *s^p^* | 4 | R | piebald | 0.00160 | N | N | N | N |
| MUDI | *s^p^* | 1 | R | piebald | 0.00010 | N | N | N | - |
| NELK | *s^p^* | 27 | R | piebald | 0.07290 | n.p. | N | N | N |
| PEMB (US) | *s^p^* | 2 | R | piebald | 0.00040 | N | N | N | N |
| PLOT | *s^p^* | 8 | R | piebald | 0.00640 | N | - | N | - |
| MePOO | *s^p^* | 8 | R | piebald | 0.00160 | N | N | Y | - |
| MPOO | *s^p^* | 18 | R | piebald | 0.03240 | N | N | Y | N |
| SPOO | *s^p^* | 6 | R | piebald | 0.00360 | N | N | Y | N |
| PULI | *s^p^* | 1 | R | piebald | 0.00010 | N | N | N | N |
| ROTT | *s^p^* | < 1 | R | piebald | < 0.00010 | N | N | N | N |
| SSHP (UK) | *s^p^* | 16 | R | piebald | 0.02560 | N | n.p. | Y | n.p. |
| SSHP (US) | *s^p^* | 6 | R | piebald | 0.00360 | N | n.p. | Y | n.p. |
| SHIB | *s^p^* | 5 | R | piebald | 0.00250 | N | N | N | N |
| SILK | *s^p^* | 1 | R | piebald | 0.00010 | N | N | N | N |
| SSNZ | *s^p^* | 17 | R | piebald | 0.02890 | N | N | N | N |
| TIBM (China) | *s^p^* | 2 | R | piebald | 0.00040 | N | N | N | n.p. |
| TIBM (US) | *s^p^* | 6 | R | piebald | 0.00360 | N | N | N | n.p. |
| VIZS (US) | *s^p^* | 28 | R | piebald | 0.07840 | N | N | N | N |
| WVIZ | *s^p^* | 3 | R | piebald | 0.00090 | N | N | N | N |
| YORK (US) | *s^p^* | 5 | R | piebald | 0.00250 | N | N | N | N |
|  | *T* |  |  |  |  |  |  |  |  |
| AIRT | *tailless* | 2 | D | absent or short tail | 0.03922 | N | N | N | Y |
| AMST | *tailless* | < 1 | D | absent or short tail | < 0.01980 | N | N | - | - |
| AUCD | *tailless* | 1 | D | absent or short tail | 0.01980 | N | N | N | N |
| BEDT | *tailless* | 1 | D | absent or short tail | 0.01980 | N | N | N | N |
| BICH | *tailless* | 1 | D | absent or short tail | 0.01980 | N | N | N | N |
| BOLO | *tailless* | 5 | D | absent or short tail | 0.09524 | - | N | N | N |
| BORD | *tailless* | 2 | D | absent or short tail | 0.03922 | N | N | N | N |
| BORT | *tailless* | 1 | D | absent or short tail | 0.01980 | N | N | N | N |
| BOX | *tailless* | 1 | D | absent or short tail | 0.01980 | N | N | N | Y |
| BULT | *tailless* | 2 | D | absent or short tail | 0.03922 | N | N | N | N |
| CAIR | *tailless* | 1 | D | absent or short tail | 0.01980 | N | N | N | N |
| CHIH | *tailless* | 2 | D | absent or short tail | 0.03922 | N | N | N | N |
| COTO | *tailless* | 1 | D | absent or short tail | 0.01980 | N | N | N | N |
| DACH (Miniature Longhair) | *tailless* | 2 | D | absent or short tail | 0.03922 | N | N | N | N |
| DACH (Miniature Shorthair) | *tailless* | 2 | D | absent or short tail | 0.03922 | N | N | N | N |
| DACH (Standard Shorthair) | *tailless* | 3 | D | absent or short tail | 0.05825 | N | N | N | N |
| GSD | *tailless* | < 1 | D | absent or short tail | < 0.01980 | N | N | N | N |
| GSNZ (US) | *tailless* | 6 | D | absent or short tail | 0.11321 | N | N | N | N |
| ISET | *tailless* | 6 | D | absent or short tail | 0.11321 | N | N | N | N |
| ITGY | *tailless* | 1 | D | absent or short tail | 0.01980 | N | N | N | N |
| LAB (UK) | *tailless* | < 1 | D | absent or short tail | < 0.01980 | N | N | N | N |
| LAGO | *tailless* | < 1 | D | absent or short tail | < 0.01980 | N | N | N | N |
| LMUN | *tailless* | 1 | D | absent or short tail | 0.01980 | N | N | N | N |
| MALT (US) | *tailless* | 1 | D | absent or short tail | 0.01980 | N | N | N | N |
| MANT | *tailless* | 3 | D | absent or short tail | 0.05825 | N | N | N | N |
| MBLT | *tailless* | 4 | D | absent or short tail | 0.07692 | N | N | N | N |
| MPIN | *tailless* | 1 | D | absent or short tail | 0.01980 | N | N | N | N |
| NEWF | *tailless* | 1 | D | absent or short tail | 0.01980 | N | N | N | N |
| PEKE | *tailless* | 1 | D | absent or short tail | 0.01980 | N | N | N | N |
| POM (US) | *tailless* | 1 | D | absent or short tail | 0.01980 | N | N | N | N |
| MPOO | *tailless* | < 1 | D | absent or short tail | < 0.01980 | N | N | N | N |
| RUSS | *tailless* | 1 | D | absent or short tail | 0.01980 | N | - | - | N |
| SHIH | *tailless* | 1 | D | absent or short tail | 0.01980 | N | N | N | N |
| STAF | *tailless* | 1 | D | absent or short tail | 0.01980 | N | N | N | N |
| TMNT | *tailless* | 1 | D | absent or short tail | 0.01980 | N | N | N | N |
| TREE | *tailless* | 1 | D | absent or short tail | 0.01980 | N | - | N | - |
| VIZS (UK) | *tailless* | 2 | D | absent or short tail | 0.03922 | N | N | N | N |
| WELT (US) | *tailless* | 1 | D | absent or short tail | 0.01980 | N | N | N | N |

^1^ *ASIP* has 4 known alleles, existing in a dominance hierarchy: *a^y^* > *a^w^* > *a^t^* > *a*.

^2^ Bull Arabs are standardized under the Australian Bull Arab Registry (<http://www.bullarab.com.au/bull-arab-standard>)

^3^ *MC1R* has 4 known alleles, existing in a dominance hierarchy: *E^M^* > *E^G^* > *E* > *e*.

^4^ *CBD103* has three known alleles, existing in a dominance hierarchy: *K^B^* > *k^br^* > *k^y^*.

^5^At the time of data collection, it was not possible to genetically distinguish the *K^B^* (dominant black) and *k^br^* (brindle) alleles. Breeds with the allowed color of brindle will show *K^B^* and *k^y^* frequencies that are not representative of the actual potential to produce solid black or clear *ASIP* phenotypes, respectively. The probability values for these breeds may be inaccurate.

^6^ Tenterfield Terriers are registered with the Australian Kennel Club (<http://ankc.org.au/Breed/Detail/103>)
